# Impact of C18 Epimerization of Indole‐ and Pyrazole‐Fused 18β‐Glycyrrhetinic Acid Derivatives on PTP1B and TCPTP Inhibitory Activity: Synthesis, In Vitro, and In Silico Studies

**DOI:** 10.1002/cmdc.202500350

**Published:** 2025-09-14

**Authors:** Ledy De‐la‐Cruz‐Martínez, Rosendo Martínez‐Arellano, Mitzi López‐Sánchez, José G. Alvarado‐Rodríguez, Jesús Martin Torres‐Valencia, David Equihua‐González, Julio‐César Almanza‐Pérez, Jaime Pérez‐Villanueva, Martín González‐Andrade, José C. Páez‐Franco, Francisco Cortés‐Benítez

**Affiliations:** ^1^ Doctorado en Ciencias Farmacéuticas, División de Ciencias Biológicas y de la Salud Universidad Autónoma Metropolitana‐Unidad Xochimilco Ciudad de México 04960 Mexico; ^2^ Laboratorio de Síntesis y Aislamiento de Sustancias Bioactivas Departamento de Sistemas Biológicos División de Ciencias Biológicas y de la Salud Universidad Autónoma Metropolitana‐Unidad Xochimilco Ciudad de México 04960 Mexico; ^3^ Laboratorio de Biosensores y Modelaje Molecular Departamento de Bioquímica Facultad de Medicina Universidad Nacional Autónoma de México Ciudad de México 04510 Mexico; ^4^ Red de apoyo a la Investigación, Universidad Nacional Autónoma de México Instituto Nacional de Ciencias Médicas y Nutrición Salvador Zubirán Ciudad de México 14080 Mexico; ^5^ Área Académica de Química Universidad Autónoma del Estado de Hidalgo Hidalgo 42184 Mexico; ^6^ Laboratorio de Farmacología Departamento de Ciencias de la Salud División de Ciencias Biológicas y de la Salud Universidad Autónoma Metropolitana‐Unidad Iztapalapa Ciudad de México 09340 Mexico

**Keywords:** Glycyrrhetinic acid, Molecular docking, Molecular dynamics simulations, Protein tyrosine phosphatase 1B, Uncompetitive inhibition

## Abstract

Protein tyrosine phosphatase 1B (PTP1B) is crucial for negatively regulating the canonical insulin and leptin signaling pathways. This enzyme is a validated target for treating various disorders, including diabetes and obesity. However, to date, no PTP1B inhibitors have been approved for use. In earlier studies, we developed two modified versions of 18β‐glycyrrhetinic acid (18β‐GA) called **FC‐114** and **FC‐122**, which showed better inhibitory PTP1B activity than ursolic acid, a well‐known inhibitor. To develop even stronger inhibitors, we looked at another compound, 18α‐glycyrrhetinic acid (18α‐GA), which is more potent than 18β‐GA. Thus, in this study, we aimed to synthesize the analogs 18epi‐FC114 (**3c**) and 18epi‐FC‐122 (**5c**). These compounds were prepared with and without the carbonyl group at C11. The results showed that converting 18β‐H to 18α‐H, as well as the absence of the 11‐carbonyl group, negatively impacted the PTP1B inhibitory activity. However, the synthesized compounds exhibited an uncompetitive type of inhibition toward PTP1B and did not inhibit the TCPTP enzyme. Molecular docking and dynamics simulations suggest that the inversion of 18β‐H pushes the 30‐COOH group away, disrupting interactions at the C‐terminal site of PTP1B_1–400_. Additionally, the absence of the 11‐carbonyl group positions the compounds unfavorably, limiting critical interactions in the same region.

## Graphical abstract

The indole derivative (FC‐114) and the N‐phenylpyrazole (FC‐122) of glycyrrhetinic acid are both potent inhibitors of the Protein Tyrosine Phosphatase 1B, with IC50 values of 0.48 μM and 1.15 μM, respectively. However, their activity decreases by up to four times when the 18β‐H position is epimerized to 18α‐H or when the carbonyl group at the C11 position is removed.

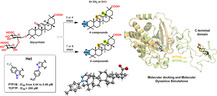

## 1 Introduction

Diabetes is a complex, multifactorial, and progressive metabolic disease characterized by elevated blood glucose levels. This condition results in postprandial hyperglycemia, characterized by abnormally high blood glucose levels after meals.^[^ ^1^, ^2^ ^–^ ^3^ ^]^ Among the three main types of diabetes (type 1, type 2, and gestational), type 2 diabetes (T2DM) is the most prevalent, accounting for over 95% of all diabetes cases worldwide.^[^ ^4^ ^]^ T2DM mainly occurs because the pancreas's beta cells do not secrete insulin effectively, and insulin‐sensitive tissues do not respond well to insulin.^[^ ^1^ ^,^ ^5^ ^]^ Since multiple factors influence T2DM and involve various molecular targets in its metabolic processes, no specific pharmaceutical target indicates a definitive treatment for this disease.^[^ ^6^ ^]^

Protein tyrosine phosphatase 1B (PTP1B) is a validated molecular target for treating T2DM and obesity‐associated comorbidities.^[^ ^7^ ^]^ Previous reports indicate that genetic suppression of PTP1B increases sensitivity to insulin and leptin hormones.^[^ ^8^ ^,^ ^9^ ^]^ PTP1B is predominantly found in essential tissues that regulate glucose metabolism, such as the liver, skeletal muscle, adipose tissue, and brain.^[^ ^10^ ^,^ ^11^ ^]^ In the insulin and leptin canonical signaling pathways, PTP1B acts as a negative regulator by dephosphorylating specific phosphotyrosine residues (pTyr) on the β subunits of the insulin receptor, as well as on Janus kinase 2 (JAK2), which is associated with the leptin receptor.^[^ ^9^ ^–^ ^11^ ^]^

Furthermore, PTP1B plays an essential role in the development of various diseases, such as inflammation, cardiovascular diseases, prostate cancer, breast cancer, neurological disorders, metabolic dysfunction‐associated steatotic liver disease (MASLD), and cellular senescence.^[^ ^12^, ^13^, ^14^ ^–^ ^15^ ^]^ Therefore, PTP1B has attracted particular attention as a therapeutic target. However, no approved PTP1B inhibitors exist to date, as most compounds that entered clinical or preclinical trials were discontinued due to their low efficacy, lack of selectivity, and resulting side effects.^[^ ^16^ ^]^

These undesirable characteristics may primarily result from the high homology of PTP1B at its catalytic site with that of other PTPs.^[^ ^17^ ^]^ Indeed, T‐cell protein tyrosine phosphatase (TCPTP) shares over 75% homology with PTP1B at the catalytic site, and its inhibition leads to serious adverse effects because TCPTP plays a crucial role in hematopoiesis and immune function. Therefore, the pursuit of allosteric inhibitors of PTP1B has become more significant and attractive during the last decade.^[^ ^17^, ^19^ ^–^ ^20^ ^]^

Several natural products have demonstrated inhibitory activity against PTP1B, such as glycyrrhetinic acid (GA). GA, a pentacyclic triterpene, is present in two epimers: 18α‐glycyrrhetinic acid (18α‐GA) and 18β‐glycyrrhetinic acid (18β‐GA). The last one is obtained by hydrolysis of glycyrrhizin, which is isolated abundantly from *Glycyrrhiza glabra* and *Glycyrrhiza uralensis* liquorice roots.^[^ ^21^ ^,^ ^22^ ^]^ Both 18β‐GA and 18α‐GA inhibit PTP1B, with 18α‐GA being more potent against it than 18β‐GA. Our research group recently reported two series of indole and *N*‐phenylpyrazole 18β‐GA derivatives. These compounds demonstrated significantly greater inhibitory activity against PTP1B compared with 18β‐GA. Additionally, they showed more potent inhibitory activity than well‐known PTP1B inhibitors such as ursolic acid, claramine, and suramin in vitro.^[^ ^23^ ^]^

**Figure 1:**
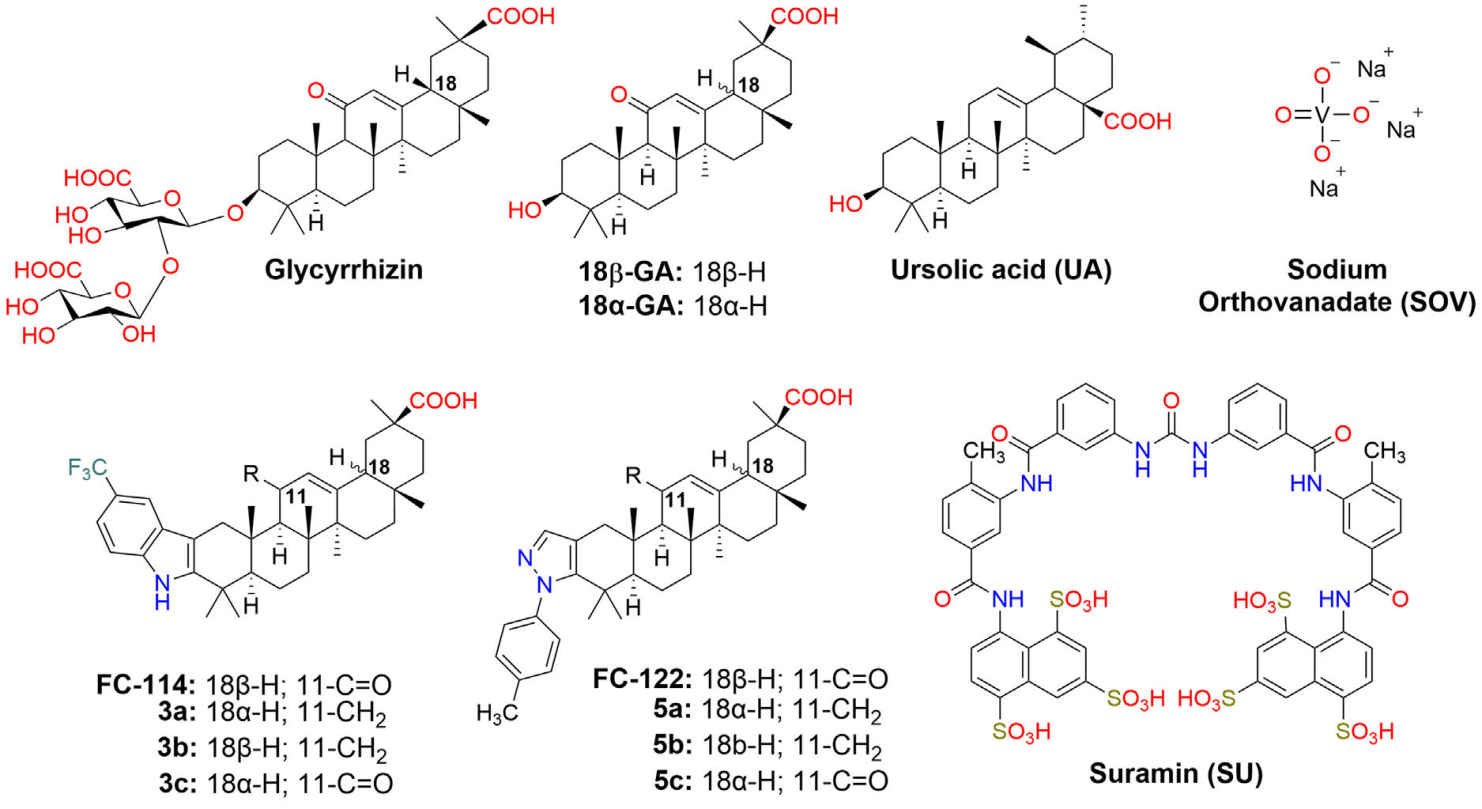
GA derivatives (**3a**‐**3c** and **5a**‐**5c**) and PTP1B inhibitors (**FC‐114**, **FC‐122**, ursolic acid, sodium orthovanadate, suramin, 18α‐GA, 18β‐GA).

In comparison with the 18β‐GA, the indole 18β‐GA derivative (**FC‐114**) was shown to be a noncompetitive inhibitor with 25 times better potency against PTP1B. Meanwhile, the *N*‐phenylpyrazole derivative (**FC‐122**) was an uncompetitive inhibitor, exhibiting 14 times greater potency against PTP1B than 18β‐GA. Both compounds were safe at doses below 2000 mg kg^−^^1^ and showed antidiabetic activity. Compound **FC‐114** significantly reduced total cholesterol levels without affecting HDL cholesterol and increased insulin levels more than glibenclamide in streptozotocin‐induced diabetic rats (**Figure** **1**).^[^ ^24^ ^]^

However, no semisynthetic derivatives of 18α‐GA have been investigated as inhibitors of PTP1B to date, despite 18α‐GA exhibiting greater potency against PTP1B than 18β‐GA. Herein, we focus on synthesizing the 18α epimers of FC‐114 (**3c**) and FC‐122 (**5c**) from 18β‐glycyrrhizin. It is worth noting that other pentacyclic triterpenes such as betulinic acid (BA), ursolic acid (UA), oleanolic acid (OA), and lupeol also demonstrate inhibitory activity against PTP1B, unlike GA, as they do not contain an α,β‐unsaturated ketone in their structures. Therefore, we also explore how removing the 11‐keto group from compounds FC‐114 (**3b**) and FC‐122 (**5b**) affects their ability to inhibit PTP1B. Since we aim to develop PTP1B inhibitors that specifically target PTP1B rather than TCPTP, as mice with suppressed TCPTP gene expression showed severe symptoms and died between weeks 3–5,^[^ ^18^ ^]^ we also assess the synthesized compounds against TCPTP.

The PTP1B inhibitory activity of the **3a**‐**3c** and **5a**‐**5c** compounds was compared with that of reference compounds: sodium orthovanadate (SOV), suramin (SU), and ursolic acid (UA). SOV is a nonselective, competitive, and reversible inhibitor of PTP1B,^[^ ^25^ ^]^ while SU is a competitive and reversible inhibitor of PTP1B used to treat trypanosomiasis and onchocerciasis.^[^ ^26^ ^]^ Finally, UA, a pentacyclic triterpene of the ursane class, inhibits PTP1B and promotes insulin receptor phosphorylation and glucose uptake in vitro.^[^ ^27^ ^]^

## 2 Results and Discussion

### 2.1 Chemistry

**Scheme 1:**
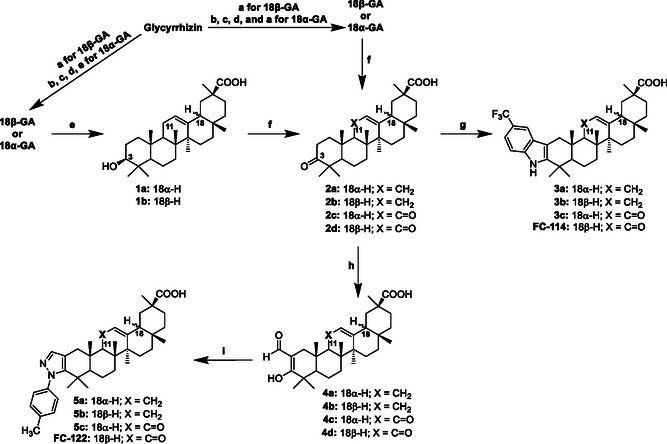
Reagents and conditions: for 18*β*‐GA a) HCl 2 M, reflux 18 h; b) KOH/H_2_O, reflux, 12 h; c) Me_2_SO_4_/K_2_CO_3_, DMSO, at room temperature; d) KOH 10% in EtOH, reflux, 3 h; e) Zn, THF, conc. HCl, 0 °C for 30 min, then at room temperature overnight; f) 2‐Iodoxybenzoic acid (IBX), DCM, room temperature, overnight or CrO_3_/H_2_SO_4_, THF, r.t., 1.5 h; g) (4‐trifluoromethyl)phenylhydrazine hydrochloride, AcOH, reflux; h) ethyl formate, NaH, THF, room temperature, overnight; i) *p*‐tolylhydrazine hydrochloride, EtOH, 85 °C.

**Scheme** **1** depicts the synthesis route for obtaining **3a–** **3c** and **5a** **–5c**. Initially, 18β‐glycyrrhizin underwent epimerization with KOH, resulting in a mixture of 18β‐glycyrrhizin and 18α‐glycyrrhizin. This mixture was then esterified with Me_2_SO_4_ in DMSO, allowing for the efficient separation of the triesters of 18β‐glycyrrhizin and 18α‐glycyrrhizin through column chromatography. Following this, recrystallization was performed in methanol.

The triester of 18α‐glycyrrhizin (yield 20%) was hydrolyzed in two steps: first, under basic conditions to obtain 18α‐glycyrrhizin, and then under acidic conditions to produce 18α‐GA. It is important to note that if hydrolysis is conducted solely in an acidic medium, the resulting product is an intermediate known as methyl‐18α‐3β‐hydroxy‐olean‐12‐en‐30‐oate. This intermediate was analyzed, and its characterization confirmed that epimerization had occurred at the asymmetric center at position C18.^[^ ^28^ ^]^

After preparation, 18α‐GA was used to synthesize the indole and *N*‐phenylpyrazole derivatives illustrated in Scheme 1. Oxidation of 18α‐GA using 2‐Iodoxybenzoic acid (IBX) at room temperature gave the ketone **2c** in 81% yield. The indole derivative of 18α‐GA (**3c**) was synthesized via Fischer indolization, with **2c** reacting with 4‐(trifluoromethyl)phenylhydrazine in refluxing acetic acid, resulting in a yield of 81% for **3c**. On the other hand, treating compound **2c** with ethyl formate in the presence of NaH produced the 1,3‐dicarbonyl compound **4c**, which was then reacted with *p*‐tolylhydrazine to give the *N*‐phenylpyrazole derivative **5c** in a 20% yield.

Scheme 1 also outlines the synthetic route for producing the indole derivatives (**3a** and **3b**) and *N*‐phenylpyrazole derivatives (**5a** and **5b**) of GA without the carbonyl group at C11. Initially, the 11‐keto group of 18α‐GA or 18β‐GA was reduced by means of a Clemmensen reaction. Subsequently, the C3 hydroxyl group (**1a** or **1b**) was oxidized to the corresponding 3‐ketone (**2a** or **2b**) using the Jones reagent in THF. This intermediate (**2a** or **2b**) was then subjected to a reaction with 4‐(trifluoromethyl)phenylhydrazine hydrochloride in refluxing acetic acid to give the indole compounds 18α‐ (**3a**) and 18β‐ (**3b**) with yields of 68% and 71%, respectively. Conversely, the compound (**2a** or **2b**) was reacted with ethyl formate to obtain the Claisen condensation product (**4a** or **4b**), which was subsequently reacted with *p*‐tolylhydrazine hydrochloride in refluxing EtOH to give the *N*‐phenylpyrazole derivatives (**5a** and **5b**) with yields of 45% and 7%, respectively.

Finally, the 18β‐epimers of the indole (**FC‐114**) and the *N*‐phenylpyrazole (**FC‐122**) were synthesized from glycyrrhizin and used as positive controls in the study. The synthesis was carried out according to the previously described methodology.^[^ ^23^ ^,^ ^24^ ^]^

### 2.2 Characterization by NMR

The formation of 18α‐glycyrrhetinic acid was confirmed through NMR characterization, utilizing both ^1^H and ^13^C NMR spectroscopy. *Jitrangsri* et al.^[^ ^28^ ^]^ reported that the 18β epimer adopts a cis conformation between its D/E rings, whereas the 18α epimer favors a *trans* conformation. This structural distinction results in a slightly higher chemical shift (2.2 ppm) for the proton at C18 in the methyl ester of 18α‐glycyrrhetinic acid, attributable to the deprotection effect of the methyl groups at positions 27 and 30, when compared to the 18β epimer, where the C18 proton appears at 2.1 ppm (Figure S12, Supporting Information). In the ^13^C NMR spectrum, the signal for C18 in the 18β epimer is observed at a higher chemical shift (48.2 ppm, Figure S13, Supporting Information), whereas for the 18α epimer, it is found at 40.1 ppm. This signal was detected through the 2D‐HSQC NMR experiment (Figure S11, Supporting Information), where it overlaps with the DMSO signal. Furthermore, the proton at C12 exhibits a slightly higher chemical shift in 18β‐GA (5.6 ppm) relative to its 18α counterpart (5.3 ppm).

The synthesis of methyl‐18α‐3β‐hydroxy‐olean‐12‐en‐30‐oate further corroborated the correct epimerization at C18. ^1^H, ^13^C, and 2D (COSY, HSQC, HMBC, and NOESY) NMR spectroscopy revealed a chemical shift of 40.5 ppm in ^13^C and 2.2 ppm in ^1^H for C18 (Figure S1–S6, Supporting Information). In addition, NOESY experiments demonstrated the absence of a correlation between proton 18‐H and methyl group 28‐CH_3_ (Figure S6, Supporting Information). In contrast, correlations were detected between the 18‐H and methyl groups 27‐CH_3_ and 30‐CH_3_, thus providing further evidence of epimerization at the C18 stereocenter, as previously reported by Jitrangsri et al.^[^ ^28^ ^]^ Consequently, it is anticipated that the final compounds **3a**, **3c**, **5a**, and **5c** will possess the proton at C18 with an alpha orientation, leading the D/E rings to adopt a *trans* conformation.

In the ^1^H NMR spectra of the indole and *N*‐phenylpyrazole derivatives **3c** and **5c** (Figure S28 and S39, Supporting Information), the vinyl proton signals at C12 are seen at 5.45 and 5.43 ppm, respectively, which are slightly lower compared to the chemical shifts of C12 in their epimers (5.52 ppm for **FC‐114** and 5.78 ppm for FC‐122, Figure S33 and S44, Supporting Information, respectively). For compound **3c**, there is a singlet at 11.23 ppm, which corresponds to the NH of the indole ring. Three signals are observed in the aromatic region (8–7.3 ppm) integrating for three hydrogens, corresponding to the indole fused to ring A of 18α‐GA, a singlet in 7.57 ppm corresponding to 4′, as well as two doublet signals at 7.43–7.27 ppm corresponding to 6′ and 7′. For the *N*‐phenylpyrazole derivative (**5c**), a singlet is observed at 7.4 ppm, corresponding to the hydrogen of the pyrazole ring, along with two doublet signals from 7.3 to 7.2 ppm with *J* = 8.24 and 7.72 Hz, corresponding to the protons of the phenyl group in **5c**. A singlet at 2.39 ppm integrating for three hydrogens corresponding to the phenyl ring's CH_3_ (4^′^) was also observed.

**Figure 2:**
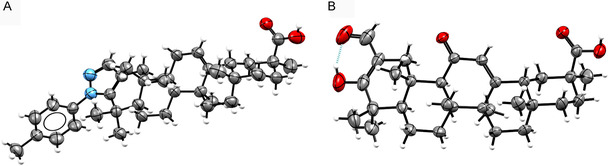
ORTEP diagram of *N‐*phenylpyrazole‐GA derivative **5b** (50% probability) A) and ORTEP diagram of intermediate **4d** (50% probability) B).

On the other hand, in the ^1^H NMR spectra of derivatives **3a**, **3b**, **5a**, and **5b** (Figure S21, S23, S36, and S38, Supporting Information, respectively), we noticed that the singlet signal of the proton at C12 shifts to lower ppm values due to the absence of the 11‐keto group (5.30, 5.27, 5.20, and 5.25 ppm, respectively), compared with **3c**, **FC‐114**, **5c**, and **FC‐122**, where the proton signal at C12 is observed at 5.45, 5.52, 5.43, and 5.78 ppm, respectively. In addition, in the ^13^C NMR spectrum of **3a**, **3b,** and **5a** (Figure S22, S24, and S37, Supporting Information, respectively), there is no longer a signal at 199.9 ppm, which corresponds to the 11‐ketone. In this sense, to corroborate the success of these reactions, a crystallographic sample of **5b** was analyzed by single‐crystal X‐ray diffraction (see Table S2, Supporting Information), which provided evidence of the removal of the carbonyl group at C11, as well as the addition of the *N*‐phenylpyrazole moiety to the ring A of 18β‐GA and demonstrated that the asymmetric carbons of the 18β‐GA skeleton remain unchanged (**Figure** **2**A).

Finally, reference compounds (**FC‐114** and **FC‐122**) were characterized and compared with those previously described.^[^ ^23^ ^,^ ^24^ ^]^ During the synthesis of the compounds mentioned above, the dicarbonyl intermediate **4d** was crystallized and then analyzed by X‐ray diffraction (see Table S1, Supporting Information and Figure 2B). Interestingly, we found that the keto functional group in C3 tautomerizes to its enol form and is stabilized by a hydrogen bond with the carbonyl oxygen in C31 belonging to the aldehyde group, resulting in the most substituted endocyclic differ from what had been previously reported by other authors, where they reported that C3 was in its keto form while C31 was in its enol form.^[^ ^23^ ^,^ ^24^ ^,^ ^29^ ^]^

### 2.3 Inhibitory Activity of PTP1B

The inhibitory activity of PTP1B was assessed using two enzyme variants: *h*PTP1B_1–285_ (the short form with an N‐terminal catalytic domain) and *h*PTP1B_1–400_ (the long form with an intrinsically disordered C‐terminal regulatory domain). This study aimed to determine the preferred binding site of GA and its derivatives within PTP1B, emphasizing the importance of using *h*PTP1B_1–400_, the representative form located in the cytosol. According to Coronel‐Tovar et al., the disordered region of the C‐terminal domain plays a crucial role in modulating PTP1B enzymatic activity.^[^ ^30^ ^]^ The newly synthesized compounds (**3a**‐**3c** and **5a**‐**5c**), starting materials (18α‐GA and 18β‐GA), **FC‐114**, **FC‐122,** and reference compounds (UA, SU, and SOV) were tested against *h*PTP1B_1–400_. Different concentrations of the compounds were evaluated to determine their IC_50_ values (**Table** **1**).

**Table 1:** Inhibitory activity of PTP1B and TCPTP by GA derivatives.

Compound	Epimer	C11position	*h*PTP1B_1–400_ IC_50_ [µM ± S.D]	*h*PTP1B_1–285_ IC_50_ [µM ± S.D]	TCPTP IC_50_[µM ± S.D]
**3a**	18α‐H	CH_2_	4.44 ± 0.22	NDa)	>100
**3b**	18β‐H	CH_2_	2.84 ± 0.26	ND	>100
**3c**	18α‐H	C=O	2.06 ± 0.08	14.90 ± 0.95	>100
**5a**	18α‐H	CH_2_	2.05 ± 0.09	ND	>100
**5b**	18β‐H	CH_2_	2.54 ± 0.05	ND	>100
**5c**	18α‐H	C=O	1.57 ± 0.04	31.59 ± 0.88	>100
**FC‐114**	18β‐H	C=O	0.48 ± 0.006	13.88 ± 0.34	>100
**FC‐122**	18β‐H	C=O	1.15 ± 0.04	25.10 ± 0.85	>100
**18α‐GA**	18α‐H	C=O	2.87 ± 0.21	ND	>100
**18β‐GA**	18β‐H	C=O	15.38 ± 0.95	ND	>100
**UA**	18β‐H	CH_2_	5.10 ± 0.34	ND	>100
**SOV**	–	–	1.19 ± 0.07	ND	0.52 ±0.053
**SU**	–	–	0.88 ± 0.09	ND	ND

a) ND: Not determined.

As expected, results showed that 18α‐GA exhibited a fivefold better inhibitory effect than 18β‐GA with IC_50_ values of 2.87 and 15.38 µM, respectively. Surprisingly, none of the newly tested derivatives proved to be more potent against *h*PTP1B_1–400_ than compounds **FC‐114** and **FC‐122**. Among the indole epimers, the beta epimer (**FC‐114**) with an IC_50_ of 0.48 μM was 4.6‐fold more potent than the alpha epimer (**3c**) with an IC_50_ of 2.06 μM. Concerning the *N*‐phenylpyrazole epimers, the beta epimer (**FC‐122**) with an IC_50_ of 1.15 μM was 1.3‐fold more potent than the alpha epimer (**5c**) with an IC_50_ of 1.57 μM. These results indicate that for these A‐ring fused indole and *N‐*phenylpyrazole derivatives, the inversion of 18β‐H to 18α‐H has a negative effect on the inhibitory activity against *h*PTP1B_1–400_. This negative impact is more significant for the indole derivatives than the *N*‐phenylpyrazole derivatives of GA. We hypothesize that this difference may be due to the fusion of the heterocycle to ring A of GA, which may cause these compounds to bind to a different region within PTP1B than that targeted by 18α‐GA and 18β‐GA.

The same trend was observed with analogs of trodusquemine, also known as MS‐1436, a potent allosteric inhibitor of PTP1B.^[^ ^31^ ^]^ *Zasloff* et al.^[^ ^32^ ^]^ conducted a study on synthesizing and evaluating analogs of this aminosteroid‐type inhibitor in obese mice. They modified a chiral center, specifically (polyamine at C3), (OH at C7), and (methyl at C20). The results indicated that the trodusquemine epimers’ inhibitors displayed poorer biological activity compared with the trodusquemine itself.^[^ ^32^ ^]^

It is important to note that all derivatives of GA, including both 18α‐ and 18β‐epimers (**3b**, **3c**, **5a**, **5b**, **5c**, **FC‐114,** and **FC‐122**), were found to be more potent against *h*PTP1B_1–400_ than their starting materials (18α‐GA and 18β‐GA). However, the indole compound **3a**, lacking 11‐carbonyl, was found to be half as potent as 18α‐GA. On the other hand, compounds **5c**, **3c**, and **5a** showed 1.8–1.4 times more potency than 18α‐GA. Interestingly, the 18β epimers (**3b** and **5b**) were 6–5.4‐fold more potent than 18β‐GA. Compared with the positive controls, the newly synthesized GA derivatives were found to be 3‐fold more potent or equipotent with UA. None of the newly synthesized GA derivatives proved to be more potent than SU or SOV.

Moreover, derivatives of GA (**3a**, **3b**, **5a**, and **5b**) without the carbonyl group at C11 showed low inhibitory activity against *h*PTP1B_1–400_ compared with those with this group (**3c**, **5c**, **FC‐114**, and **FC‐122**). This indicates that the 11‐ketone plays a crucial role in PTP1B inhibition and may act as a hydrogen bond acceptor within the enzyme. *Kyriakou* et al. (2018) found similar results, showing a decrease in the inhibitory activity of celastrol (a noncompetitive inhibitor of PTP1B) when its ketone group was reduced. These findings emphasize the significance of the carbonyl group in interacting with PTP1B.^[^ ^33^ ^]^

Subsequently, we investigated whether indole and *N*‐phenylpyrazole derivatives of GA, which contain the carbonyl group at C11 (**FC‐114**, **FC‐122**, **3c**, and **5c**), prefer binding to the long or short form of PTP1B. To do so, these terpenoids were tested against *h*PTP1B_1–285_. The results indicated that these compounds exhibited 7‐ to 28‐fold greater potency for *h*PTP1B_1–400_ over *h*PTP1B_1–285_, indicating that the intrinsically disordered C‐terminus region (PTP1B_301–400_) is significant and may play a significant role in the binding of GA derivatives.

Unlike other similar regions in PTP family proteins, the C‐terminal region of PTP1B (PTP1B_301–400_) contains two α‐helices (α8′ (residues 320–327) and α9′ (residues 360–377)) that could serve as binding sites for compounds. The ability of compounds to bind to this specific site within the disordered region makes the inhibitors more selective for PTP1B compared with other PTP proteins, such as TCPTP.^[^ ^34^ ^,^ ^35^ ^]^ A clear example of this selectivity is trodusquemine (MSI‐1436), a highly selective PTP1B inhibitor that binds directly to this site in the disordered region, demonstrating its specificity for PTP1B.^[^ ^31^ ^]^ Therefore, GA derivatives (**FC‐114**, **FC‐122**, **3a**, **3b**, **3c**, **5a**, **5b**, and **5c**) could not only inhibit PTP1B but also be selective for PTP1B over TCPTP.

### 2.4 Selectivity Assay against T‐Cell Protein Tyrosine Phosphatase (TCPTP)

The effectiveness and safety of protein tyrosine phosphatase (PTP) inhibitors depend on their specificity. PTP1B and TCPTP serve different functions: PTP1B is involved in metabolism and cancer, while TCPTP regulates immune function. Inhibiting TCPTP could impact the immune response, whereas inhibiting PTP1B may benefit metabolic diseases without these side effects.^[^ ^20^ ^,^ ^34^ ^,^ ^35^ ^]^ Thus, all the newly synthesized compounds (**3a**‐**3c** and **5a**‐**5c**), starting materials (18α‐GA and 18β‐GA), and SOV were assessed to determine their ability to inhibit TCPTP. These compounds were assessed up to 100 μM (Table 1). The results of this study showed that compounds **3a**‐**3c**, **5a**‐**5c**, 18α‐GA, and 18β‐GA do not inhibit TCPTP (Figure S51, Supporting Information). Conversely, SOV (a nonselective PTP1B inhibitor) inhibited both PTP1B and TCPTP. This indicates that starting materials (18α‐GA and 18β‐GA) and their derivatives (**3a**‐**3c** and **5a**‐**5c**) selectively inhibit PTP1B over TCPTP at the tested concentrations. Furthermore, these compounds may bind to PTP1B at or near the intrinsically disordered C‐terminal site, which is absent in TCPTP. Consequently, the GA skeleton is an attractive scaffold that could be further explored for the development of selective PTP1B inhibitors.

### 2.5 Enzymatic Kinetics Studies

**Figure 3:**
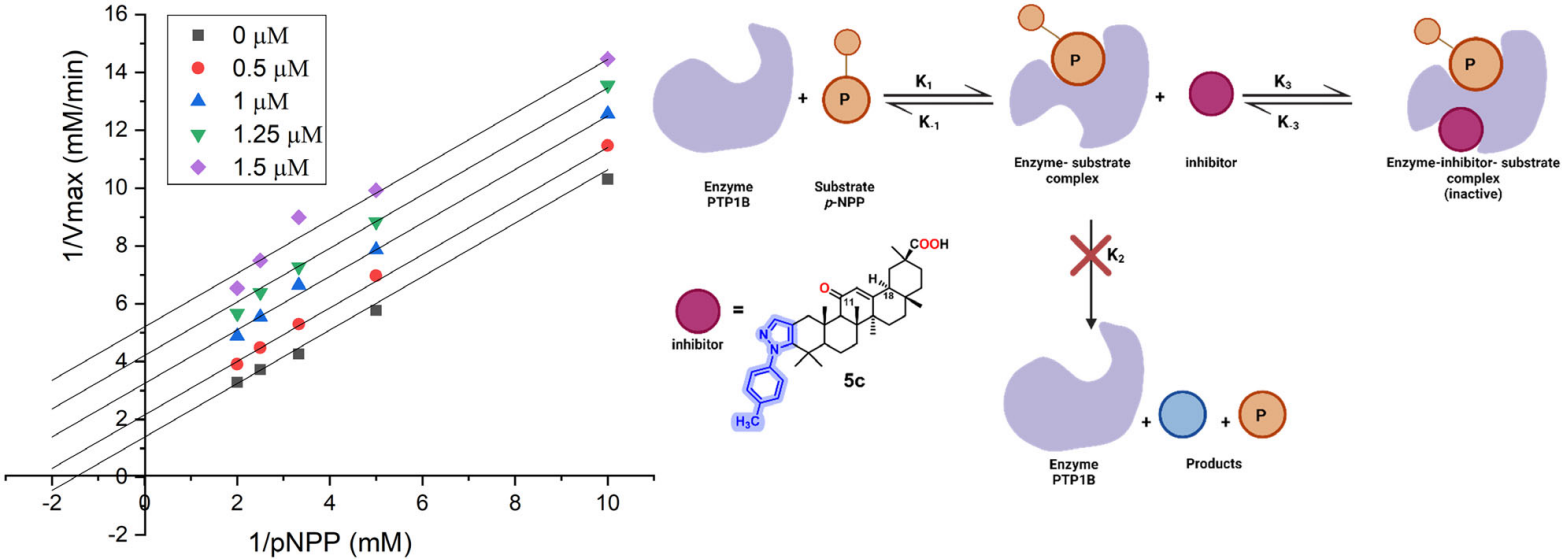
Uncompetitive inhibition model of PTP1B_1–400_ in the presence of **5c**. *p*‐NPP: p‐nitrophenyl phosphate; P: phosphate group, adapted from “uncompetitive inhibition”. Created in BioRender. Matuz Mares, D. (2025) https://BioRender.com/w69r443.

Enzymatic kinetics studies were conducted on 18α‐GA and 18β‐GA, as well as their derivatives **3b**, **3c**, and **5c**, to investigate the type of inhibition they exerted against *h*PTP1B_1–400_ (**Table** **2**). Kinetic analyses were performed at various substrate and inhibitor concentrations. All the tested compounds exhibited uncompetitive inhibition, with K_i_ values of 0.62, 1.63, 1.16, 0.071, and 0.22 µM, respectively (see Figure S52, Supporting Information). In this type of inhibition, the inhibitor binds to the enzyme–substrate complex rather than to the free enzyme^[^ ^36^ ^]^ (**Figure** **3**), allowing for greater selectivity due to the differences in amino acid residues and polarities between the allosteric sites and the active site of PTP1B. Allosteric inhibition of PTP1B can occur via two mechanisms: 1) by targeting the α3, α6, and α7 helices in the N‐terminal catalytic domain (PTP1B_1–300_) or 2) by interacting with the α8′ and α9′ helices (comprising amino acids 320–327 and 360–377, respectively) in the intrinsically disordered C‐terminal regulatory domain (PTP1B_301–400_).^[^ ^31^ ^,^ ^34^ ^,^ ^35^ ^]^ Therefore, the uncompetitive inhibition demonstrated by these compounds is significant, as their ability to bind to sites distinct from the catalytic site may enhance selectivity in interaction with *h*PTP1B_1–400_, corroborating the previously described results.

It is worth noting that previous research has demonstrated that 18α‐GA and 18β‐GA exhibit competitive inhibition against hPTP1B1−322, with respective IC_50_ values of 10.40 and 26.07 µM and *K* *_i_* values of 3.17 and 16.23 µM.^24^ In our study, we used the *h*PTP1B_1–400_ enzyme, which represents the long form of the protein and includes the intrinsically disordered C‐terminal regulatory domain, to determine the kinetic parameters of compounds **3b**, **3c**, **5c**, 18α‐GA, and 18β‐GA. This proline‐rich domain confers substrate specificity to PTP1B. Therefore, discrepancies in the observed IC_50_ and *K*_i_ values, as well as the type of inhibition, may be attributed to the specific enzyme used in those studies or to statistical adjustments, as noted by other researchers.^[^ ^35^ ^]^

**Table 2:** Kinetic parameters of *h*PTP1B_1–400_ with different inhibitors.

Compound	*K* *_i_* ± S.D[µM]	*V*_max_ ± S.D[mM min^−1^]	*K*_m_ ± S.D[mM]	Inhibition type
**3b**	1.16 ± 0.13	0.63 ± 0.06	0.53 ± 0.08	uncompetitive
**3c**	0.071 ± 0.008	0.62 ± 0.05	0.52 ± 0.07	uncompetitive
**5c**	0.224 ± 0.05	1.54 ± 0.28	1.42 ± 0.32	uncompetitive
**18α‐GA**	0.62 ± 0.19	1.94 ± 0.54	2.11 ± 0.68	uncompetitive
**18β‐GA**	1.63 ± 0.50	3.13 ± 0.94	2.83 ± 0.96	uncompetitive

### 2.6 Molecular Docking Studies

To investigate the potential binding modes of GA derivatives (**3a**, **3b**, **3c**, **5a**, **5b**, and **5c**) with PTP1B, both the short form (PTP1B_1–298_) and the long form (PTP1B_1–400_) of the protein were analyzed using molecular docking simulations. These simulations were performed using three software programs: AutoDock 4.2 (AD)^[^ ^37^ ^]^ (The Scripps Research Institute, La Jolla, CA, USA), Autodock Vina (VINA) (The Scripps Research Institute, La Jolla, CA, USA),^[^ ^38^ ^]^ and GOLD^[^ ^39^ ^]^ (The Cambridge Crystallographic Data Centre, Cambridge, UK) version 2024.1.0, along with the CB‐Dock2 web server.^[^ ^40^ ^,^ ^41^ ^]^ The 3D structure of *Homo sapiens* PTP1B_1–298_ (PDB ID: 1C83), comprising residues 1–298, as well as a modeled structure of PTP1B_1–400_ complexed with *p*‐nitrophenyl phosphate (pNPP) substrate (PTP1B_1–400_‐pNPP), was used. This study utilized the three software programs mentioned above to gather information from multiple scoring functions, identifying the most accurate solution for each protein–ligand interaction.

To pinpoint the preferred binding sites for the synthesized compounds (**3a**, **3b**, **3c**, **5a**, **5b**, and **5c**), raw materials (18α‐GA and 18β‐GA), and reference compounds (**FC‐114** and **FC‐122**) in the PTP1B_1–298_ enzyme (PDB ID: 1C83), a blind docking simulation was performed using AD and the CB‐Dock2 server. Following this, site‐directed docking simulations were carried out using AD, VINA, and GOLD at the preferred binding sites of the GA derivatives.

**Figure 4:**
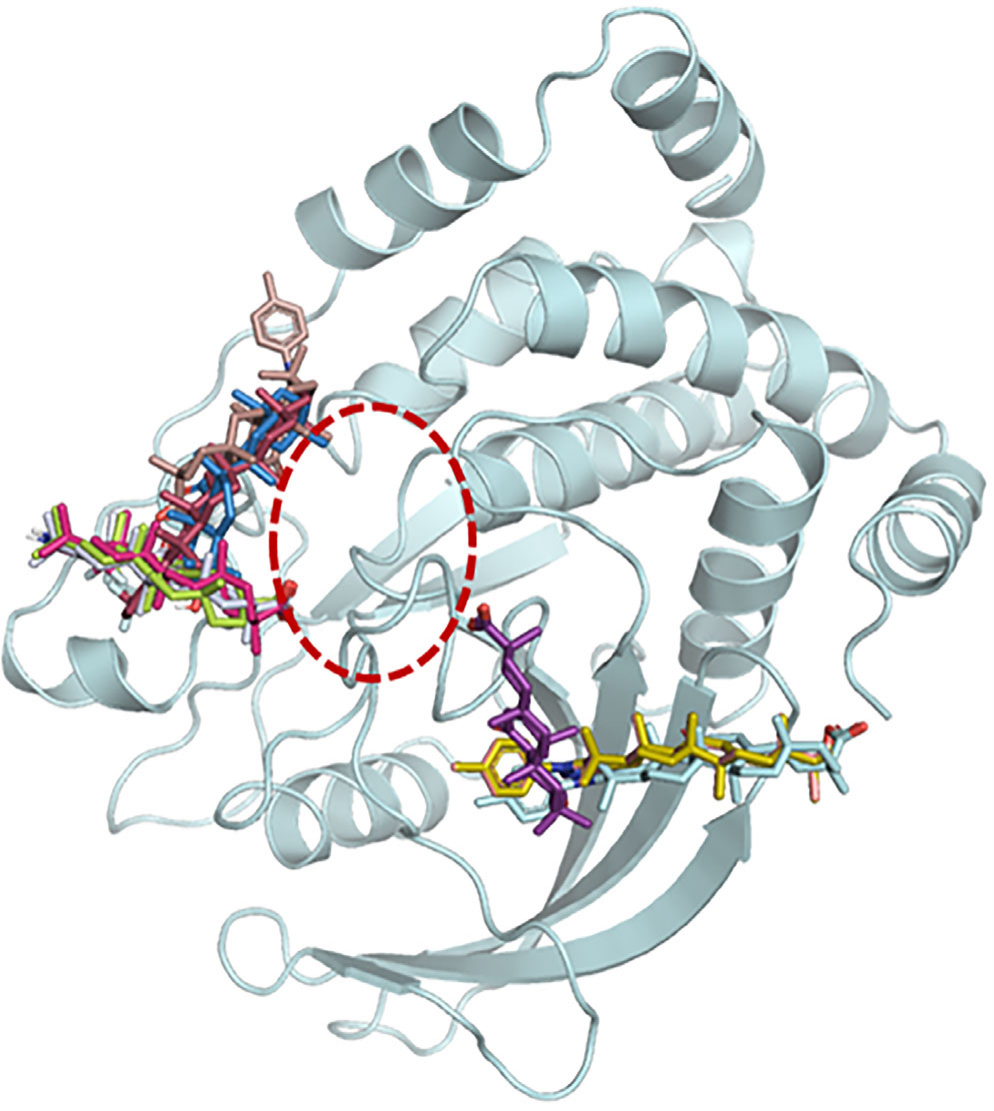
Binding mode of **3a** (red), **3b** (pink), **3c** (gray), **5a** (yellow), **5b** (cyan), **5c** (light pink), **18** **β**‐**GA** (purple), **18** **α** **‐GA** (blue), **FC‐114** (green), and **FC‐122** (brown) of blind docking of PTP1B_1–298_ (cyan). The catalytic site of PTP1B_1–298_ (red lines).

**Figure 5:**
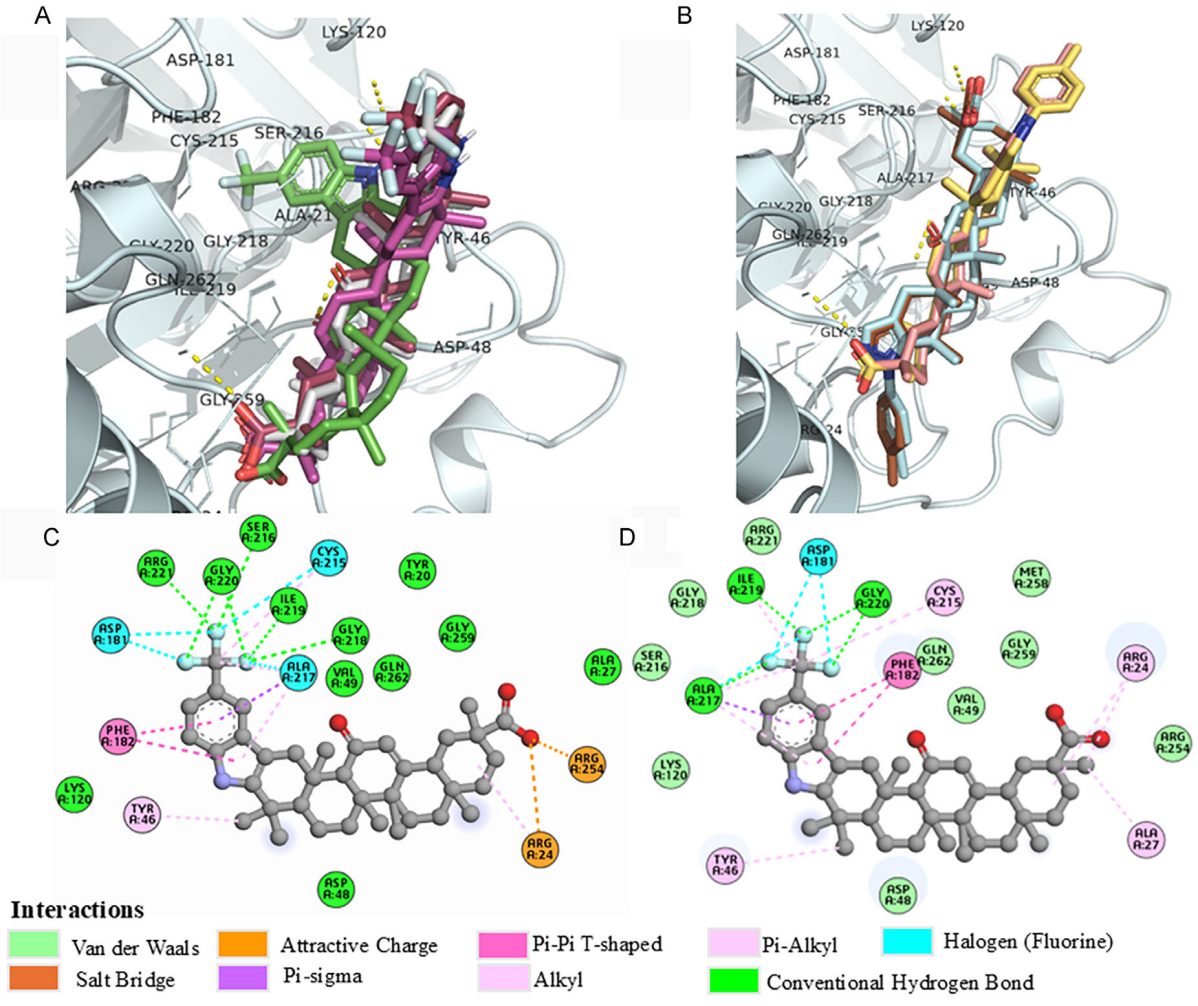
Binding poses of the indole‐GA derivatives A): **3a** (red), **3b** (pink), **3c** (gray) and **FC‐114** (green) and binding poses of the pyrazole‐GA derivates B): **5** **a** (yellow), **5b** (cyan), **5c** (light pink) and **FC‐122** (brown) docked into the allosteric binding site of PTP1B (cyan) (PDB ID: 1C83). The 2D diagram of docked FC‐114 C) and **3c** D) in PTP1B_1–298_ shows interactions with the residues at the allosteric binding site.

For blind docking, both AD and the CB‐Dock2 server identified two preferred binding sites **Figure** **4**. These sites were selected based on their scoring function and the fact that most of the tested ligands were found to be bound to them. We observed that the binding sites for these triterpenoids are located in close proximity to each other and near the catalytic site of PTP1B_1–298_ (**Figure** **5**). The first binding site includes the residues Arg^24^, Ala^27^, Tyr^46^, Asp^48^, Val^49^, Lys^120^, Phe^182^, Ala^217^, Ile^219^, Arg^254^, Gly^259^, and Gln^262^. The second site consists of residues Ala^35^, Lys^36^, Lys^41^, Asn^44^, Arg^45^, Tyr^46^, Arg^47^, Ser^118^, Leu^119^, and Lys^120^.

Given this spatial proximity, site‐directed docking simulations were performed on the noncatalytic binding site of PTP1B_1–298_, consisting of the residues Tyr^20^, Arg^24^, Arg^254^, and Gly^259^.^[^ ^35^ ^]^ All GA derivatives (**3a**, **3b**, **3c**, **5a**, **5b**, **5c**, **FC‐114**, and **FC‐122**) showed better scores and binding energies compared with the starting materials 18α‐GA and 18β‐GA across all three software programs (**Table** **3**). Furthermore, all compounds, except for 18β‐GA, form hydrogen bonds with Arg^24^ or Arg^254^ through their carboxylate group. Additionally, there were hydrophobic interactions between the GA skeleton and the residues Tyr^46^, Val^49^, Ala^217^, and Met^258^ (Figure 5A,B). Notably, only the reference compounds (**FC‐114** and **FC‐122**), which are β‐epimers of 18β‐GA having an 11‐carbonyl group, exhibited attractive interactions with Arg^24^, Arg^254^, or Lys^120^. **FC‐114** and **3c** were unique because they also displayed halogen interactions with Asp^181^, Cys^215^, and Ala^217^ (Figure 5C,D). We hypothesized that the interactions between **FC‐114** and **FC‐122**, particularly their C‐30 carboxylate group with Arg^24^, Arg^254^, or Lys^120^ within PTP1B_1–298_, may be further enhanced by the *cis* conformation between rings D and E of the 18β‐GA skeleton.

**Table 3:** Results of molecular docking simulations using the short form of PTP1B_1–298_ (PDB ID: 1C83).

PTP1B_1–298_ (PDB ID: 1C83)					Docking interactions in the binding site			
Ligand	kcal mol^−1^ a)	kcal mol^−1^ b)	GSc)	CSd)	Attractive Charge	H‐bond	Hydrophobic	Halogen
**3a**	−8.9	−8.0	41.2	34.7	–	Arg^24^, Arg^254^, Gly^259^, Gln^262^	Tyr^46^, Val^49^, Ile^219^	–
**3b**	−8.4	−7.8	46.4	41.5	–	Arg^24^, Arg^254^	Ile^219^, Met^258^	
**3c**	−9.1	−7.9	41.2	44.8	–	Arg^24^, Arg^254^, Gln^262^	Arg^24^, Tyr^46^	
**5a**	−9.1	−6.9	30.1	23.1	–	Arg^24^, Arg^254^	Arg^24^, Tyr^46^, Met^258^	
**5b**	−8.6	−6.8	45.2	26.5	–	Arg^254^	Arg^24^, Ala^27^, Tyr^46^, Val^49^, Phe^182,^ Ala^217,^ Met^258^	
**5c**	−9.4	−7.4	31.5	13.2	–	Arg^24^, Arg^254^	Arg^24^, Tyr^46^, Val^49^	
**FC‐114**	−9.2	−9.2	51.7	53.2	Arg^24^ Arg^254^	Ser^216^, Gly^218^,Ile^219^, Gly^220^, Arg^221^	Tyr^46^,Phe^182,^ Ala^217^	Asp^181,^ Cys^215,^ Ala^217^
**FC‐122**	−8.9	−7.2	46.8	1.8	Arg^254^ Lys^120^	Arg^254^	Arg^24^, Tyr^46^, Val^49^, Phe^182^, Ala^217^, Ile^219,^ Met^258^	–
**18** **α** **‐GA**	−8.6	−6.0	33.8	13.8	–	Arg^24^, Arg^254^, Gln^262^	Tyr^46^, Val^49^	
**18** **β** **‐** **GA**	−7.4	−6.0	39.6	30.7	–	Arg^24^, Ser^28^, Gln^262^	Tyr^46^, Val^49^, Ala^217^, Met^258^	

a) Binding energy values retrieved from Autodock;

b) Binding energy values retrieved from Autodock Vina;

c) Goldscore score values retrieved from GOLD;

d) CHEMPLP values retrieved from GOLD.

On the other hand, we designed a molecular docking model for uncompetitive inhibition based on the results from the kinetic enzyme assay. PTP1B in its extended form (PTP1B_1–400_) retrieved from AlphaFold (https://alphafold.ebi.ac.uk/) was initially modeled, and then the substrate *p*‐nitrophenyl phosphate (pNPP) was docked. Afterward, it was submitted to molecular dynamics simulations (MDS) using the AMBER 11 force field. We checked the quality of this complex PTP1B_1–400_‐pNPP using the Molprobity^[^ ^42^ ^]^ server (http://molprobity.biochem.duke.edu/) giving a Molprobity score = 1.08 and a Rama distribution Z‐score = −0.23 ± 0.38, whereas the structure assessment tool QMEAN^[^ ^43^ ^]^ of SWISS‐MODEL server (https://swissmodel.expasy.org/assess) gave a QMEAN Z‐score = −0.32 (see Table S3 and Figure S53, Supporting Information) indicating a good quality of the homology model since the molprobity score is a single indicator that reflects the crystallographic resolution at which such a quality would be expected. A low value indicates better model quality, whereas the Rama distribution Z‐score between −2 and 2 indicates standard protein backbone geometry.^[^ ^44^ ^]^

**Figure 6:**
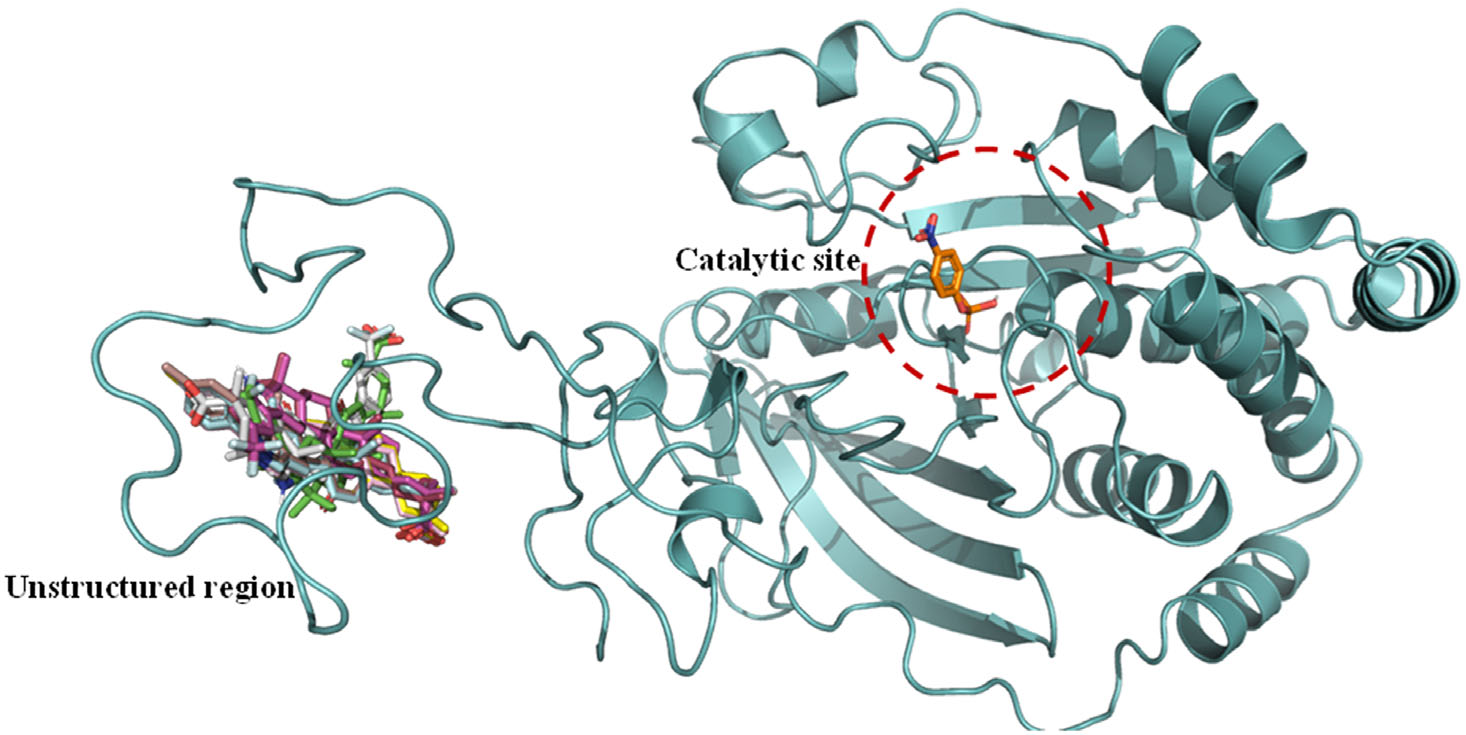
Binding mode of the indole‐GA derivatives: **3a** (red), **3b** (pink), **3c** (gray), **FC‐114** (green), and binding mode of the pyrazole‐GA derivates  **5** **a** (yellow), **5b** (cyan), **5c** (light pink), and **FC‐122** (brown) of blind docking of PTP1B_1–400._ The catalytic site of PTP1B_1–400_ is shown with the substrate pNPP (orange).

Then, we conducted a blind molecular docking using AD and VINA for each of the GA derivatives (**3a**, **3b**, **3c**, **5a**, **5b**, **5c**, **FC‐114**, and **FC‐122**), as well as for the starting materials (18α‐GA and 18β‐GA). Interestingly, the results indicated that all docked compounds exhibited better binding energies and scores in the unstructured region of PTP1B_1–400,_ specifically between amino acid residues 300–400. Following this, site‐specific molecular docking was performed within the unstructured region of PTP1B_1–400_ (**Figure** **6**). The findings revealed that all GA derivatives had improved binding energies and scores compared with the starting materials and even surpassed those observed when docked at the noncatalytic binding site of PTP1B_1–298_ (PDB ID 1C83).

**Figure 7:**
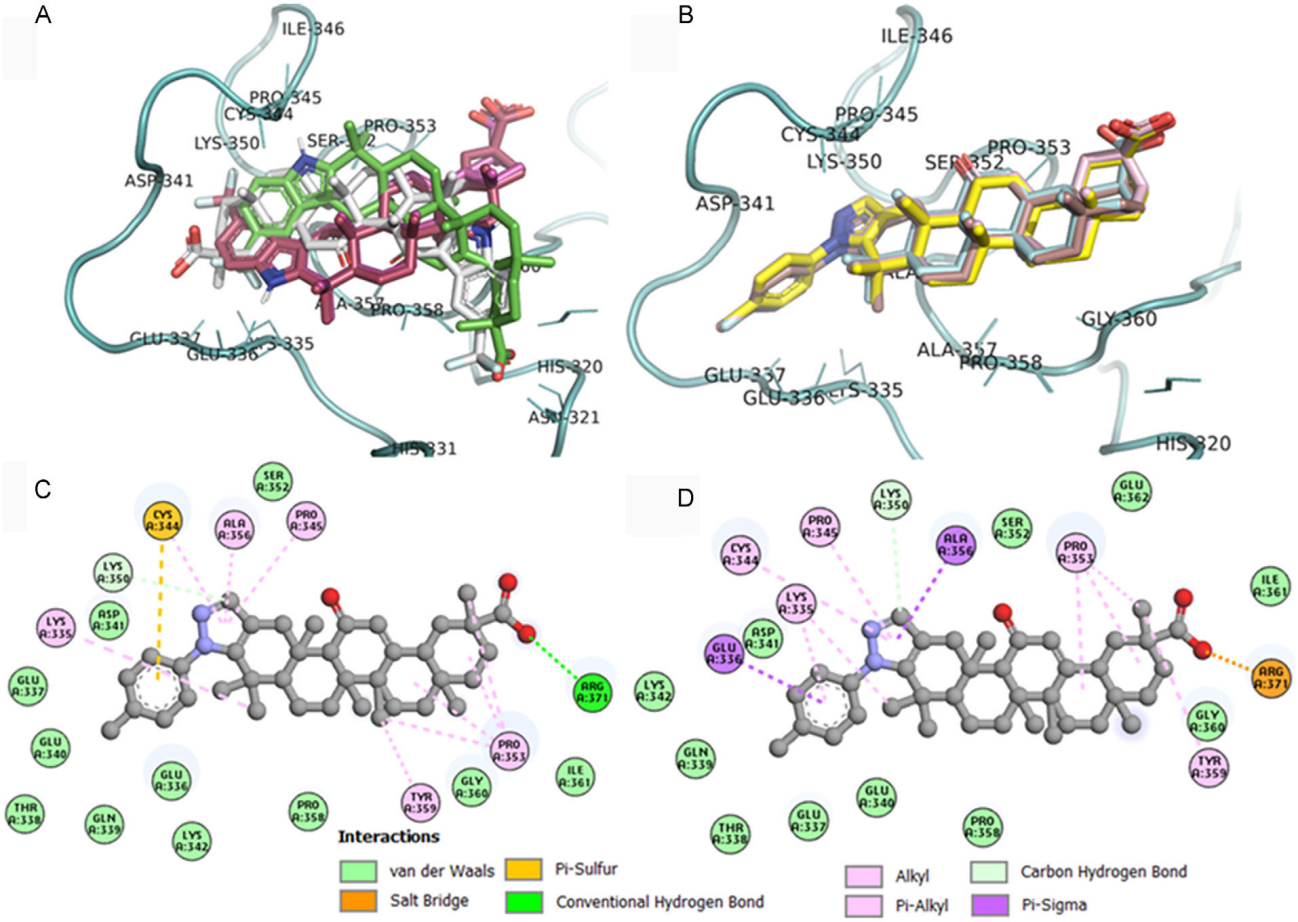
Binding poses of the indole‐GA derivatives A) **3a** (red), **3b** (pink), **3c** (gray), **FC‐114** (green) and binding poses of the pyrazolic‐GA derivatives B) 5**a** (yellow), **5b** (cyan), **5c** (light pink) and **FC‐122** (brown) docked into the unstructured region of PTP1B_1–400_. 2D diagram of docked **5c** C) and FC‐122 D) in PTP1B_1–400_‐pNPP complex, showing interactions into the unstructured region of PTP1B_1–400_.

Our analysis across all three software programs (**Table** **4**) found that all docked compounds acted as hydrogen bond acceptors through their carboxylate groups, interacting with Arg^371^, Glu^336^, Glu^337^, or Asn^321^. Only **FC‐122** formed a salt bridge with Arg^371^ via its carboxylate group. Furthermore, all docked compounds exhibited hydrophobic interactions with the side chain of residues Lys^335^, Glu^336^, Pro^345^, Pro^353^, Ala^356^, Pro^358^, Tyr^359^, and Cys^344^ through its GA skeleton (**Figure** **7**A,B). The indole‐GA derivatives (**3a**, **3b, 3c**, and **FC‐114**) displayed halogen interactions between their ‐CF_3_ group and Lys^350^ or Lys^335^ and Gln^332^ or Pro^358^ through unconventional interactions of C‐F···C=O. In contrast, the pyrazole‐GA derivatives (**5a**, **5b**, and **5c** (Figure 7C)) displayed *π*‐sulfur interactions with Cys^344^. Importantly, we observed that when the 11‐ketone group is present (**3c**, **FC‐114**, and **FC‐122** (Figure 7D), the carboxylate group at C‐30 forms more hydrogen bond interactions within the allosteric site of PTP1B compared with compounds where the 11‐carbonyl group is absent (**3a**, **3b**, **5a**, and **5b**). This suggests that this group is crucial for maintaining inhibitory activity against PTP1B, as observed in the in vitro inhibitory activity studies against *h*PTP1B_1–400_.

**Table 4:** Results of molecular docking simulations using the long form of PTP1B_1–400_.

PTP1B_1–400_					Docking interactions with the binding site				
Ligand	kcal mol^−1^ a)	kcal mol^−1^ b)	GSc)	CSd)	H‐bond	Hydrophobic	Halogen	*π*‐sulfur	*π*‐anion
**3a**	−9.2	−8.8	48.1	31.8	Arg^371^	Lys^335^, Pro^345^, Pro^353^, Pro^358^, Tyr^359^, Ala^356^	Lys^350^	–	–
**3b**	−9.5	−8.7	51.1	36.2	Arg^371^	Lys^335^, Pro^345^, Pro^353^, Pro^358^, Tyr^359^	Lys^350^		
**3c**	−9.7	−9	47.4	29.3	Glu^337^, Gln^339^, Asn^321^	Lys^335^, Cys^344^, Pro^345^, Pro^353^	Gln^332^Pro^358^		
**5a**	−10.6	−8.6	46.9	9.9	Arg^371^	Lys^335^, Cys^344^, Pro^345^, Pro^353,^ Ala^356^	–	Cys^344^	
**5b**	−11.2	−7.7	41.4	5.7	Arg^371^	Lys^335^, Pro^345^, Pro^353^, Ala^356,^ Pro^358^, Tyr^359^		Cys^344^	
**5c**	−10.5	−8.8	53.5	31.6	Arg^371^	Lys^335^, Pro^345^, Pro^353^, Ala^356,^ Pro^358^, Tyr^359^, Cys^344^		Cys^344^	
**FC‐114**	−9.2	−8.9	51.4	37.4	His^320^, Asn^321^, Glu^336^	Lys^335^, Pro^345^, Pro^353^, Ala^356,^ Pro^358^, Cys^344^	Lys^335^	–	His^331^
**FC‐122**	−10.5	−8.8	52.4	6.6	Arg^371^(salt‐bridge)	Lys^335^, Glu336, Pro^345^, Pro^353^, Ala^356,^ Pro^358^, Tyr^359,^ Cys^344^	–		–
**18** **α** **‐GA**	−9.5	−6.6	35.0	20.1	Gly^360^	Lys^335^, Pro^345^, Pro^353^, Ala^356,^ Pro^358^, Tyr^359,^ Cys^344^			
**18** **β** **‐GA**	−8.9	−6.6	38.9	30.8	Glu^336^, Glu^337^	Lys^335^, Cys^344^, Lys^350^, Pro^353^, Ala^356^			

a) Binding energy values retrieved from Autodock;

b) Binding energy values retrieved from Autodock Vina;

c) Goldscore score values retrieved from GOLD;

d) CHEMPLP values retrieved from GOLD.

To understand the selectivity of GA (18α‐GA and 18β‐GA) and its derivatives (**3a**‐**3c**, **5a**‐**5c**, **FC‐114**, and **FC‐122**) for PTP1B over TCPTP, a blind docking simulation was performed using the modeled TCPTP_1–415._ We checked the quality of the modeled TCPTP_1–415_ using the MolProbity^[^ ^42^ ^]^ server (http://molprobity.biochem.duke.edu/) giving a Molprobity score = 0.63 and a Rama distribution Z‐score = 0.98 ± 0.38, whereas the structure assessment tool QMEAN^[^ ^43^ ^]^ of SWISS‐MODEL server (https://swissmodel.expasy.org/assess) gave a QMEAN *Z*‐score = 0.16 (see Table S4 and Figure S54, Supporting Information), indicating a good quality of the homology model.^[^ ^44^ ^]^

The results of this study indicated that all docked ligands preferentially bind to a site that includes the amino acids Trp^18^, Gln^19^, Tyr^22^, Arg^26^, Pro^181^, Phe^183^, Gln^260^, Pro^262^, Val^365^, Met^368^, Lys^369^, and Leu^372^. Further docking studies focused on this region revealed that all ligands exhibited affinity for the enzyme across three different programs (AD, VINA, and GOLD) (**Table** **5**), with binding energies ranging from −7.1 to −10.5 kcal mol^−^^1^ and scores of 10.1 and 60.2. Both 18α‐GA and 18β‐GA showed weaker affinities compared to their derivatives. It was also observed that the carboxylate group of compounds **3a**, **3c**, **5a**, **5b**, **5c**, and 18β‐GA formed hydrogen bonds with Ala^218^, Ile^220^, or Gly^221^. Additionally, compounds having the 11‐carbonyl (**3c**, **5c**, 18α‐GA, 18β‐GA, and **FC‐122**) performed hydrogen bonds with Lys^118^ or Lys^122^. Interestingly, only the compounds that included fluorine in their structure (**3a**, **3b**, **3c**, and **FC‐114**) formed hydrogen bonds through fluorine with residues Lys^364^, Gln^19^, Ala^218^, Gly^219^, Ser^217^, or Arg^222^, as well as engaged in unconventional halogen interactions with Lys^118^, Asp^182^, or Cys^216^. Furthermore, all compounds (**3a**, **3b**, **3c**, **5a**, **5b**, **5c**, **FC‐114**, **FC‐122**, 18α‐GA, and 18β‐GA) demonstrated hydrophobic interactions with Tyr^48^, Phe^183^, Lys^118^, Ile^220^, and Arg^357^ through the GA skeleton. Recently, Singh et al. in 2022^[^ ^45^ ^]^ reported that TCPTP consists of a catalytic domain (residues 1–302) and a C‐terminal tail (residues 303–387). Unlike PTP1B, the C‐terminal tail of TCPTP interacts with the catalytic domain through residues 344–385, leading to self‐inhibition (or autoinhibition) of the enzyme, which reduces its activity. They also indicated that the activation of TCPTP and enhanced substrate dephosphorylation could be achieved by inhibiting the C‐terminal tail of TCPTP (residues 344–385) using peptides derived from integrin α−1.^[^ ^45^ ^]^

**Table 5:** Results of molecular docking with TCPTP_1–415_.

Ligand	kcal mol^−1^ a)	kcal mol^−1^ b)	GSc)	CSd)	Ligand interactions with the binding site		
					H‐bond	Hydrophobic	Halogen
**3a**	−9.5	−9.3	60.2	47.1	Ala^218^, Ile^220^, Gly^221,^ Lys^364^	Val^51^, Phe^183^, Arg^357^, Lys^358^	Lys^118^
**3b**	−9.7	−9.4	49.7	46.6	Gln^19^, Lys^122^	Tyr^48^, Phe^183^, Ala^218^, Ile^220^	Lys^350^
**3c**	−9.3	−9.8	45.3	41.7	Lys^118^, Ser^217^, Ala^218^, Gly^219^, Arg^222^, Lys^364^	Phe^183^, Ile^220^, Arg^357^	Asp^182^, Cys^216^
**5a**	−9.8	−8.6	60.2	37.6	Ala^218^, Ile^220^, Gly^221^, Cys^216^	Tyr^48^, Lys^118^, Phe^183^, Arg^357^	–
**5b**	−9.7	−8.9	44.4	10.1	Ala^218^, Gly^219^, Ile^220^, Gly^221^	Tyr^48^, Lys^118^, Phe^183^, Ile^220^, Arg^357^	
**5c**	−10.5	−9.2	55.8	24.3	Lys^118^, Lys^122^, Cys^216^, Ala^218^, Ile^220^, Gly^221^	Tr^48^, Phe^183^, Arg^357^	
**FC‐114**	−9.4	−8.9	52.6	41.8	Gln^19^, (Lys^118^, Lys^122^; attractive charge)	Tyr^48^, Ala^218^, Pro^262^	Lys^335^
**FC‐122**	−9.6	−8.8	41.5	15.3	Lys^118^, Lys^364^	Tyr^48^, Lys^118^, Ala^218^, Arg^357^, Thr^360^	–
**18** **α** **‐GA**	−9.4	−7.1	35.6	28.3	Lys^122^, Asp^182^, Ser^217^, Arg^354^	Tyr^48^, Arg^49^, Phe^183^, Ala^218^, Ile^220^	
**18** **β** **‐GA**	−9.1	−7.4	50.2	25.7	Lys^122^, Ala^218^, Ile^220^, Gly^221^, Lys^364^	Lys^118^, Phe^183^, Ile^220^	

a) Binding energy values retrieved from Autodock;

b) Binding energy values retrieved from Autodock Vina;

c) Goldscore score values retrieved from GOLD;

d) CHEMPLP values retrieved from GOLD.

The results from our molecular docking simulations indicate that GA and its derivatives can bind to both the catalytic domain and the C‐terminal tail of TCPTP, similar to the behavior observed with integrins. Therefore, GA derivatives may activate TCPTP by displacing the autoregulatory C‐terminal tail, thus making the catalytic site available for the pNPP substrate. Our in vitro assays supported this hypothesis, which showed that these compounds did not inhibit TCPTP even at concentrations up to 100 μM.

### 2.7 Molecular Dynamics Simulations (MDS)

To better understand the impact of the inversion of 18β‐H to 18α‐H in the derivatives FC‐114 and FC‐122 on the stability of the complexes formed with PTP1B, we conducted molecular MDS using the YASARA Structure software^[^ ^46^ ^,^ ^47^ ^]^ and the AMBER11 (also known as AMBER ff99sb*‐ILDN) force field.^[^ ^48^ ^]^ Four studies were carried out for the protein‐ligand complexes: PTP1B_1–400_‐pNPP‐**FC‐114**, PTP1B_1–400_‐pNPP‐**FC‐122**, PTP1B_1–400_‐pNPP‐**3c**, and PTP1B_1–400_‐pNPP‐**5c**, which resulted from docking simulations at the C‐terminal unstructured region of PTP1B. In the first study, we evaluated root mean square deviation (RMSD) fluctuations of the C*α* carbons to assess the stability of each protein–ligand complex. In the second study, we evaluated the mean square fluctuation (RMSF) to determine the stability of amino acids. Additionally, we calculated the binding energy of each ligand within the C‐terminal unstructured region of PTP1B, as well as the binding energy of pNPP when each ligand interacts within the C‐terminal unstructured region of PTP1B, throughout 200 nanoseconds of MDS.

#### 2.7.1 Root Mean Square Deviation (RMSD) Analysis

**Figure 8:**
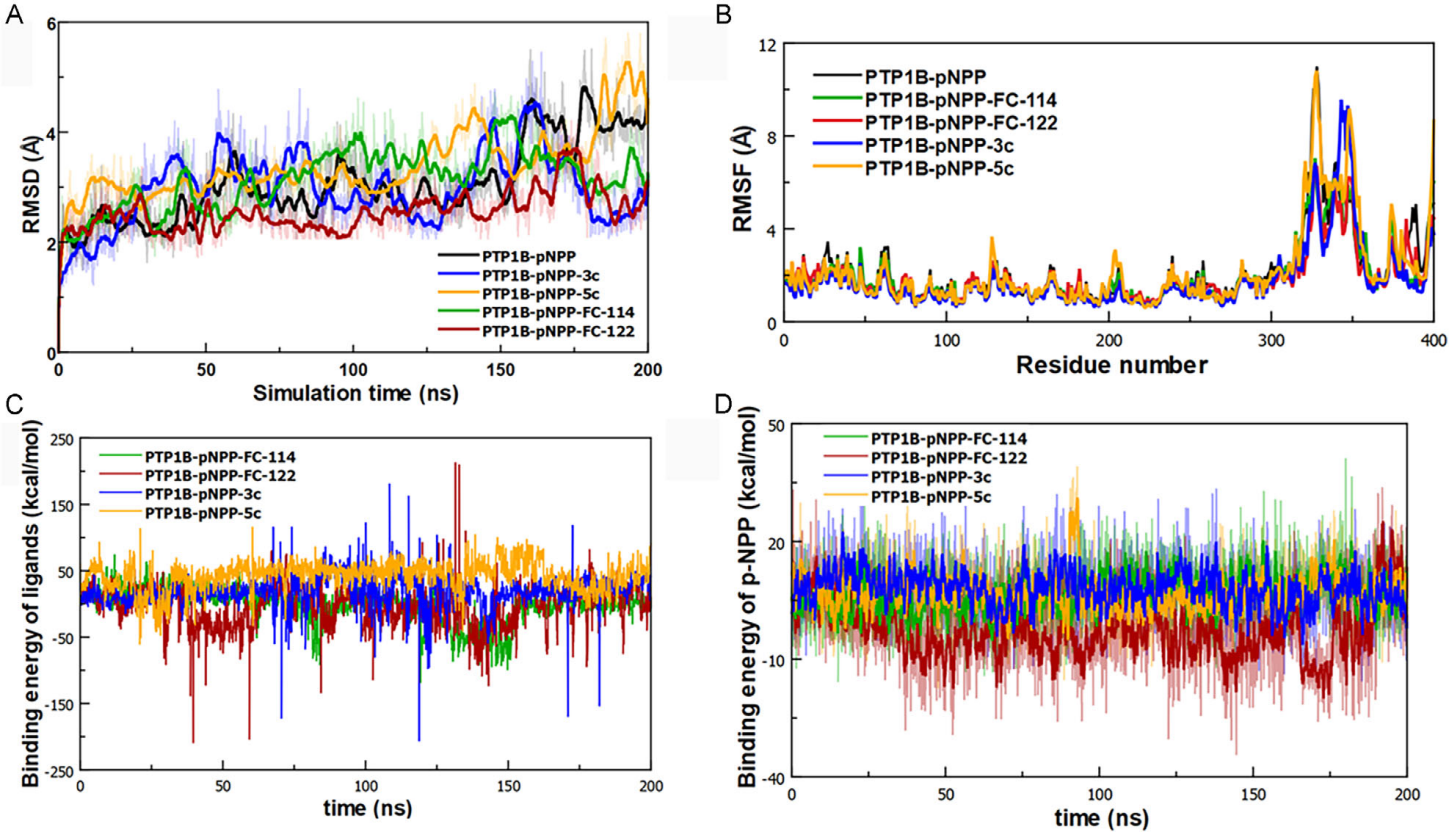
A) Standard RMSD of PTP1B_1–400_‐pNPP, PTP1B_1–400_–pNPP‐**FC‐114**, PTP1B_1–400_–pNPP‐**FC‐122**, PTP1B_1–400_‐pNPP‐**3c,** and PTP1B_1–400_–pNPP‐**5c** systems. B) Fluctuation of amino acid residues due to ligand binding measured by root–mean‐square‐fluctuation (RMSF) values. C) Binding energy of ligands: **FC‐114**, **FC‐122**, **3c** and **5c** within the PTP1B_1–400_–pNPP‐**FC‐114**, PTP1B_1–400_–pNPP‐**FC‐122**, PTP1B_1–400_‐pNPP‐**3c**, and PTP1B_1–400_–pNPP‐**5c** systems. D) Binding energy of pNPP within the PTP1B_1–400_–pNPP‐**FC‐114**, PTP1B_1–400_–pNPP‐**FC‐122**, PTP1B_1–400_‐pNPP‐**3c** and PTP1B_1–400_–pNPP‐**5c** systems.

In this study, we performed an RMSD analysis on the PTP1B_1–400_‐pNPP complex as well as the PTP1B_1–400_‐pNPP‐**FC‐114**, PTP1B_1–400_‐pNPP‐**FC‐122**, PTP1B_1–400_‐pNPP‐**3c**, and PTP1B_1–400_‐pNPP‐**5c** complexes over a 200‐nanosecond simulation period using AMBER 11 (also known as AMBER ff99sb*‐ILDN) force field.^[^ ^48^ ^]^ The results of this analysis serve as an indicator of the overall fluctuation of the protein's C*α* carbons. Thus, the lower the value of RMSD, the less fluctuation of the C*α* carbons, indicating greater stability for the protein's main chain atoms. Consequently, lower RMSD values suggest that the ligand stabilizes the PTP1B_1–400_‐pNPP–ligand complex. As shown in **Figure** **8**A, the PTP1B_1–400_‐pNPP system tends to stabilize after 25 nanoseconds with an average RMSD value = 3.14 Å from 0 to 200 nanoseconds. Similarly, the PTP1B_1–400_‐pNPP‐**FC‐114** complex stabilizes with an RMSD of 3.14 Å. In contrast, the PTP1B_1–400_‐pNPP‐**5c** complex shows more significant fluctuations, with a higher RMSD value of 3.39 Å.

A slightly lower mean RMSD value of 2.97 Å was observed in the PTP1B_1–400_‐pNPP‐**3c** complex, indicating that it can stabilize the PTP1B_1–400_‐pNPP–ligand complex. Additionally, it is evident that the PTP1B_1–400_‐pNPP‐**FC‐122** complex exhibits the lowest overall fluctuation among the five complexes, with a mean RMSD value of 2.56 Å. This suggests that the binding of **F** **C‐122** at the C‐terminal unstructured region of PTP1B has the most significant effect on stabilizing the overall PTP1B_1–400_‐pNPP–ligand complex.

#### 2.7.2 Root Mean Square Fluctuation (RMSF) Analysis

The chart (Figure 8B) indicates that the PTP1B_1–400_‐pNPP‐**FC‐114** and PTP1B_1–400_‐pNPP‐**FC‐122** complexes showed lower fluctuations in the amino acid sequence within the C‐terminal unstructured region (300–400 residues) compared with the PTP1B_1–400_‐pNPP system. In contrast, increased values of RMSF were noted in this same region for the PTP1B‐pNPP‐**3c** and PTP1B_1–400_‐pNPP‐**5c** complexes, particularly at the residues Arg^325^ and Lys^350^, suggesting a conformational change in the C‐terminal unstructured region (300–400 residues). Furthermore, the analysis of the two ligand‐contact graphs revealed that the ligands **FC‐114**, **FC‐122**, **3c**, and **5c** maintained hydrophobic interactions with several residues, including Gln^332^, Lys^335,^ Glu^336^, Glu^337^, Cys^344^, Pro^345^, Pro^353^, Ala^356^, and Pro^358^. However, hydrogen and halogen bond interactions were no longer observed during the 200 ns of molecular dynamics simulation (see Figure S55–S58, Supporting Information).

#### 2.7.3 Binding Energy Analysis

To quantitatively address the affinity of the four inhibitors, we performed a binding energy analysis as a function of the simulation time for 200 ns using the MM‐PBSA method (Figure 8C). The binding energy was obtained by calculating the energy of the ligand–protein complex system (i.e., the bound state) and subtracting the energy at an infinite distance between the ligand and the rest of the protein system (i.e., the unbound state) at intervals of 100 ps. More negative values indicate better binding in the context of the AMBER11 force field.

Among the four simulated complexes, PTP1B_1–400_‐pNPP‐**3c** and PTP1B_1–400_‐pNPP‐**5c** had average binding energy values of 19.33 and 42.53 kcal mol^−^^1^, respectively. Contrary, PTP1B_1–400_‐pNPP‐**FC‐114** and PTP1B_1–400_‐pNPP‐**FC‐122** exhibited more negative average binding energy values (−1.45 and −6.58 kcal mol^−^^1^, respectively), suggesting stronger binding in the C‐terminal unstructured region of PTP1B_1–400_‐pNPP complex. The compound **FC‐122** displayed the best binding energy at this region of the PTP1B_1–400_‐pNPP complex. Conversely, we addressed the affinity of the pNPP substrate when each ligand interacts with the C‐terminal unstructured region of PTP1B during the 200 nanoseconds of MD simulation (Figure 8D). Our analysis indicated that when **FC‐122** binds to the C‐terminal unstructured region of PTP1B, the pNPP shows a better average binding energy to the catalytic site (−2.74 kcal mol^−^^1^) compared with when it is bound to the other ligands: **3c**, **5c**, and **FC‐114**, which had average binding energy values of 8.05, 6.64, and 6.64 kcal mol^−^^1^, respectively. This suggests a more favorable interaction between the pNPP and the catalytic site of PTP1B, potentially preventing the binding of other molecules in the catalytic site.

Results of the MDS reveal that the inversion of the stereochemistry of 18β‐H to 18α‐H has a significant impact on the structural properties because the 18β‐H complexes (PTP1B_1–400_‐pNPP‐**FC‐122** and PTP1B_1–400_‐pNPP‐**FC‐114**) showed better structural stability evidenced by a lower RMSD and a lower RMSF in amino acids 300–400 corresponding to the region unstructured PTP1B compared with the complexes of the 18*α*‐H derivatives (PTP1B_1–400_‐pNPP‐**3c** and PTP1B_1–400_‐pNPP‐**5c**). Furthermore, the 18β‐H conformation showed a more favorable interaction with PTP1B because the binding energy of the complex, as well as the binding energy of the p‐NPP substrate, was better in the 18β‐H complexes (PTP1B_1–400_‐pNPP‐**FC‐122** and PTP1B_1–400_‐pNPP‐**FC‐114**) than that of 18α‐H complexes (PTP1B_1–400_‐pNPP‐**3c** and PTP1B_1–400_‐pNPP‐**5c**).

### 2.8 Biological Assessment of GA Derivatives against Insulin‐Resistant HepG2 Cells

**Figure 9:**
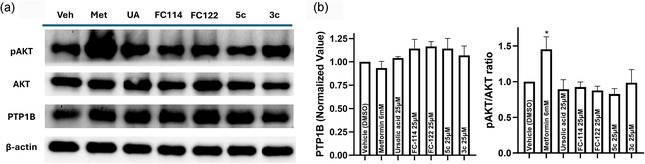
Effects of metformin, ursolic acid, **FC‐114**, **FC**‐**122**, **3c**, and **5c** on the levels of PTP1B and pAKT in an insulin resistance model in HepG2 cells. a) Western blotting representative analysis (*n* = 3) of PTP1B and pAKT levels of HepG2 cells treated with the compounds at 24 h of treatment. β‐actin was employed as the loading control. b) Densitometric analysis of western blot images employing ImageJ analysis software. Bar graphs represent the mean ± SEM of normalized values. The control levels were set at 1. T‐test significance *p < 0.05, when compared with control (DMSO). Statistical analyses were carried out using GraphPad Prism 8.0.

To evaluate the potential effects of the most potent PTP1B inhibitors identified in this study, HepG2 cells were exposed to different concentrations of **FC‐114**, **FC‐122**, **3c**, and **5c**. The findings revealed that these triterpenoids derivatives did not compromise HepG2 cell viability at 24 h (see Figure S59, Supporting Information), demonstrating reduced cytotoxic activity. Subsequently, we evaluated the same compounds in a HepG2 insulin‐resistant model. Treatments that enhance insulin signaling, such as metformin, have been reported to decrease PTP1B levels in in vitro studies, although the underlying mechanisms are not fully understood.^[^ ^49^, ^50^ ^–^ ^51^ ^]^ In our study, neither metformin, ursolic acid, nor GA derivatives significantly reduced PTP1B levels. However, we observed a significant increase in AKT phosphorylation (pAKT) levels with metformin, whereas ursolic acid did not demonstrate a notable change in pAKT levels. Among the synthesized derivatives, only compound **3c** exhibited a slight increase in pAKT levels (**Figure** **9**).

## 3 Conclusion

Six new derivatives of glycyrrhetinic acid were synthesized from glycyrrhizin. The study investigated the impact of inverting the 18β‐H proton to 18α‐H, as well as the absence of the 11‐carbonyl group at position C11 in the compounds **FC‐114** and **FC‐122**, on the inhibitory activity of the enzymes PTP1B and TCPTP. Assays on the PTP1B enzyme demonstrated that the inversion of the 18β‐H group and the absence of the 11‐ketone negatively affected the inhibitory activity. Specifically, these modifications reduced the potency against PTP1B by up to four times. Notably, the synthesized compounds exhibited uncompetitive inhibition, with Ki values ranging from 1.16 to 0.017 μM. Among the two modifications, the absence of the 11‐ketone group had the most significant negative impact on inhibitory activity.

Molecular docking and molecular dynamics simulations suggested that these modifications reduce inhibitory activity against PTP1B because the inversion of 18β‐H pushes the 30‐COOH group away, disrupting key interactions at the unstructured C‐terminal site of PTP1B_1–400_. Meanwhile, the absence of the 11‐carbonyl group positioned the compounds unfavorably, hindering their ability to form important interactions in the same zone of PTP1B_1–400_.

Moreover, both the reference compounds **FC‐114** and **FC‐122**, along with the synthesized derivatives, showed selectivity for PTP1B over TCPTP, as they did not inhibit TCPTP at concentrations of up to 100 μM. It was observed that only compound **3c** slightly increased pAKT levels in an assay using insulin‐resistant HepG2 cells, though this increase was not statistically significant. These findings indicate that while 18α‐glycyrrhetinic acid is a more potent inhibitor of PTP1B than 18β‐glycyrrhetinic acid, the effect reverses when both triterpenes are modified with fused heterocycles such as indole or *N*‐phenylpyrazole in the A ring of their structure.

## 4 Experimental Section

4.1

4.1.1

##### Chemicals and Reagents

All reagents and starting materials were obtained from Sigma–Aldrich (Toluca, MEX, Mexico, and St. Louis, MO, USA). Reactions were monitored by thin‐layer chromatography on 0.2 mm silica gel‐coated 60 F254 plates (Sigma–Aldrich) and visualized under UV light. Melting points were determined using a Fischer–Johns melting point apparatus without correction. Both ^1^H and ^13^C NMR spectra were recorded on Agilent DD2 (Agilent, Santa Clara, CA, USA) and Bruker Ascend spectrometers (Bruker, Billerica, MA, USA) operating at 600 and 400 MHz for ^1^H, and at 151 MHz for ^13^C, respectively. Chemical shifts are reported in parts per million (ppm) relative to tetramethylsilane (Me_4_Si = 0); coupling constants (*J* values) are expressed in Hertz (Hz). Multiple patterns are indicated as follows: s, singlet; d, doublet; q, quartet; dd, doublet of doublets; t, triplet; m, multiplet; bs, broad singlet. High‐resolution mass spectra (HRMS) were obtained using a micrOTOF‐ESI‐TOF‐MS mass spectrometer by direct infusion and in a positive mode using nitrogen (4 mL min^−^^1^) as nebulizer gas, spray voltage (4.5 kV) at 150 °C, within a mass range of *m*/*z* 50−3000. The results are expressed as *m*/*z*. All data spectra are reported in the Supporting Information. According to IUPAC rules, compounds were named using the automatic generator tool implemented in ChemDraw Professional 22.0.0 software (PerkinElmer, Waltham, MA, USA).

##### Synthesis of 18α‐Glycyrrhetinic Acid

This product was prepared using the method described by *Jitrangsri* et al. with some modifications.^[^ ^28^ ^]^ A solution of ammonium salt of 18β‐glycyrrhizin (5 g, 5.95 mmol) was refluxed with KOH (6.7 g, 119.4 mmol) in water (30 mL) for 12 h to yield the epimerized glycyrrhizin (5.1 g, 5.95 mmol). This crude compound (5.1 g, 5.95 mmol) was esterified with dimethyl sulfate (2.8 mL, 29.6 mmol) and potassium carbonate (4.0 g, 29.6 mmol) in dimethyl sulfoxide (18 mL) to obtain a mixture of trimethyl esters (18β and 18α‐glycyrrhizins), which were purified by column chromatography using dichloromethane: methanol (95:5). After purification, the pure 18α‐methylglycyrrhizate was obtained by recrystallization in methanol. A solution of 18*α*‐methylglycyrrhizate (1.0 g) in ethanol (6.5 mL) and 10% KOH (6.5 mL) was refluxed for 3 h to give 18α‐glycyrrhizic acid.^[^ ^28^ ^]^ Finally, 18α‐glycyrrhizic acid was hydrolyzed in refluxing HCl solution (12.5 mL H_2_O, 2.6 mL conc. HCl) overnight to yield 18α‐glycyrrhetinic acid^[^ ^52^ ^]^ as a brown solid, which after crystallization with a mixture of methanol and water yielded a white powder (20%) with a melting point >300 °C Ref [330–335 °C].^[^ ^53^ ^]^

##### Methyl‐18*α*‐3*β*‐hydroxy‐olean‐12‐en‐30‐oate

^1^HNMR (600 MHz, CDCl_3_) *δ*_H_: 5.56 (s, 1H, H‐12), 3.68 (s, 3H, OCH_3_), 3.22 (dd, *J* = 11.5, 4.7 Hz, 2H, H‐3 and 3‐OH), 2.25 (s, 1H, H‐9), 2.22 (d, *J* = 11.7 Hz, 1H, H‐18), 2.02—1.85 (m, 2H, H‐16 and H‐2), 1.72–1.52 (m, 6H, H‐1, 7‐CH_2_, H‐15 and 21‐CH_2_), 1.52–1.36 (m, 8H, H‐1, H‐2, H‐15, H‐16, 19‐CH_2_, 22‐CH_2_), 1.33 (s, 3H, 27‐CH_3_), 1.21 (s, 3H, 29‐CH_3_), 1.18 (s, 3H, 25‐CH_3_), 1.12 (s, 3H, 26‐CH_3_), 0.99 (s, 3H, 23‐CH_3_), 0.92 (m, 1H, H‐6), 0.80 (s, 3H, 24‐CH_3_), 0.86 (m, 1H, H‐6), 0.70 (s, 3H, 28‐CH_3_), 0.67 (s, 1H, H‐5). ^13^C NMR (151 MHz, CDCl_3_): *δ*_C_ 199.98 (C11), 178.94 (C30), 165.82 (C13), 124.26 (C12), 78.93 (C3), 60.79 (C9), 55.11 (C5), 52.09 (OCH_3_), 45.03 (C14), 43.97 (C8), 40.50 (C20), 39.24 (C4), 39.19 (C18), 37.74 (C1), 36.96 (C22), 39.24 (C10), 36.96 (C17), 36.05 (C19), 33.90 (C7), 31.90 (C21), 28.57 (C16), 28.23 (C23), 27.36 (C15), 26.80 (C2), 20.78 (C27 and C29), 18.62 (C26), 16.71 (C25), 16.13 (C24), 15.81 (C28).

##### 18α‐Glycyrrhetinic Acid

^1^H NMR (400 MHz, DMSO‐d_6_): *δ*_H_ 5.33 (s, 1H, H‐12), 4.33 (s, 1H, OH), 3.01 (d, *J* = 7.4 Hz, 1H, H‐3), 2.45 (d, *J = *13.32 Hz, 1H, H‐1), 2.28–2.26 (s, 2H, H‐18 and H‐9), 1.88 (m, 1H, H‐2), 1.75 (m, 1H, H‐16), 1.73 (m, 2H, H‐7 and H‐22), 1.56–1.43 (m, 4H, H‐6, H‐7, H‐15, and H‐19), 1.42–1.34 (m, 6H, H‐6, H‐7, H‐15, H‐16, H‐21, and H‐22), 1.32 (s, 3H, 27‐CH_3_), 1.20–1.27 (m, 3H, H‐2, H‐18, H‐19), 1.16 (s, 3H, 29‐CH_3_), 1.08 (25‐CH_3_), 1.04 (26‐CH_3_), 0.94 (m, 1H, H‐1), 0.90 (23‐CH_3_), 0.68 (24‐CH_3_), 0.67 (m, 1H, H‐5), 0.64 (s, 3H, 28‐CH_3_). ^13^C NMR (101 MHz, DMSO‐d_6_) *δ*_C_ 198.88 (C11), 179.56 (C30), 166.22 (C13), 123.02 (C12), 76.68 (C3), 60.00 (C9), 54.20 (C5), 44.73 (C14), 43.39 (C8), 41.63 (C20), 40.1 (C4), 39.49 (C18), 38.46 (C1), 36.68 (C10), 36.49 (C22), 35.30 (C17), 35.16 (C19), 33.23 (C7), 31.47 (C21), 28.40 (C16), 28.22 (C23), 26.96 (C15), 26.30 (C2), 20.66 (C29), 20.45 (C27), 18.25 (C26), 17.29 (C6), 16.44 (C25), 16.13 (C24), 15.71 (C28).

##### General Procedure for Clemmensen Reaction (Compounds 1a and 1b)

A solution of 18α‐GA or 18β‐GA (1 g, 2.1 mmol) and zinc powder (1.1 g, 8 mmol) in THF (30 mL) was cooled in an ice bath. Afterward, concentrated HCl (4.5 mL) was added dropwise over 30 min. The reaction mixture was stirred at room temperature overnight. The THF was then removed by rotary evaporation, and 30 mL of water was added to the reaction mixture. Finally, the precipitate was filtered under vacuum and washed with distilled water (2 × 20 mL). The crude product was purified by column chromatography using hexane:ethyl acetate (7:3) as eluent.

##### 18α‐11‐deoxo‐olean‐12‐en‐30‐oic Acid (1a)

Yield 88% (0.845 g); white solid; melting point: >300 °C Ref [328.1–330.6 °C].^[^ ^54^ ^]^

##### 18β‐11‐deoxo‐olean‐12‐en‐30‐oic Acid (1b)

Yield 70% (0.673 g); white solid; melting point: >300 °C Ref [324–326 °C].^[^ ^55^ ^]^

##### General Procedure for Jones Oxidation (compounds 2a and 2b)

To a solution of **1a** (0.5 g, 1.092 mmol) in THF (10 mL), 0.9 mL of Jones reagent (2.67 g CrO_3,_ 2.3 mL H_2_SO_4_, and 10 mL water) was added dropwise at 0 °C. After the addition, the reaction mixture was stirred at room temperature for 4 h. Subsequently, THF was removed by vacuum distillation, and 15 mL of distilled water was added to the reaction mixture. The precipitate was filtered under vacuum and washed with distilled water (2 × 15 mL). The crude reaction product was purified by column chromatography using a hexane:ethyl acetate (8:2) mixture as the eluent.

##### 18α‐3‐oxo‐11‐deoxy‐olean‐12‐en‐30‐oic Acid (2a)

Yield 81% (0.404 g); white solid; melting point: 290–295 °C, ^1^H NMR (600 MHz, CDCl_3_): *δ*_H_ 5.65 (s, 1H), 2.87 (m, 1H), 2.64 (m, 1H), 2.36 (s, 1H), 2.35 (m, 1H), 2.4 (m, 1H), 2.25 (d, *J* = 12.1 Hz, 1H), 2.07–1.92 (m, 2H), 1.69 (m, 2H), 1.64–1.36 (m, 11H). 1.35 (s, 3H), 1.33(s, 3H), 1.26 (s, 3H), 1.18 (s, 3H), 1.10 (s, 3H), 1.07 (s, 3H), 0.74 (s, 3H).

##### 18β‐3‐oxo‐11‐deoxy‐olean‐12‐en‐30‐oic Acid (2b)

Yield 79% (0.393 g); white solid; melting point: 285–288 °C, ^1^H NMR (600 MHz, CDCl_3_): *δ*_H_ 5.32 (s, 1H), 2.55 (m, 1H), 2.37 (m, 1H), 2.04–1.74 (m, 8H), 1.64 (m, 2H), 1.58–1.47 (m, 2H), 1.46–1.27 (m, 6H), 1.20 (s, 3H), 1.14 (s, 3H), 1.09 (s, 3H), 1.07 (s, 3H), 1.05 (s, 3H), 1.01 (s, 3H), 0.82 (s, 3H). ^13^C NMR (151 MHz, CDCl_3_) *δ*_C_ 218.20, 183.84, 144.73, 122.64, 55.47, 48.20, 47.70, 47.02, 44.29, 42.60, 41.78, 39.93, 39.41, 38.42, 36.86, 34.34, 32.26, 32.10, 31.27, 28.86, 28.32, 27.08, 26.55, 26.22, 26.02, 23.70, 21.72, 19.81, 16.74, 15.35.

##### 18α‐3‐oxo‐11‐oxo‐olean‐12‐en‐30‐oic Acid (2c)

This compound was prepared from a solution of 18α‐GA (1 g, 2.19 mmol) in 30 mL of DCM with IBX (1.7847 g, 6.37 mmol) overnight at room temperature. Subsequently, the reaction mixture was filtered over celite and washed with DCM. Then, the reaction mixture was concentrated using a rotavapor. The crude reaction product was recrystallized in methanol to yield a white solid (0.830 g, 81%) with a melting point: >300 °C Ref [310–332 °C].^[^ ^56^ ^]^

##### General Procedure for Claisen Condensation (Compounds 4a, 4b, and 4c)

A solution of **2a** (0.66 mmol), **2b** (0.66 mmol), or **2c** (0.64 mmol) in dry THF (10 mL) under a nitrogen inert atmosphere, NaH (8.5 mmol) was added. The reaction mixture was stirred for 40 min at room temperature. Then, ethyl formate (3.07 mmol) was added dropwise over 30 min. The reaction mixture was stirred at room temperature overnight. Afterward, THF was evaporated under vacuum, and a 1 M HCl solution (18 mL) was added. The precipitate was filtered out and washed with distilled water. Finally, the crude product was purified by column chromatography using a hexane:ethyl acetate (8:2) mixture as the eluent.

##### 18α‐3‐oxo‐11‐deoxy‐2‐formyl‐olean‐12‐en‐30‐oic Acid (4a)

Yield 55%; white powder; melting point: 270–275 °C, ^1^H NMR (600 MHz, CDCl_3_) *δ*_H_ 8.64 (s, 1H), 5.77 (s, 1H), 3.46 (d, *J* = 14.82 Hz, 1H), 2.44 (s, 1H), 2.23 (m, 1H), 2.09–1.97 (m, 2H), 1.94 (d, *J* = 15.0 Hz, 2H), 1.86 (td, *J* = 13.6, 4.4 Hz, 1H), 1.74–1.58 (m, 2H), 1.47 (dtt, *J* = 16.2, 14.3, 7.6 Hz, 5H), 1.38 (s, 3H), 1.23 (s, 3H), 1.20 (s, 3H), 1.17 (s, 3H), 1.14 (s, 3H), 1.13 (s, 3H), 0.86 (s, 3H).

##### 18β‐3‐oxo‐11‐deoxy‐2‐formyl‐olean‐12‐en‐30‐oic Acid (4b)

Yield 50%; white powder; melting point: 159–161 °C Ref[154–158 °C].^[^ ^57^ ^]^

##### 18α‐3‐oxo‐11‐oxo‐2‐formyl‐olean‐12‐en‐30‐oic acid (4c)

Yield 75%; white powder; melting point: >300 °C Ref [319–321°C].^[^ ^56^ ^]^

##### General Procedure for Fischer Indolization (Compounds 3a, 3b, and 3c)

These compounds were prepared from **2a**, **2b**, or **2c**, respectively, following the method described by De‐la‐Cruz‐Martínez et al.^[^ ^23^ ^]^

##### Compound 3a

Yield: 68%; light yellow powder; melting point: 271–273 °C; ^1^H NMR (400 MHz, DMSO‐d_6_): *δ*_H_ 12.06 (bs, 1H), 11.24 (s, 1H), 7.71 (s, 1H), 7.44 (d, *J* = 8.34 Hz, 1H), 7.29 (d, *J* = 8.46 Hz, 1H), 5.30 (s, 1H), 2.82 (d, *J* = 15.06 Hz, 1H), 2.20 (d, *J* = 11.12 Hz, 1H), 2.12–1.94 (m, 5H), 1.82–1.75 (m, 4H), 1.71–1.58 (m, 3H), 1.56–1.36 (m, 4H), 1.33 (s, 3H), 1.23 (s, 3H), 1.19 (s, 3H), 1.16 (s, 1H), 1.10 (s, 3H), 1.03 (s, 3H), 0.91 (s, 3H), 0.79 (s, 3H). ^13^C NMR (101 MHz, DMSO‐d_6_) *δ*_C_ 179.38, 166.51, 159.77, 151.08, 147.06, 143.61, 137.82, 130.23, 126.95, 123.14, 118.90, 116.01, 111.44, 111.15, 106.19, 58.46, 55.37, 52.40, 44.90, 43.34, 41.66, 37.32, 35.30, 33.97, 31.24, 29.83, 22.89, 20.70, 20.43, 18.0, 16.01, 15.77. HRMS (ESI‐MS) *m/z* for C_37_H_49_F_3_NO_2_^+^ [M+H]^+^: calc. 596.3710; found 596.3711.

##### Compound 3b

Yield: 71%; light yellow powder; melting point: 242–245 °C; ^1^H NMR (400 MHz, DMSO‐d_6_) *δ*_H_ 12.09 (bs, 1H, COOH), 11.22 (s, 1H, NH), 7.68 (s, 1H, H–4′), 7.40 (d, *J* = 8.40 Hz, 1H, H‐7´), 7.27 (d, *J* = 8.46 Hz, 1H, H‐6´), 5.27 (s, 1H, H‐12), 2.80 (d, *J* = 15.00 Hz, 1H, H‐1), 2.15 (d, *J* = 15.32, 1H, H‐1), 2.11 (m, 1H, H‐11), 2.09 (d *J=* 13.4 Hz, 1H, H‐18), 1.97 (dd, *J* = 17.7, 8.0 Hz, 1H, H‐22), 1.94 (d, *J=* 13.40 Hz, 1H, H‐7), 1.83 (d, *J* = 10.1 Hz, 1H, H‐9), 1.68–1.35 (m, 4H, H‐21, H‐6, H‐19 and H‐6´), 1.39 (s, 1H, H‐5) 1.30 (s, 3H, H‐24), 1.28 (m, 2H, H‐21 and H‐7), 1.20 (s, 3H, H‐23), 1.16 (s, 3H, H‐26), 1.14 (s, 1H, H‐15), 1.07 (s, 3H, 29‐CH_3_), 1.0 (s, 3H, 27‐CH_3_), 0.88 (s, 3H, 25‐CH_3_), 0.84 (m, 1H, H‐22), 0.77 (s, 3H, 28‐CH_3_).^13^C NMR (101 MHz, DMSO‐d_6_) *δ*_C_ 178.03 (30‐COOH), 144.28 (C3), 143.83 (C13), 137.81 (C7a´), 126.93 (C3a´), 124.54 (q, *J *= 272 Hz, CF_3_), 122.06 (C12), 116.47 (C6´), 114.88 (C4´), 111.00 (C7´), 106.16 (C2), 52.91 (C5), 47.92 (C18), 45.72 (9), 43.22 (C20), 42.48 (C14), 41.36 (C10), 38.11 (C8), 37.60 (C19), 36.35 (C1), 33.96 (C4), 31.70 (C16), 31.62 (C21), 30.38 (C24), 29.62 (C15), 28.23 (C28 and C29), 26.47 (C22), 25.80 (C26), 25.57 (C7), 23.09 (C11), 22.70 (C23), 22.09 (C6), 16.35 (C27), 15.48 (C25). HRMS (ESI‐MS) *m/z* for C_37_H_49_F_3_NO_2_^+^ [M+H]^+^: calc. 596.3710; found 596.3700.

##### Compound 3c

Yield: 81%; light yellow powder; melting point: 296–298 °C; ^1^H NMR (400 MHz, DMSO‐d_6_): *δ*_H_ 12.18 (bs, 1H, 30‐COOH), 11.23 (s, 1H, NH), 7.58 (s, 1H, H‐4^′^), 7.43 (d, *J* = 8.4 Hz, 1H, H‐7^′^), 7.28 (d, *J* = 8.50 Hz, 1H, H‐6^′^), 5.45 (s, 1H, H‐12), 3.67 (d, *J =* 15.42 Hz, 1H, H‐1), 2.63 (s, 1H, H‐9), 2.33 (d, *J* = 11.2 Hz, 1H, H‐18), 2.26 (d, *J=* 15.6 Hz, 1H, H‐1), 1.94 (dd, *J =* 14.3, 10.5 Hz, 1H, H‐15), 1.76 (m, 3H, H‐7, H‐16 and H‐22), 1.64 (m, 1H, H‐6), 1.56 (m, 3H, H‐6, H‐7 and H‐19), 1.50 (m, 1H, H‐22), 1.42 (m, 3H, H‐5, H‐16 and H‐22), 1.39 (s, 3H, 27‐CH_3_), 1.29 (s, 3H, 28‐CH_3_), 1.23 (s, 6H, 23‐CH_3_ and 24‐CH_3_), 1.19 (s, 3H, 29‐CH_3_), 1.13 (s, 3H, 26‐CH_3_), 1.11 (s, 3H, 25‐CH_3_), 0.68 (s, 3H, 28‐CH_3_). ^13^C NMR (101 MHz, DMSO‐d_6_): *δ*_C_ 198.52 (C11), 179.47 (30‐COOH),166.48 (C13), 143.57 (C3), 137.86 (C3a’), 126.83 (C7a’), 124.48 (q, *J* = 272 Hz, CF_3_), 123.14 (C12), 116.48 (C6’), 111.07 (C7’), 106.10 (C2), 58.41 (C9), 52.27 (C5), 44.82 (C14), 43.33 (C8),41.61 (C20), 39.90 (C18), 37.31 (C10), 36.48 (C1), 35.28 (C21), 35.24 (C22), 33.90 (C4), 32.47(C7), 31.15 (C19), 30.43 (C23), 29.82 (C17), 28.39 (C16), 26.34 (C15), 22.89 (C24), 20.64(C29), 20.42 (C27), 18.11 (C6), 17.99 (C26), 16.00 (C25), 15.70 (C28). HRMS (ESI‐MS) *m/z* for C_37_ H_47_F_3_NO_3_^+^ [M+H]^+^: calc. 610.3503; found 610.3510.

##### Synthesis of N‐Phenylpyrazole GA Derivatives 5a, 5b, 5c

These compounds were prepared from **4a**, **4b**, or **4c**, respectively, following the procedure described by De‐la‐Cruz‐Martínez et al.^[^ ^23^ ^]^

##### Compound 5a

Yield 20%; white powder; melting point >300 °C; ^1^H NMR (600 MHz, CDCl_3_): *δ*_H_ 12.05 (bs, 1H), 7.30 (s, 1H), 7.30 (d, *J* = 8.94 Hz, 2H), 7.23 (d, *J* = 8.04 Hz, 2H), 5.25 (s, 1H), 2.57 (d, *J* = 14.88 Hz, 1H), 2.39 (s, 3H), 2.08 (d, *J* = 14.8 Hz, 1H), 1.98 (d, *J* = 12.3 Hz, 2H), 1.93 (d, *J* = 13.2 Hz, 2H), 1.80–1.70 (m, 4H), 1.63 (t, *J* = 13.38 Hz, 1H), 1.50 (m, 2H), 1.40 (m, 2H), 1.33–1.23 (m, 4H), 1.28 (s, 3H), 1.15 (s, 3H), 0.99 (s, 3H), 0.98 (s, 3H), 0.95 (s, 3H), 0.90 (s, 3H), 0.85 (d, *J = *13.14 Hz, 1H), 0.76 (s, 3H). ^13^C NMR (101 MHz, CDCl_3_) *δ*_C_ 183.49_,_ 165.35, 140.50, 134.71, 129.86, 129.22, 129.02, 128.67, 128.26, 124.61, 122.62, 119.05, 118.54, 110.32, 107.05, 59.65, 53.59, 44.89, 44.15, 42.43, 40.37, 37.98, 37.69, 35.98, 35.82, 34.18, 33.32, 31.77, 31.23, 28.58, 26.95, 23.68, 21.63, 20.96, 20.74, 18.53, 16.36, 16.20. HRMS (ESI‐MS) *m/z* for C_38_H_53_N_2_O_2_^+^ [M+H]^+^: calc. 569.4102; found 569.4106.

##### Compound 5b

Yield 7%; white powder; melting point 290–293 °C; ^1^H NMR (600 MHz, DMSO‐d6): *δ*_H_ 7.28 (d, *J *= 8.94 Hz, 2H), 7.28 (s, 1H), 7.23 (d, *J *= 7.98 Hz, 2H), 5.25 (s, 1H), 2.59 (d, *J *= 14.94 Hz, 1H), 2.54 (s, 1H), 2.39 (s, 3H), 2.08 (d, *J *= 14.94 Hz, 1H), 2.0–1.90 (m, 4H), 1.82–1.67 (m, 5H), 1.62 (t, *J* = 13.26 Hz, 1H), 1.57–1.33 (m, 5H), 1.33–1.23 (m, 4H), 1.15 (s, 3H), 1.07 (s, 3H), 1.01 (s, 1H), 0.99 (s, 3H), 0.98 (s, 3H), 0.95 (s, 3H), 0.9 (s, 3H), 0.85 (d, *J* = 12.30 Hz, 1H). 0.76 (s, 3H). HRMS (ESI‐MS) *m/z* for C_38_H_53_N_2_O_2_^+^ [M+H]^+^: calc. 569.4102; found 569.4106. Single crystal X‐ray diffraction: see Figure 2 and Table S2, Supporting Information.

##### Compound 5c

Yield 32%; white powder; melting point >300 °C; ^1^H NMR (400 MHz, DMSO‐d_6_): *δ*_H_ 7.29 (d, *J = *7.72 Hz, 2H, H–2′ and H–6′), 7.28 (s, 1H, pyrazole–H), 7.24 (d, *J = *8.24 Hz, 2H, H–3′ and H–5′), 5.43 (s, 1H, H–12), 3.44 (d, *J = *15.16 Hz, 1H, H‐1), 2.53 (s, 1H, H‐9), 2.39 (s, 3H, phenyl‐CH_3_), 2.32 (d, *J = *10.3 Hz, 1H, H‐18), 2.17 (d, *J = *15.3 Hz, 1H, H‐1), 1.79 (t, *J* = 11.9 Hz, 1H, H‐19), 1.73–1.40 (m, 8H, H‐6, H‐7, H‐15, H‐16, H‐19 and H‐21), 1.36 (s, 3H, 29‐CH_3_), 1.32–1.20 (m, 7H, H‐5, H‐15, H‐21 and H‐22), 1.17 (s, 3H, 26‐CH_3_), 1.12 (s, 3H, 27‐CH_3_), 1.09 (s, 3H, 25‐CH_3_), 0.99 (s, 3H, 23‐CH_3_), 0.96 (s, 3H, 24‐CH_3_), 0.86 (dd, *J* = 9.2, 5.1 Hz, 1H, H‐16), 0.67 (s, 3H, 28‐CH_3_). ^13^C NMR (101 MHz, DMSO‐d_6_) *δ*_C_ 198.40 (C11), 166.48 (C13), 145.02 (C3), 139.71 (C1’), 138.49 (C2), 137.50 (C‐pyrazole), 128.95 (C3’ and C5’), 128.84 (C2’ and C6’), 123.14 (C12), 113.40 (C4’), 68.52 (C10), 58.37 (C9), 55.84 (C14), 53.57 (C5), 44.78 (C14), 43.13 (C8), 41.61 (C19), 39.21 (C18), 37.34 (C1), 36.57 (C4), 35.29 (C22), 34.18 (C7), 32.44 (C17), 31.51 (C16), 29.17 (C23), 28.39 (C15), 26.36 (C29), 22.39 (C24), 20.75 (C26), 20.68 (C21), 20.33 (phenyl‐CH_3_), 18.02 (C6), 17.91 (C25), 15.73 (C27), 15.66 (C28). HRMS (ESI‐MS) *m/z* for C_38_H_51_N_2_O_3_^+^ [M+H]^+^: calc. 583.3895; found 583.3881.

## 5 X‐Ray Diffraction

Single crystals of **4d** and **5b** were obtained by recrystallization from a mixture of hexane and ethyl acetate solution. Data were collected using an Agilent Xcalibur Gemini CCD diffractometer using graphite‐monochromated MoK*α* (*λ* = 0.71073 Å) radiation in the *ω* scan mode at 293 K by using Olex2.^[^ ^58^ ^]^ The structures were solved with the ShelXT^[^ ^59^ ^]^ structure solution program using intrinsic phasing and refined with the ShelXL^[^ ^60^ ^]^ refinement package using least squares minimization. The nonhydrogen atoms were treated anisotropically. Hydrogen atoms were placed in their calculated positions and then refined using the riding model, whereas the hydrogens from the –OH groups were localized from the difference electron density map, and their positions were refined with U_iso_ tied to the parent atom with distance restraints. The absolute configuration of **4d** was established by the known configuration of the 18*β*‐GA acid acquired from Sigma–Aldrich and used as a starting material (Scheme 1). Table S1 and S2, Supporting Information show relevant crystal data.

## 6 Biological Assays

### 6.1 Protein Tyrosine Phosphatase 1B (PTP1B) Activity Assay

Recombinant human protein tyrosine phosphatase 1B, consisting of 400 and 285 amino acid residues, was used.^[^ ^35^ ^]^ The newly synthesized compounds and positive controls (SOV, AU, and SU) were dissolved in DMSO or Tris buffer solution (50 mM, pH 6.8). Aliquots of 0–10 µL of the test compounds (triplicate) were taken and incubated for 15 min at 37 °C with 85 µL of Tris buffer solution (50 mM, pH 6.8) containing PTP1B enzyme and 5 µL of *p*‐nitrophenyl phosphate substrate (*p*‐NPP, 10 mM). Absorbance was then measured at a wavelength of 405 nm. The inhibitory activity was determined according to Equation (1).

(1) $$\%\;\text{PTP}1B=\left(\frac{A_{405b}}{A_{405c}}\right)\times 100$$

where % PTP1B: Is the percentage of inhibition. *A*_405c_: Is the corrected absorbance of the blank (*A*_405initial_–*A*_405final_). *A*_405b_: Is the corrected absorbance of the compounds (*A*_405control_–*A*_405compound_). The IC_50_ was calculated using regression analysis, Equation (2):

(2) $$\%\text{inhibition}=\frac{A_{100}}{1+{\left(\frac{I}{{\mathrm{IC}}_{50}}\right)}^{s}}$$

where *A*_100_: Maximum inhibition, *I*: Inhibitor concentration, IC_50_: Concentration required to inhibit enzymatic activity by 50% ± SD, *s*: Hill slope.

### 6.2 Kinetic Studies of PTP1B

Assays to determine enzyme kinetic parameters and inhibition mechanism were conducted under the same experimental conditions as the IC_50_ assays, but with variable concentrations of *p*‐nitrophenyl phosphate (*p*‐NPP) ranging from 0.1 to 0.5 mM in increments of 0.1 mM, and five increasing concentrations of each inhibitor were tested according to their previously determined IC_50_ value: 18α‐GA 0, 1.5, 2.25, 3, and 3.7 µM; 18β‐GA 0, 5.0, 12.5, 15, and 20 µM; **3b** 0, 0.5, 1.0, 2.0, and 3.0 µM; **5c** 0, 1.0, 2.0, 3.0, and 4.0 µM. The negative control was prepared with the enzyme and substrate in the absence of the inhibitor. Experiments were conducted in triplicate.^[^ ^30^ ^]^ The Supporting Information contains detailed Equations (E1–E5) used to determine the kinetic parameters and mechanisms underlying PTP1B inhibition. The kinetic parameters of enzyme inhibition, *K*_m_ and *V*_max_, were determined by fitting the data to the Michaelis‐Menten model, Equation (3) (OriginPro 2018 (64‐bit) SR1).

(3) $$y=\frac{V_{\max}X}{K_{m}+X´}$$

where *V*_max_: is the maximum velocity, *X*: is the inhibitor concentration, *K*_m_: Michaelis constant.

Data fitting determined the inhibition mechanisms of PTP1B to a nonlinear regression curve, Equation (4) for competitive inhibition model using OriginPro 2018 (64‐bit) SR1.

(4) $$y=\frac{V_{\max}(X)}{\left(\frac{K_{m}}{1+\frac{I}{K_{i}}}\right)+X}$$

where *V*_max_: maximum velocity, *X*: substrate concentration, *I*: inhibitor concentration, *K*_m_: Michaelis constant, *K* *_i_*: inhibition constant.

### 6.3 T‐Cell Protein Tyrosine Phosphatase Activity (TCPTP) Assay

TCPTP enzyme reported by *Mendoza* et al. was used for this assay. The newly synthesized compounds and positive control SOV were dissolved in DMSO or Tris buffer solution (50 mM, pH 6.8). Aliquots of 0–10 µL (triplicate) of the newly synthesized compounds, positive control, and solvent were taken. These were incubated with 85 µL of enzyme solution (Tris, 50 mM, pH 6.8, TCPTP) and 5 µL of substrate (*p*‐NPP, 10 mM) for 15 min at 37 °C. After incubation, absorbance was measured at a wavelength of 405 nm.^[^ ^61^ ^]^

### 6.4 Culture Conditions

For the resistant insulin model using HepG2 cells, the following reagents were acquired from Sigma–Aldrich (Toluca, MEX, Mexico, and St. Louis, MO, USA): Insulin (I2643), Dulbecco's modified eagle's medium (DMEM) low glucose media (D5523), Trypsin‐EDTA solution (T4174), antibiotic‐antimycotic solution(A5955), dimethyl sulfoxide (DMSO‐D8418), sodium deoxycholate (D6750), triethylammonium bicarbonate buffer 1 M (TEAB‐T7408), glutaraldehyde 25% (G6275), acetic acid (695092), and crystal violet (C0775). The bicinchoninic acid protein quantitation kit (23225) and the Halt Cocktail with protease and phosphatase inhibitors (1861281) were acquired from ThermoFisherScientific. Fetal bovine serum (26140–079) was from Gibco. The chemiluminescent solution (170–5061) was acquired from Biorad. Rabbit anti‐PTP1B (5311S) and mouse anti‐*β* actin (3700S) primary antibodies were obtained from Cell Signaling, and secondary antibodies coupled with horseradish peroxidase were from Jackson laboratories (rabbit‐111−035, mouse‐115–035).

### 6.5 Cell Viability with Crystal Violet

A 96‐well plate was seeded with 3,000 cells per well and incubated for 24 h. Afterward, the cells were treated with test compounds at various concentrations for 24 h. After incubation, the media was removed, and 100 µL of 1% glutaraldehyde was added for 15 min. Afterward, the liquid was removed, and 50 µL of 0.5% crystal violet (dissolved in 25% methanol) was added for 30 min. The crystal violet staining solution was removed, and the plate was thoroughly washed with tap water. The precipitate was dissolved in a 10% acetic acid solution and mixed for 15 min. The absorbance was then measured at 590 nm using a plate reader (xMark Microplate Spectrophotometer—Bio‐Rad).

### 6.6 Protein Extraction and Western Blotting

Treated cells with compounds were washed twice with 1 mL of PBS, and protein was extracted using 500 µL of a buffer solution containing 10% sodium deoxycholate in 10% TEAB, supplemented with a protease and phosphatase inhibitor cocktail. The extracts were heated to 80 °C for 5 min and disaggregated through passages with an insulin syringe. The cell extracts were centrifuged at 15,000 rpm for 15 min at 4 °C, and the supernatant was collected. Protein quantification was performed using the bicinchoninic acid assay method, and aliquots were prepared for western blotting. SDS/PAGE was conducted, and proteins were transferred onto a nitrocellulose membrane. The membrane was then blocked with 10% skimmed milk in TBST for 1 h and incubated overnight with primary antibodies (1:400 dilution). Secondary antibodies coupled to horseradish peroxidase were diluted 1:2000, and membranes were incubated for 1 h. The blots were documented using the ChemiDoc MP (Bio‐Rad), and densitometric analysis was performed using ImageJ (U.S. National Institutes of Health). Statistical analyses were carried out using GraphPad Prism 8.0.

## 7 Computational Studies

### 7.1 Structural Models of PTP1B_1–400_, TCPTP_1–415_, and PTP1B_1–400_‐pNPP Complex

The 3D structures of PTP1B_1–400_ and TCPTP_1–415_ were retrieved from the AlphaFold Protein Structure Database developed by DeepMind and EMBL‐EBI (https://alphafold.ebi.ac.uk/). The UniProt code P18031 corresponds to the *PTPN1* gene, and the P17706 code to the *PTPN2* gene corresponds to the proteins *h*PTP1B_1–400_ and *h*TCPTP_1–415_, respectively. Subsequently, these 3D structures were submitted to MDS using the AMBER11 force field to obtain a folding with biological relevance of the unstructured C‐terminal zone (amino acids 314–415). To do so, 270 and 200 ns of MDS were performed for PTP1B_1–400_ and TCPTP_1–415_, respectively. Afterward, both models were refined by running the md_refine macro, which uses the YASARA2^[^ ^62^ ^]^ force field. The snapshot with the minimum energy and maximum quality score was selected. For the construction of the PTP1B_1–400_‐pNPP complex, utilized as the uncompetitive model for docking simulations, the ligand pNPP was first docked into the catalytic site of the PTP1B_1−400_ structure using AutoDock Vina,^[^ ^38^ ^]^ which is integrated into YASARA Structure. A cube simulation cell with an extension of 6 Å was created around residue Cys^215^, and the resulting file was saved in *.sce format. Concurrently, the pNPP ligand was constructed as described in the Molecular Docking section. The ligand was then saved in *.sdf format. With the PTP1B_1–400_ structure and the pNPP ligand prepared, molecular docking was performed using the modified *dock_run* macro set for 500 runs. The resulting structure with the best binding energy and the largest population was selected. With the newly created 3D model of the PTP1B‐pNPP complex, a new MDS was conducted using the AMBER11 force field, with a simulation time of 200 ns. Afterward, the structure was further refined using the *md_refine* macro. The snapshot with the minimum energy and maximum quality score was selected. Finally, the quality of both PTP1B_1–400_‐pNPP complex and TCPTP_1–415_ structure was assessed with Molprobity^[^ ^63^ ^]^ server (http://molprobity.biochem.duke.edu/) and Swiss Model Structure Assessment^[^ ^64^ ^]^ server (https://swissmodel.expasy.org/assess).

### 7.2 Molecular Docking

For molecular docking using Autodock^[^ ^37^ ^]^ 4.2 and Autodock Vina,^[^ ^38^ ^]^ the graphical interface AutoDockTools^[^ ^37^ ^]^ 1.5.6 suite was used to prepare and analyze the docking simulations. pNPP and GA ligands were constructed using Chem3D BioUltra 16.0 software (PerkinElmer, Waltham, MA, USA). The GA derivatives were prepared by systematically modifying the structure of 18*α*‐GA (deposition number 1,199,252^[^ ^65^ ^]^) and 18β‐GA (deposition number 1,169,430^[^ ^66^ ^]^) were retrieved from the Cambridge Crystallographic Data Base (https://www.ccdc.cam.ac.uk/). Afterward, the protonation state of all compounds was fixed, assuming a pH = 7.4, and then the 3D geometry was optimized by the Universal Force Field (UFF) in AVOGADRO^[^ ^67^ ^]^ 1.2.0 (http://avogadro.cc/).

Using AutoDockTools, hydrogen atoms were added to the macromolecules, and Gasteiger–Marsili charges were assigned to the atoms in the protein and ligands. Both protein and ligands were exported as *.pdbqt files. Blind docking simulations using Autodock 4.2 for the short form of PTP1B (PTP1B_1–298_, using PDB ID: 1C83^[^ ^68^ ^]^ macromolecule) were performed with a grid box size: 126 Å × 126 Å × 126 Å with a spacing of 0.375 Å and coordinates *x* = 43.778, *y* = 17.255 and *z* = 15.033; while for modeled PTP1B_1–400_‐pNPP complex were performed with a grid box size: 126 Å × 126 Å × 126 Å with a spacing of 0.375 Å and coordinates *x* = −2.945, *y* = −1.919 and *z* = 3.952; finally for modeled TCPTP were performed with a grid box size: 126 Å × 126 Å × 126 Å with a spacing of 0.503 Å and coordinates *x* = 13.883, *y* = 0.817 and *z* = −0.307 whereas docking simulations at the allosteric site of PTP1B_1–298_ (using 1C83 macromolecule) were performed with a grid box size: 70 Å × 60 Å × 60 Å with a spacing of 0.375 Å and coordinates *x* = 44.226, *y* = 11.692 and *z* = 3.767 while for PTP1B_1–400_‐pNPP were performed with a grid box size: 80 Å × 90 Å × 90 Å with a spacing of 0.375 Å and coordinates *x* = −13.917, *y* = 10.765 and *z* = 30.462; finally for modeled TCPTP were performed with a grid box size: 70 Å × 70 Å × 70 Å with a spacing of 0.375 Å and coordinates *x* = 12.628, *y* = 2.756 and *z* = 8.268 The search was carried out using the Lamarckian Genetic Algorithm. 100 GA runs with a maximum number of 25,000,000 evaluations, a mutation rate of 0.02, and an initial population of 150 conformers were covered. Finally, each ligand with the best cluster size and the lowest binding energy was selected for further analysis. Molecular docking using Autodock Vina was carried out employing the exact coordinates as previously mentioned, except that the grid box dimensions were 47.25 Å × 47.25 Å × 47.25 Å (for PTP1B_1–298_ macromolecule and modeled PTP1B_1–400_‐pNPP in the blind docking simulations) whereas docking simulations at the allosteric site of PTP1B_1–298_ was carried out employing the same coordinates as previously mentioned, except that the grid box dimensions were: 26.25 Å × 22.5 Å × 22.5 Å, while for modeled PTP1B_1–400_‐pNPP, the grid box dimensions were: 30 Å × 33.75 Å × 33.75 Å. Finally, for the modeled TCPTP, the grid box dimensions were 26.25 Å × 26.25 Å × 26.25 Å, and the exhaustiveness value was set to 500.

The PTP1B_1–298_, PTP1B_1–400_‐pNPP and TCPTP 3D structures were exported to GOLD^[^ ^39^ ^]^ software. Using the GOLD wizard, the proteins were prepared by adding hydrogens and extracting the ligands, which were further docked at the catalytic site or allosteric site within an 8 Å radius sphere that was carried out using the following parameters: 100 genetic algorithm runs and 125,000 operations. CHEMPLP fitness was chosen as the main scoring function, whereas GoldScore fitness was selected as the re‐scoring function. The dockings were ranked according to the value of the CHEMPLP and GoldScore fitness function.

### 7.3 Molecular Dynamics Simulations (MDS)

The ligand‐protein complexes were submitted to MD simulations with YASARA Structure. The simulations began with an optimization of the hydrogen bonding network to enhance solute stability and a pKa prediction to fine‐tune the protonation states of protein residues at the chosen pH of 7.4. Then, NaCl ions were added with a physiological concentration of 0.9%, with an excess of either Na or Cl to neutralize the cell. After the steepest descent and simulated annealing minimizations to remove clashes, the simulation was run for 200 nanoseconds using the AMBER11 force field for the solute, GAFF2^[^ ^69^ ^]^ and AM1BCC^[^ ^70^ ^]^ for ligands and TIP3P for water. The cutoff was 8 Å for van der Waals forces (the default used by AMBER); no cutoff was applied to electrostatic forces (using the Particle Mesh Ewald algorithm).^[^ ^71^ ^]^ The equations of motion were integrated using a multiple timestep of 1.25 fs for bonded interactions and 2.5 fs for nonbonded interactions at a temperature of 310 K and a pressure of 1 atm (NPT ensemble), employing algorithms described in detail previously. After inspecting the solute RMSD as a function of simulation time, the first 100 picoseconds were considered equilibration time and excluded from further analysis. The binding energy study using the MM‐PBSA method was performed by running the *md_analyzebindingenergy* macro, which was previously modified with the PBS method at a temperature of 310 K.

## Conflict of Interest

The authors declare no conflict of interest.

## Author Contributions

**Ledy De‐la‐Cruz‐Martínez**: data curation (lead); formal analysis (lead); investigation (lead); methodology (lead); writing—original draft (lead); and writing—review and editing (lead). **Rosendo Martínez‐Arellano**: data curation (lead) and investigation (lead). **Mitzi López‐Sánchez**: data curation (lead) and investigation (lead). **José G. Alvarado‐Rodríguez**: data curation (equal); formal analysis (supporting); and methodology (supporting). **Jesús Martin Torres‐Valencia**: investigation (supporting). **David Equihua‐González**: investigation (supporting). **Julio‐César Almanza‐Pérez**: supervision (supporting). **Jaime Pérez‐Villanueva**: resources (equal). **Martín González‐Andrade**: data curation (lead); funding acquisition (lead); investigation (lead); resources (lead); and supervision (lead). **José C. Páez‐Franco**: data curation (lead); investigation (lead); resources (lead); and supervision (lead). **Francisco Cortes‐Benitez**: conceptualization (lead); investigation (lead); project administration (lead); resources (lead); supervision (lead); and writing—review and editing (lead).

## Patents

The patent application MX/a/2022/004731 is owned by Francisco Cortés‐Benítez, Martín González‐Andrade, and Jaime Pérez‐Villanueva.

## Supporting information

Supplementary Material

## Data Availability

The data that support the findings of this study are available in the supplementary material of this article.
