# Supplementary material for: Impact of C18 Epimerization of Indole‐ and Pyrazole‐Fused 18β‐Glycyrrhetinic Acid Derivatives on PTP1B and TCPTP Inhibitory Activity: Synthesis, In Vitro, and In Silico Studies
Source: ChemMedChem. 2025 Sep 14;20(22):e202500350. doi: 10.1002/cmdc.202500350 (PMC12640663; doi:10.1002/cmdc.202500350)
Supplement: Supplementary file 1 — Supplementary Material [file CMDC-20-e202500350-s001.pdf]

Supporting Information

Impact of C18 Epimerization of Indole- and Pyrazole-fused 18β-Glycyrrhetinic Acid Derivatives on PTP1B and TCPTP Inhibitory Activity. Synthesis, In Vitro, and In Silico Studies.

Ledy De-la-Cruz-Martínez,[a,b,e] Rosendo Martínez-Arellano,[b,f] Mitzi López-Sánchez,[b,f] José G. Alvarado-Rodríguez,[c] Jesús Martin Torres-Valencia,[c] David Equihua-González,[b] Julio-César Almanza-Pérez,[d] Jaime Pérez-Villanueva,[b] Martín González-Andrade,*[e] José C. Páez-Franco,*[f] Francisco Cortés-Benítez*[b]

[a] *Doctorado en Ciencias Farmacéuticas, División de Ciencias Biológicas y de la Salud, Universidad Autónoma Metropolitana – Unidad Xochimilco, Ciudad de México 04960, Mexico.*

[b] *Laboratorio de Síntesis y Aislamiento de Sustancias Bioactivas, Departamento de Sistemas Biológicos, División de Ciencias Biológicas y de la Salud, Universidad Autónoma Metropolitana – Unidad Xochimilco, Ciudad de México 04960, Mexico.*

[c] *Área Académica de Química, Universidad Autónoma del Estado de Hidalgo, Hidalgo 42184, Mexico.*

[d] *Laboratorio de Farmacología, Departamento de Ciencias de la Salud, D.C.B.S., Universidad Autónoma Metropolitana - Unidad Iztapalapa, Ciudad de México 09340, Mexico.*

[e] *Laboratorio de Biosensores y Modelaje Molecular, Departamento de Bioquímica, Facultad de Medicina, Universidad Nacional Autónoma de México, Ciudad de México 04510, Mexico.*

[f] *Red de apoyo a la Investigación, Universidad Nacional Autónoma de México, Instituto Nacional de Ciencias Médicas y Nutrición Salvador Zubirán, Ciudad de Mexico 14080, Mexico*

*Correspondence:

Francisco Cortés-Benítez: jcortesb@correo.xoc.uam.mx; Tel.: +52 (55) 5483 7000, ext. 7259.

Martin González-Andrade: martin@bq.unam.mx; Tel.: +52 (55) 56232254

José C. Páez-Franco: paez@cic.unam.mx; Tel.: +52 (55) 54876080

Table of content

Figure S1. ^{1}H NMR (600 MHz) spectrum of methyl-18α-3β-hydroxy-olean-12-en-30-oate in $CDCl_3$ S1

Figure S2. ^{13}C NMR (126 MHz) spectrum of methyl-18α-3β-hydroxy-olean-12-en-30-oate in $CDCl_3$ S1

Figure S3. COSY spectra of methyl-18α-3β-hydroxy-olean-12-en-30-oate S2

Figure S4. HMBC spectra of methyl-18α-3β-hydroxy-olean-12-en-30-oate S4

Figure S5. HSQC spectra of methyl-18α-3β-hydroxy-olean-12-en-30-oate S5

Figure S6. NOESY spectra of methyl-18α-3β-hydroxy-olean-12-en-30-oate S6

Figure S7. ^{1}H NMR (400 MHz) spectrum of **18α-GA** in DMSO-d_6 S7

Figure S8. ^{13}C NMR (101 MHz) spectrum of **18α-GA** in DMSO-d_6 S7

Figure S9. COSY spectra of **18α-GA** S9

Figure S10. HMBC spectra of **18α-GA** S10

Figure S11. HSQC spectra of **18α-GA** S11

Figure S12. ^{1}H NMR (600 MHz) spectrum of **18β-GA** in $CDCl_3$ S12

Figure S13. ^{13}C NMR (126 MHz) spectrum of **18β-GA** in $CDCl_3$ S12

Figure S14. COSY spectra of **18β-GA** S13

Figure S15. HMBC spectra of **18β-GA** S14

Figure S16. HSQC spectra of **18β-GA** S15

Figure S17. NOESY spectra of **18β-GA** S16

Figure S18. ^{1}H NMR (600 MHz) spectrum of **2a** in $CDCl_3$ S17

Figure S19. ^{1}H NMR (600 MHz) spectrum of **2b** in $CDCl_3$ S17

Figure S20. ^{13}C NMR (151 MHz) spectrum of **2b** in $CDCl_3$ S18

Figure S21. ^{1}H NMR (600 MHz) spectrum of **3a** in DMSO-d_6 S18

Figure S22. ^{13}C NMR (101 MHz) spectrum of **3a** in DMSO-d_6 S19

Figure S23. ^{1}H NMR (400 MHz) spectrum of **3b** in DMSO-d_6 S19

Figure S24. ^{13}C NMR (101 MHz) spectrum of **3b** in DMSO-d_6 S20

Figure S25. COSY spectra of **3b** compound S22

Figure S26. HMBC spectra of **3b** compound S24

Figure S27. HSQC spectra of **3b** compound S25

Figure S28. ^{1}H NMR (400 MHz) spectrum of **3c** in DMSO-d_6 S26

Figure S29. ^{13}C NMR (101 MHz) spectrum of **3c** in DMSO-d_6 S26

Figure S30. COSY spectra of **3c** compound S28

Figure S31. HMBC spectra of **3c** compound S30

Figure S32. HSQC spectra of **3c** compound S32

Figure S33. ^{1}H NMR (400 MHz) spectrum of **FC-114** in DMSO-d_6 S32

Figure S34. ^{13}C NMR (101 MHz) spectrum of **FC-114** in DMSO-d_6 S33

Figure S35. ^{1}H NMR (600 MHz) spectrum of **4a** in $CDCl_3$ S33

Figure S36. ^{1}H NMR (600 MHz) spectrum of **5a** in DMSO-d_6 S34

Figure S37. ^{13}C NMR (101 MHz) spectrum of **5a** in $CDCl_3$ S34

Figure S38. ^{1}H NMR (600 MHz) spectrum of **5b** in DMSO-d_6 S35

Figure S39. ^{1}H NMR (400 MHz) spectrum of **5c** in DMSO-d_6 S36

Figure S40. ^{13}C NMR (101 MHz) spectrum of **5c** in DMSO-d_6 S36

Figure S41. COSY spectra of **5c** compound S38

Figure S42. HMBC spectra of **5c** compound S40

Figure S43. HSQC spectra of **5c** compound S42

Figure S44. ^{1}H NMR (400 MHz) spectrum of compound **FC-122** in DMSO-d_6 S42

Table S1. Crystal data, structure solution, and refinement parameters for compound **4d** S43

Table S2. Crystal data, structure solution, and refinement parameters for compound **5b** S44

Figure S45. Mass spectrum formula report of compound **3a** S45

Figure S46. Mass spectrum formula report of compound **3b** S45

Figure S47. Mass spectrum formula report of compound **3c** S46

Figure S48. Mass spectrum formula report of compound **5a** S46

Figure S49. Mass spectrum formula report of compound **5b** S47

Figure S50. Mass spectrum formula report of compound **5c** S47

Figure S51. Selectivity of the inhibition for PTP1B over TCPTP for GA and its derivatives at 100 μM S49

Figure S52. Lineweaver–Burk plots for PTP1B inhibition by AG and its derivatives S52

Table S3. Quality parameters of the generated $PTP1B_{1\text{-}400}$ modeled S54

Figure S53. Validation of the $PTP1B_{1-400}$ modeled S55

Table S4. Quality parameters for the $TCPTP_{1-415}$ modeled S56

Figure S54. Validation of the $TCPTP_{1-415}$ modeled S57

Figure S55. Per-residue contacts with compound **3c** from MD simulations S58

Figure S56. Per-residue contacts with compound **5c** from MD simulations S58

Figure S57. Per-residue contacts with compound **FC-114** from MD simulations S59

Figure S58. Per-residue contacts with compound **FC-122** from MD simulations S59

Figure S59. Cell viability assessment by crystal violet staining S60

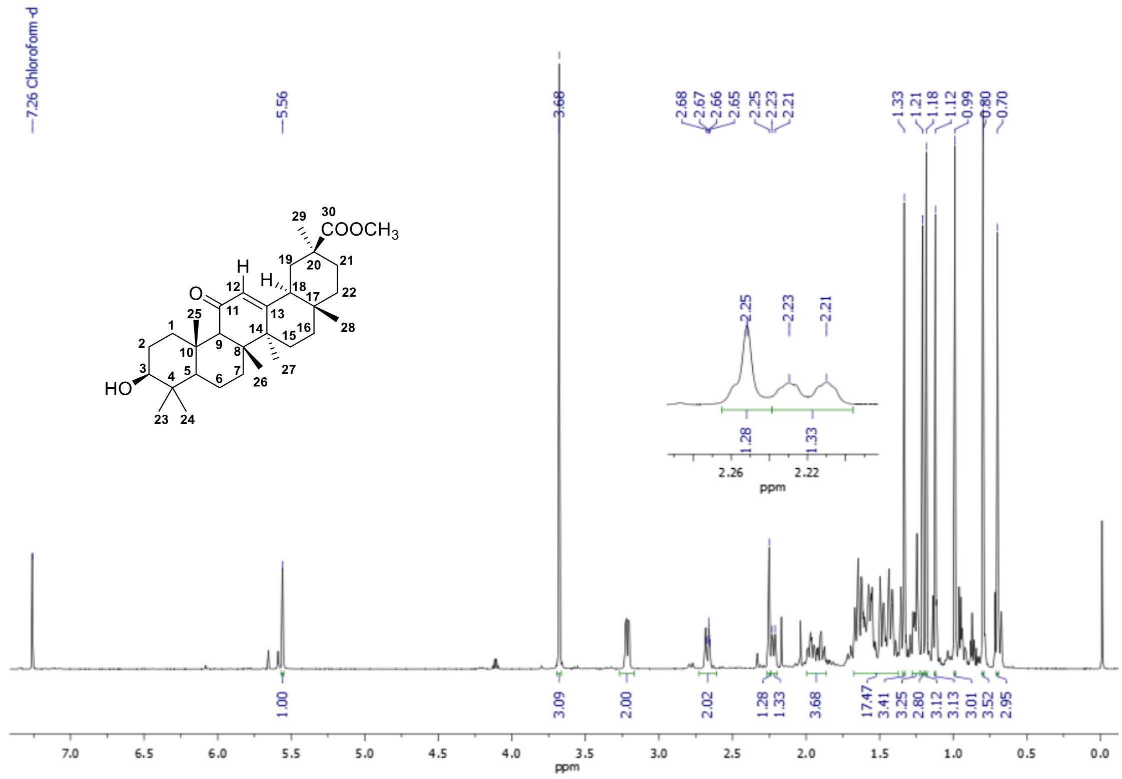

Figure S1. ^{1}H NMR (600 MHz) spectrum of methyl-18α-3β-hydroxy-olean-12-en-30-oate in $CDCl_3$

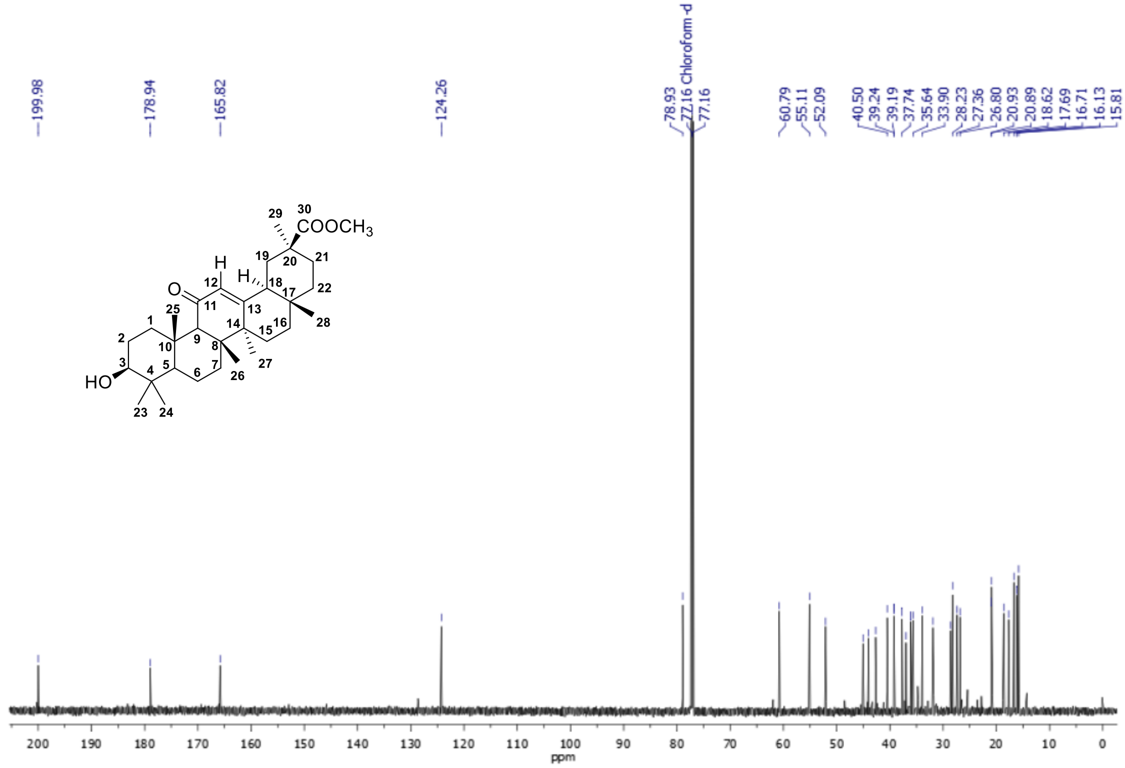

Figure S2. ^{13}C NMR (126 MHz) spectrum of methyl-18α-3β-hydroxy-olean-12-en-30-oate in $CDCl_3$

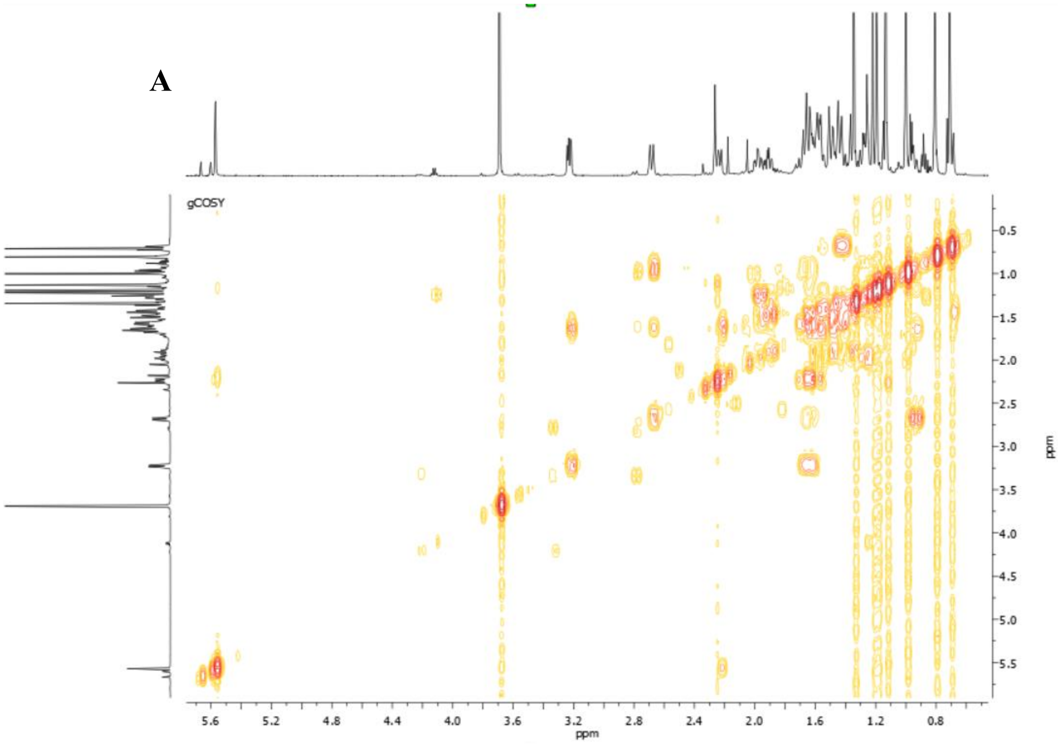

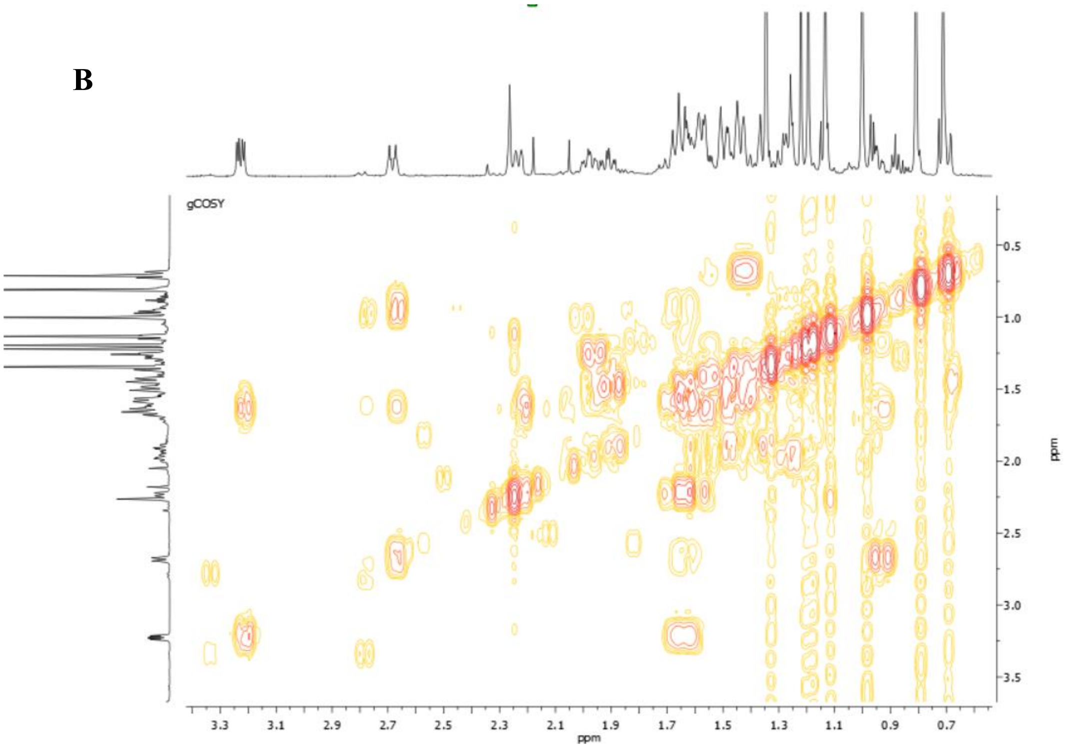

Figure S3. A) Full COSY spectra of methyl-18α-3β-hydroxy-olean-12-en-30-oate in $CDCl_3$. **B)**. Highfield spectra.

A

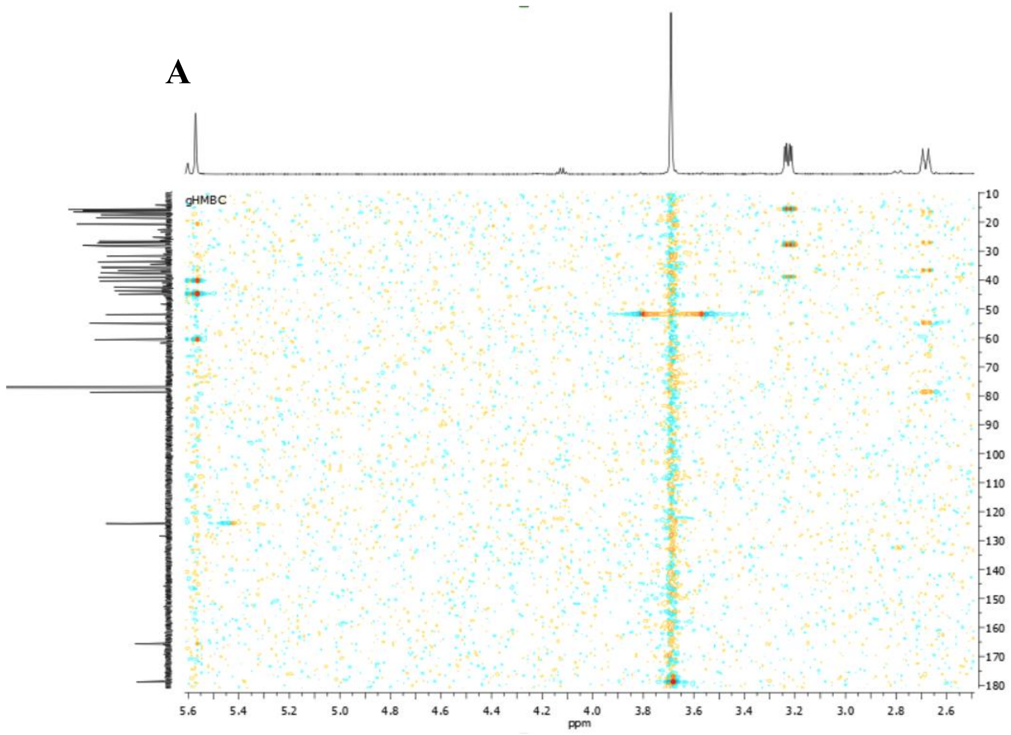

B

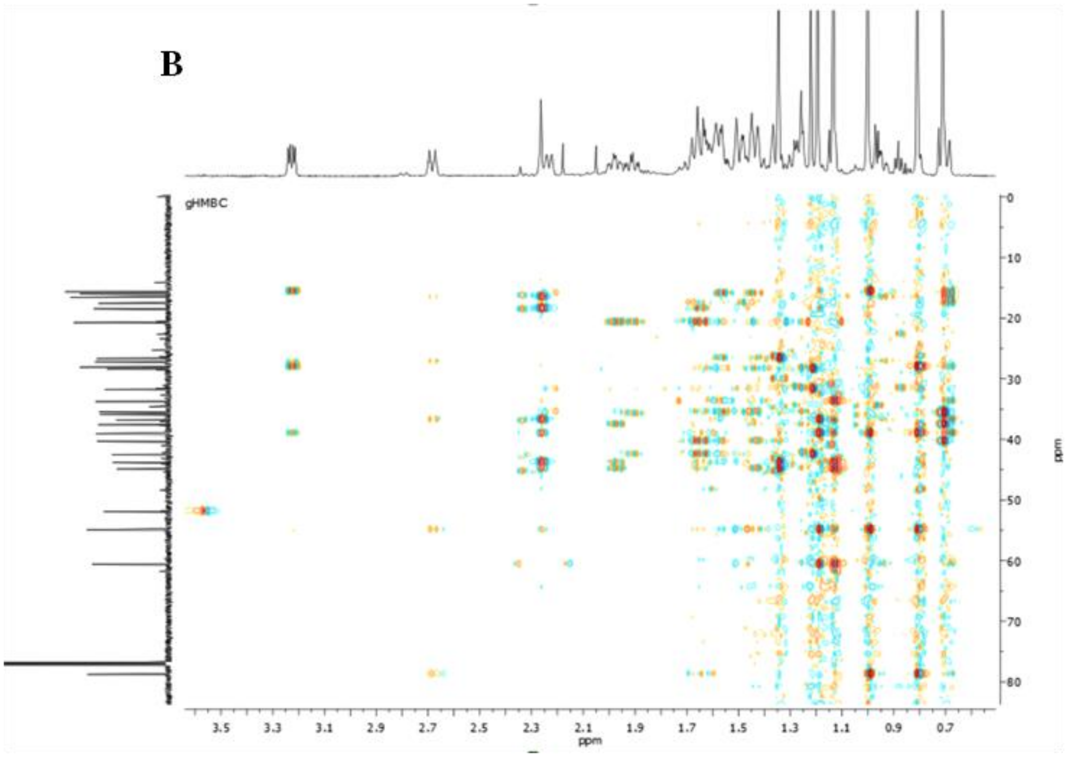

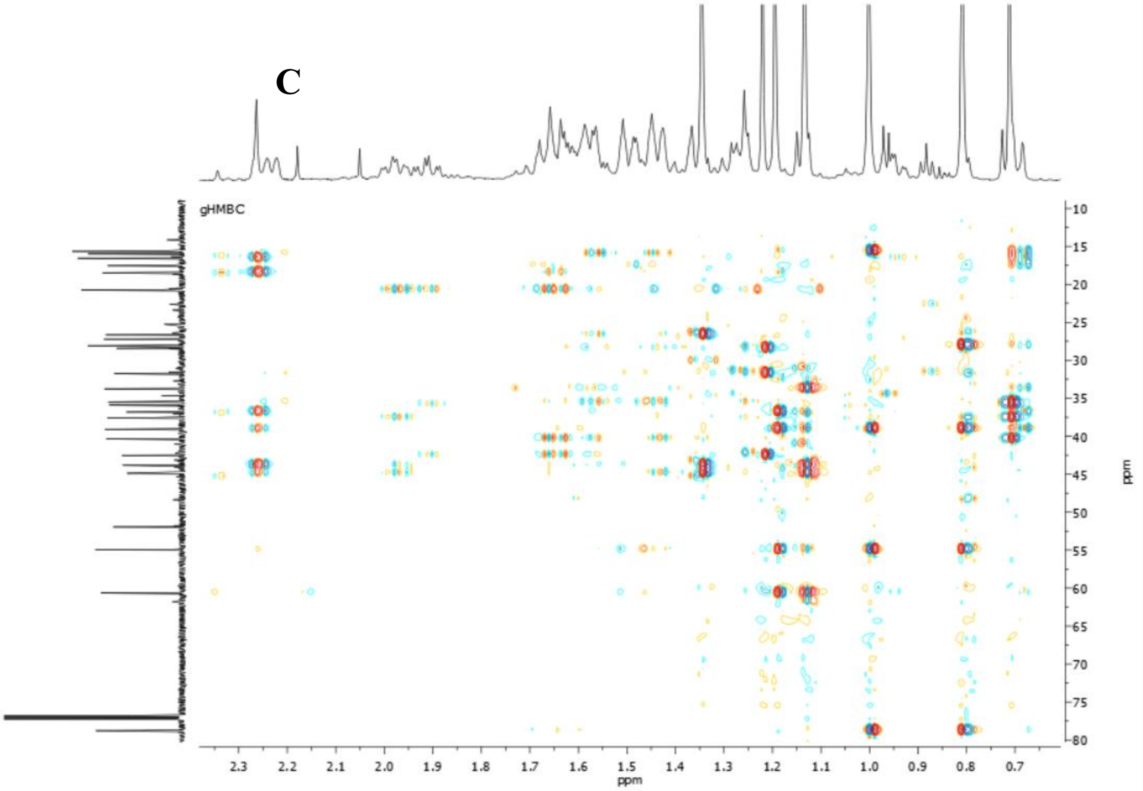

Figure S4. A) Full HMBC spectra of methyl-18α-3β-hydroxy-olean-12-en-30-oate. **B)** and **C)** Highfield spectra.

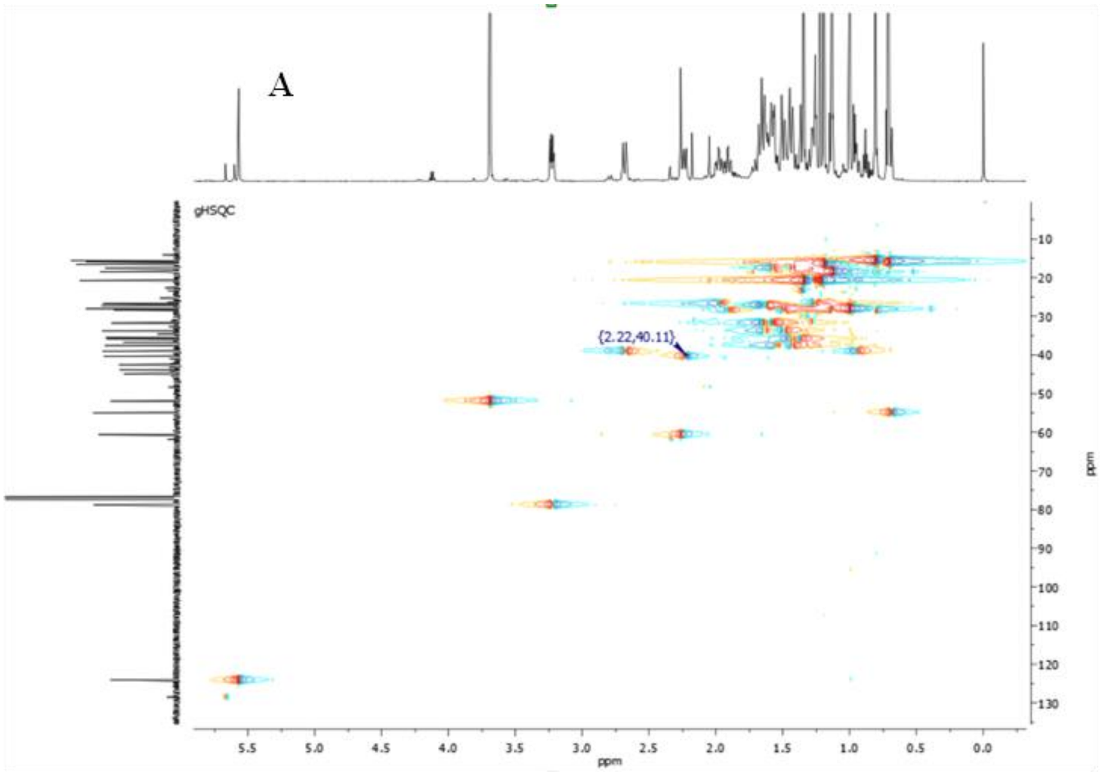

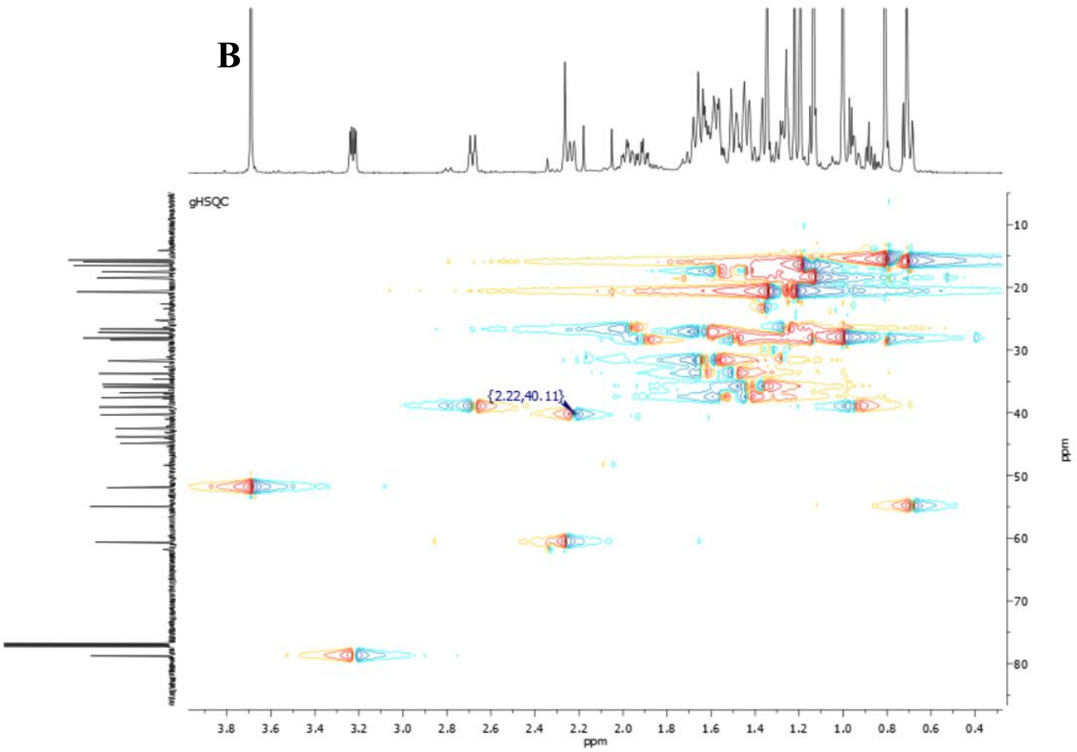

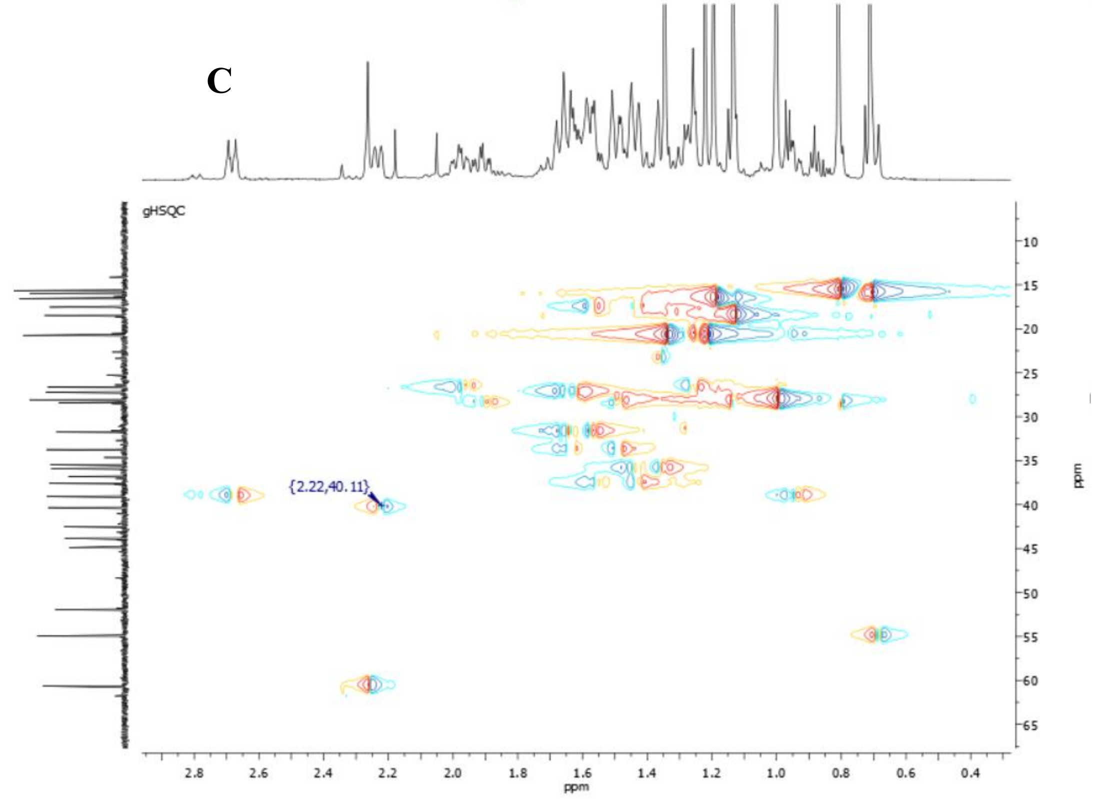

Figure S5. A) Full HSQC spectra of methyl-18α-3β-hydroxy-olean-12-en-30-oate. **B)**. and **C)** Highfield spectra.

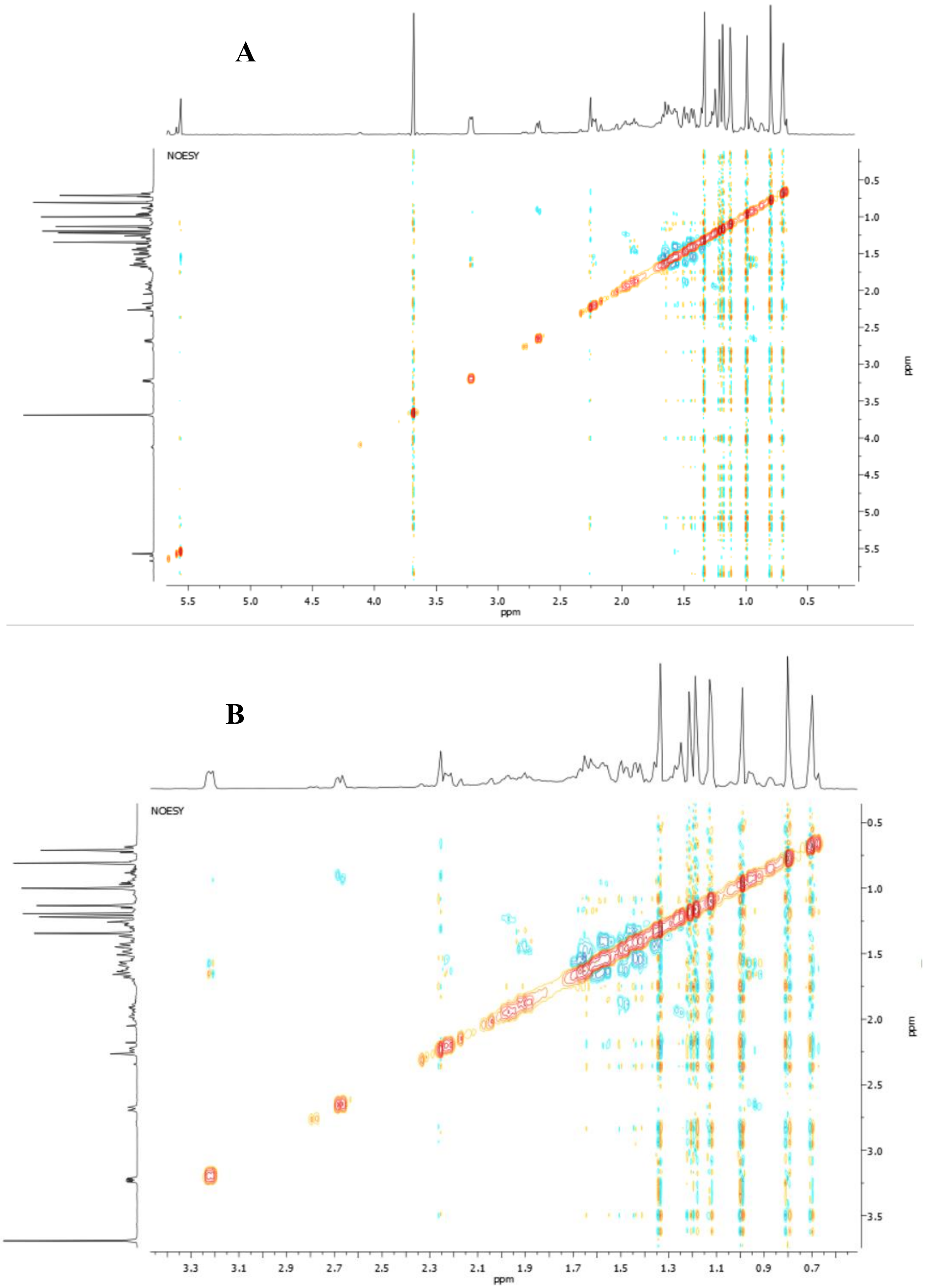

Figure S6. A) Full NOESY spectra of methyl-18α-3β-hydroxy-olean-12-en-30-oate. **B**) Highfield spectra.

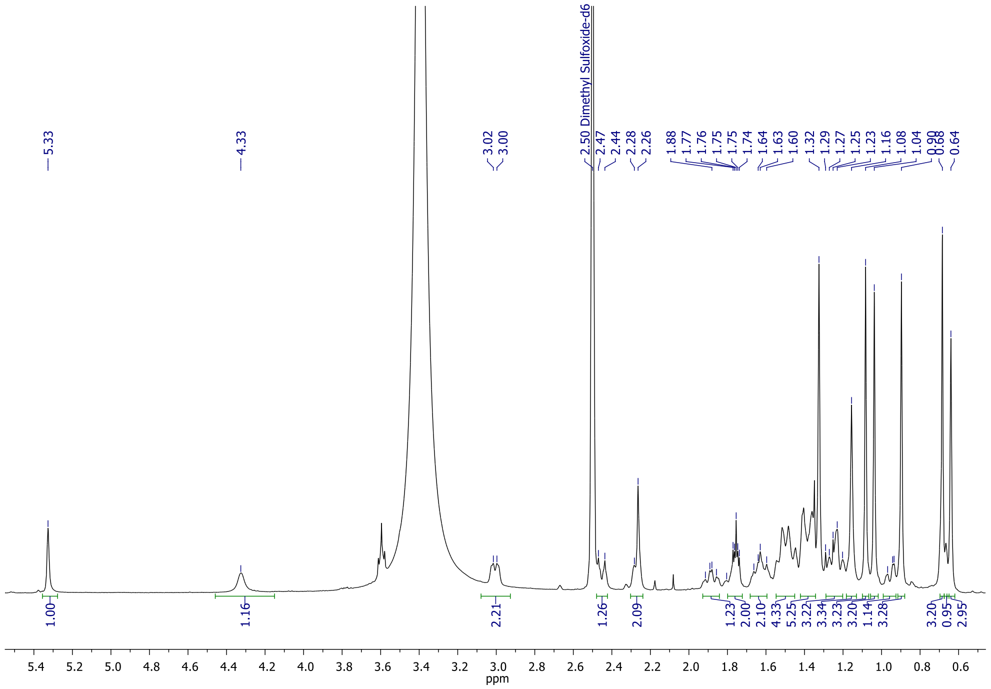

Figure S7. ^{1}H NMR (400 MHz) spectrum of **18α-GA** in DMSO-d6

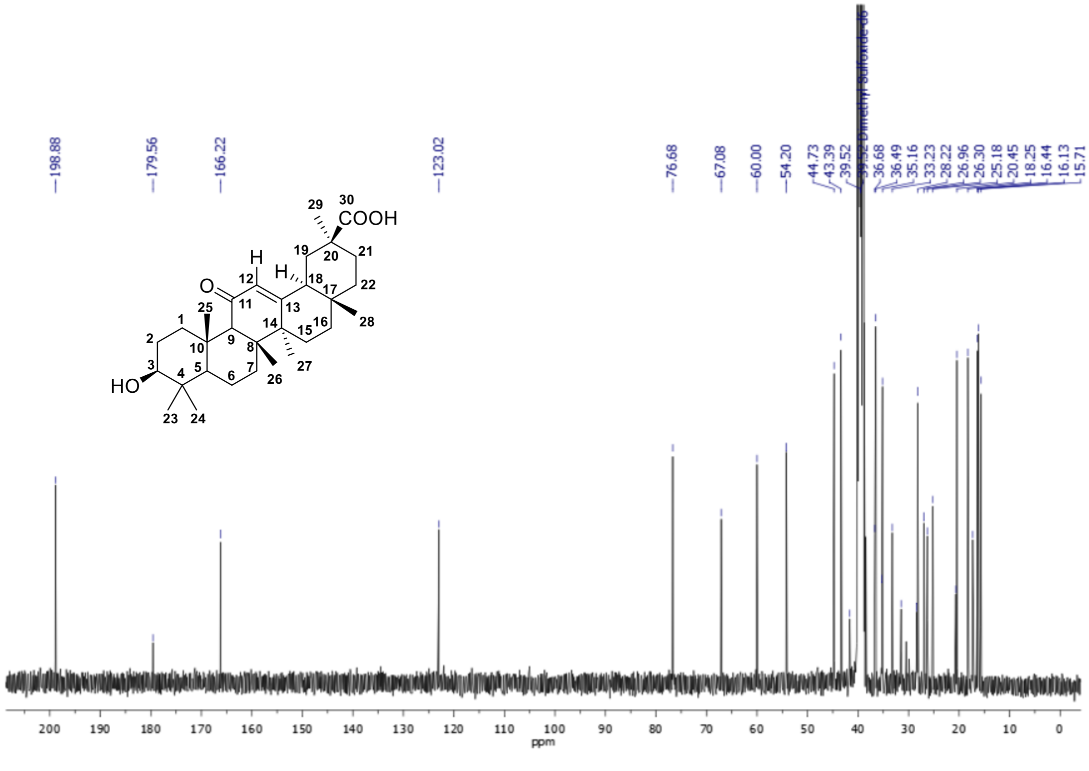

Figure S8. ^{13}C NMR (101 MHz) spectrum of **18α-GA** in DMSO-d6

A

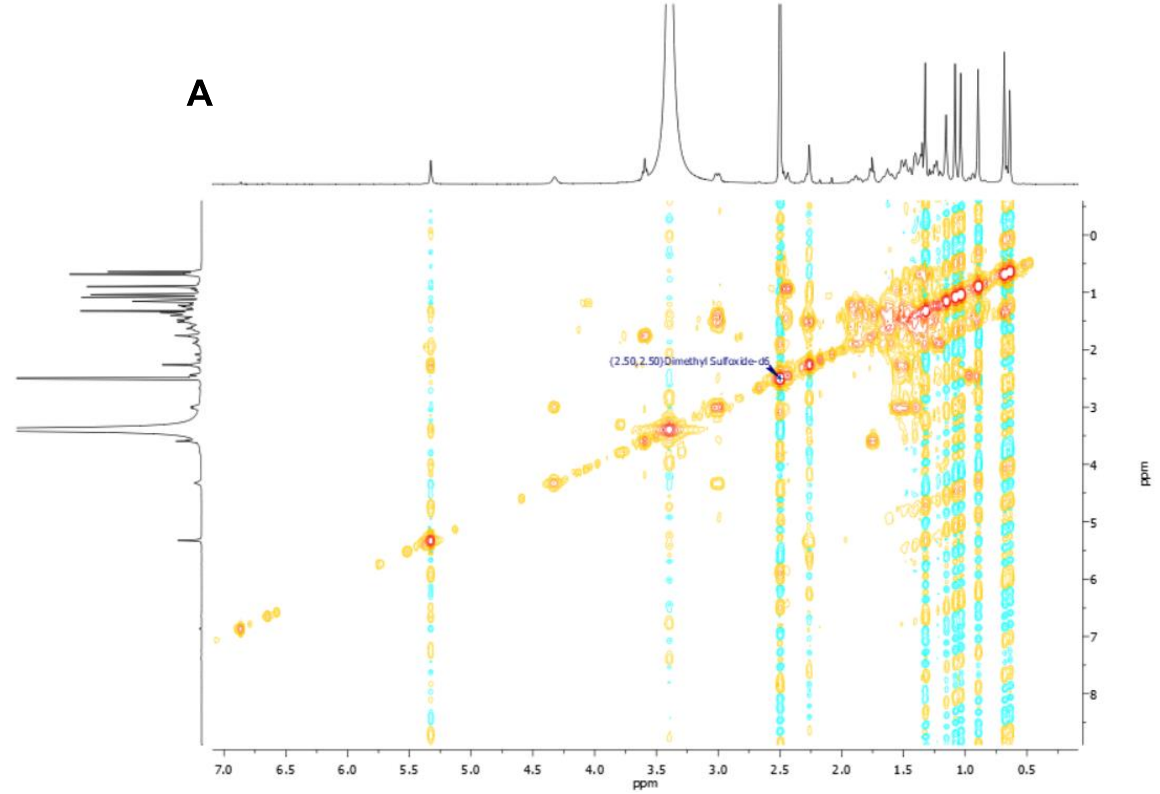
(2.50,2.50)Dimethyl Sulfoxide-d6
ppm
ppm
7.0
6.5
6.0
5.5
5.0
4.5
4.0
3.5
3.0
2.5
2.0
1.5
1.0
0.5

B

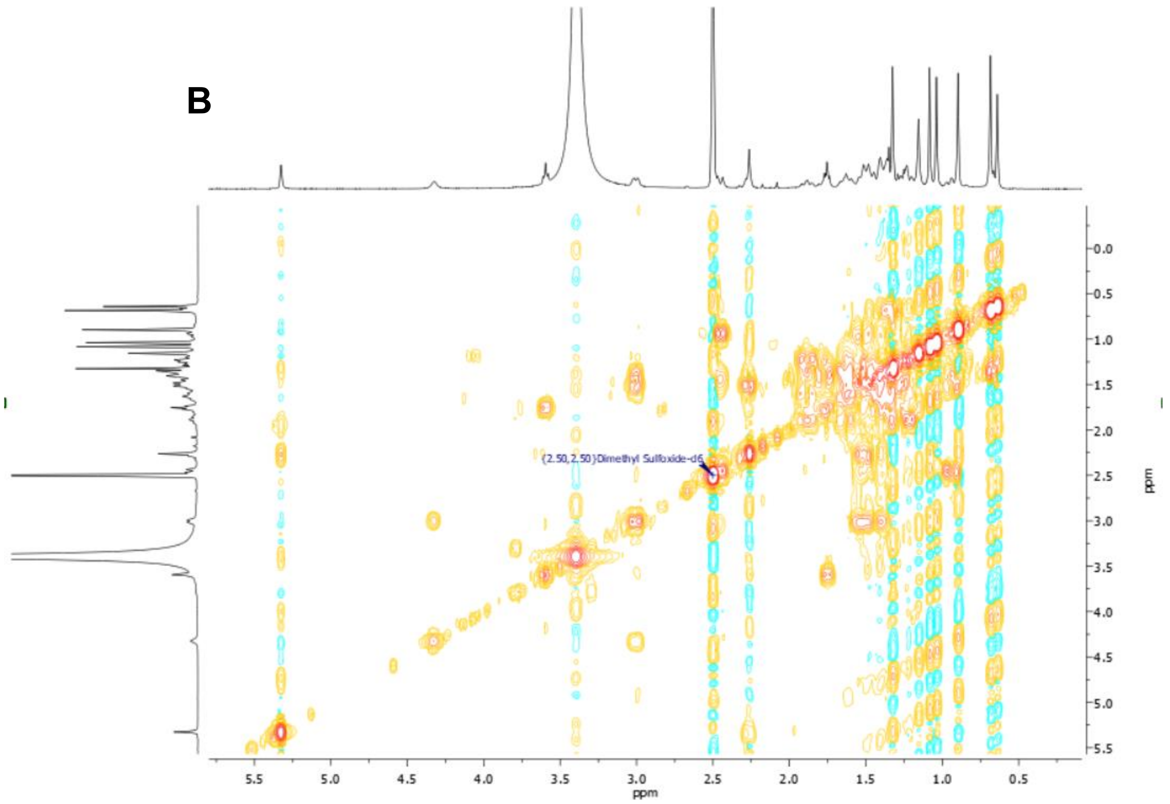
(2.50,2.50)Dimethyl Sulfoxide-d6
ppm
ppm
5.5
5.0
4.5
4.0
3.5
3.0
2.5
2.0
1.5
1.0
0.5

C

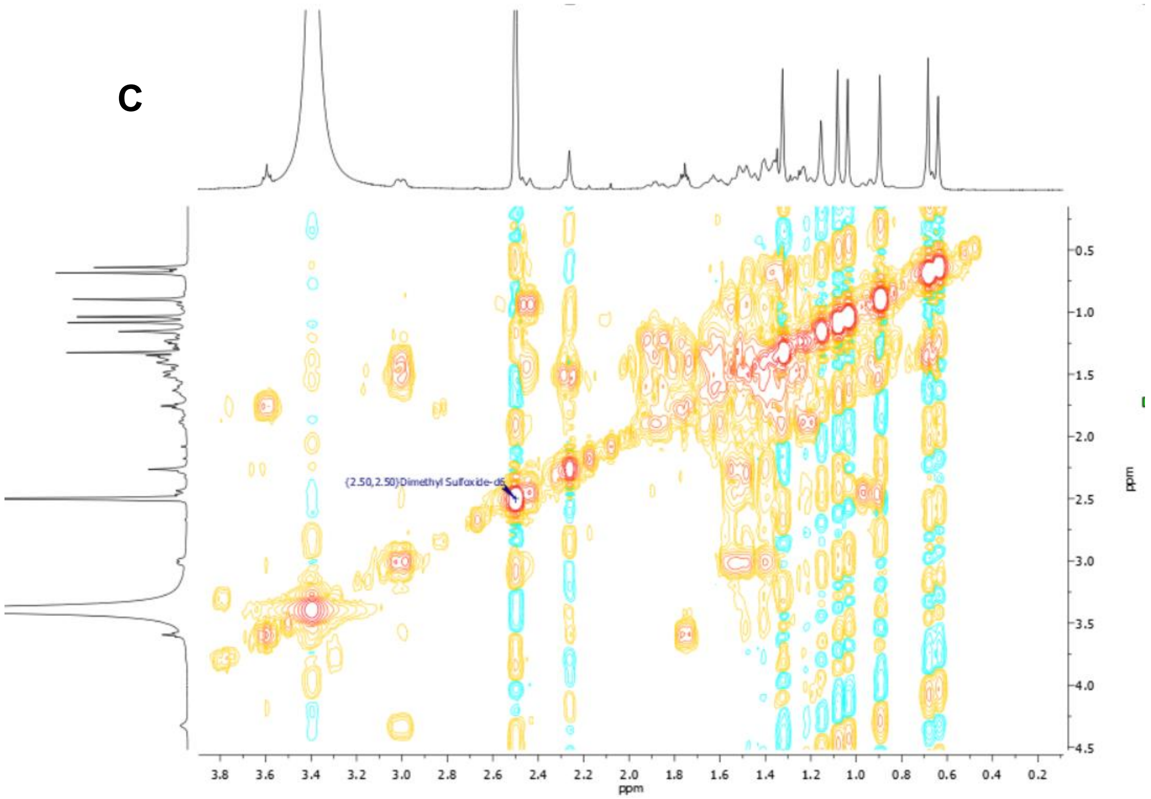

Figure S9. A) Full COSY spectra of **18α-GA** in DMSO-d6. **B)** and **C)** Highfield spectra.

A

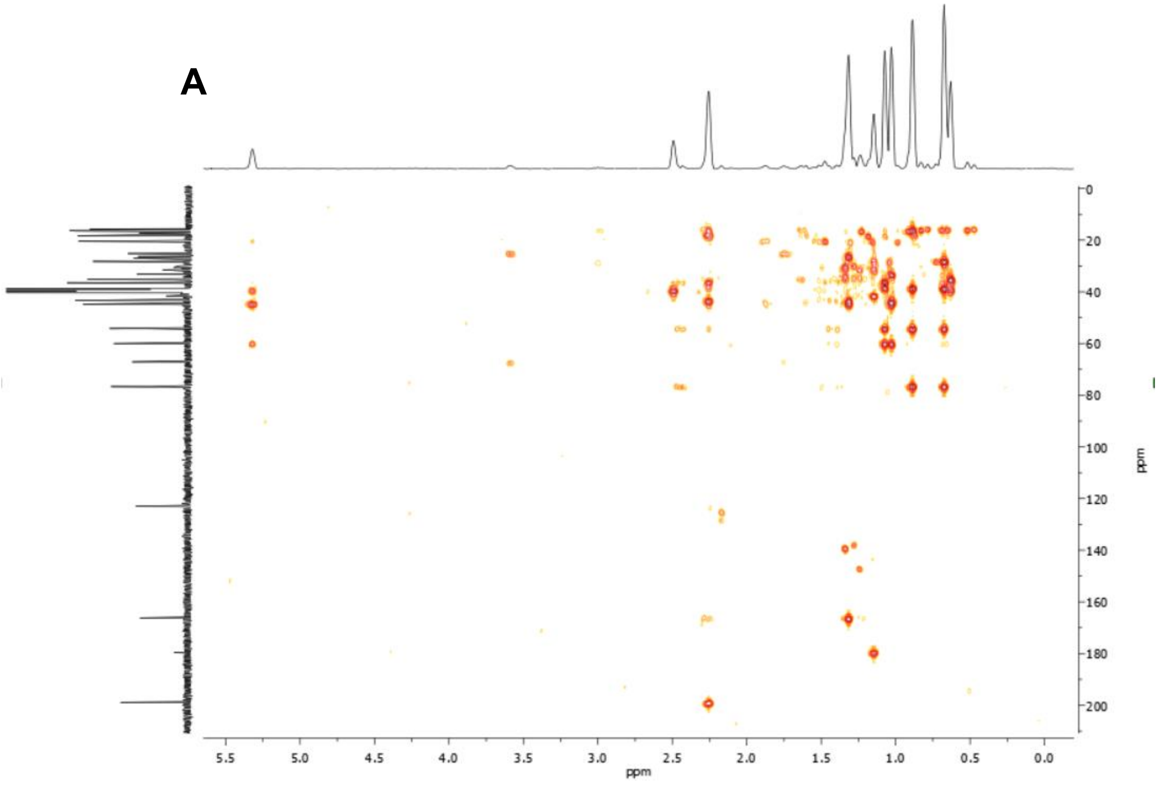

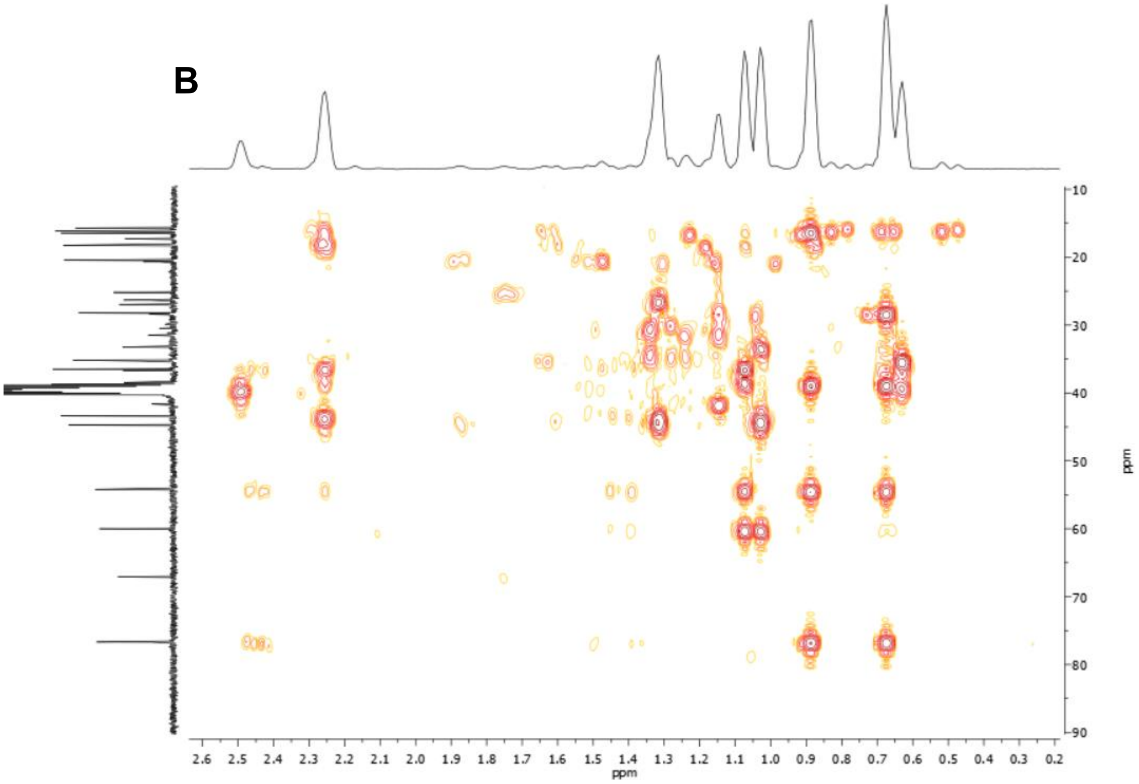

Figure S10. A) Full HMBC spectra of **18α-GA** in DMSO-d6. **A**) and **B)** Highfield spectra.

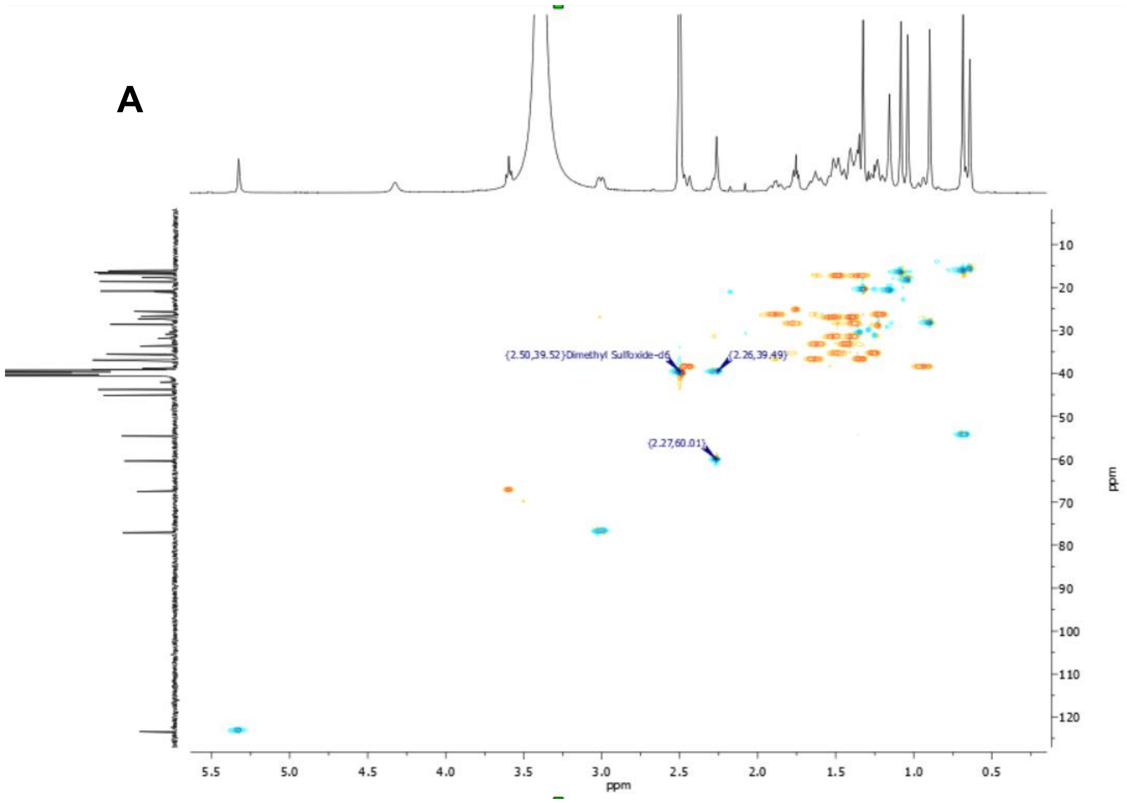

B

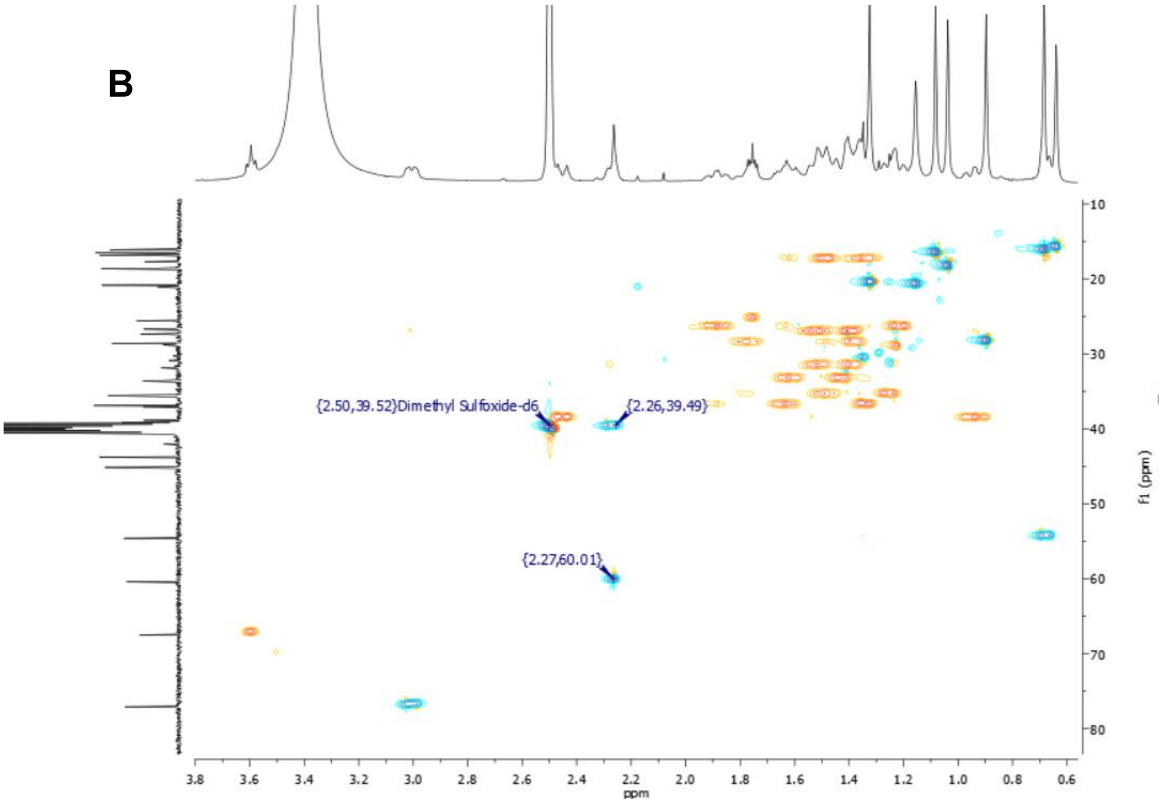

C

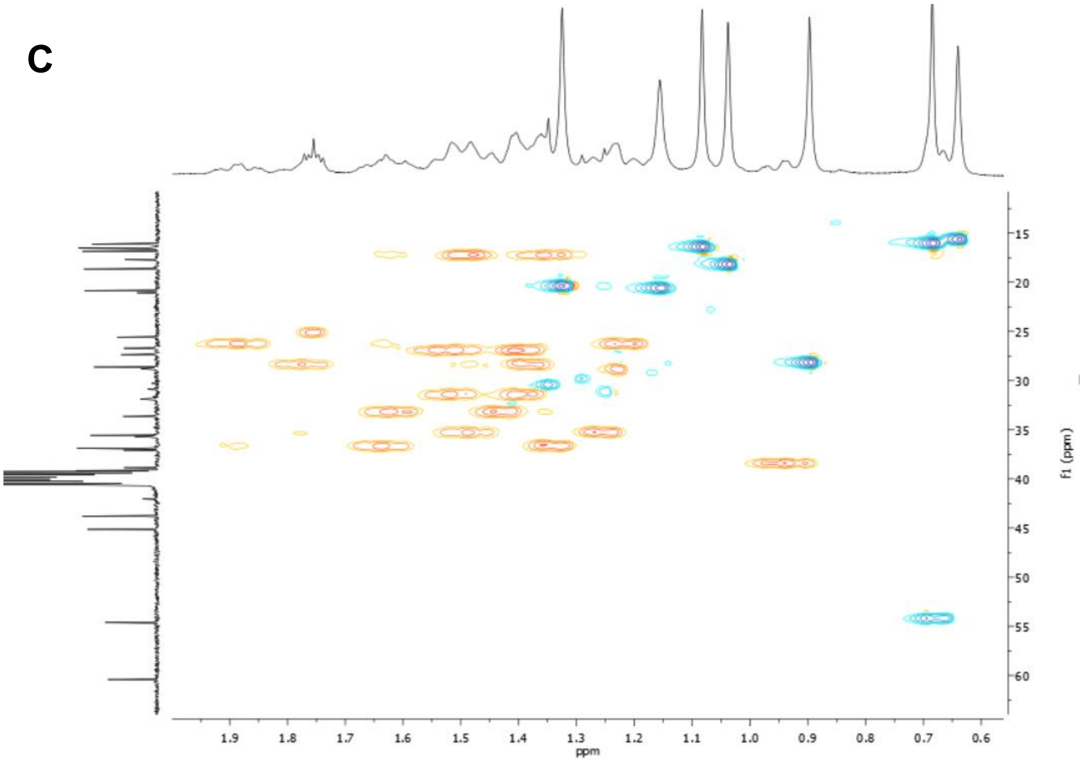

Figure S11. A) Full HSQC spectra of **18α-GA** in DMSO-d6. **B)** and **C)** Highfield spectra.

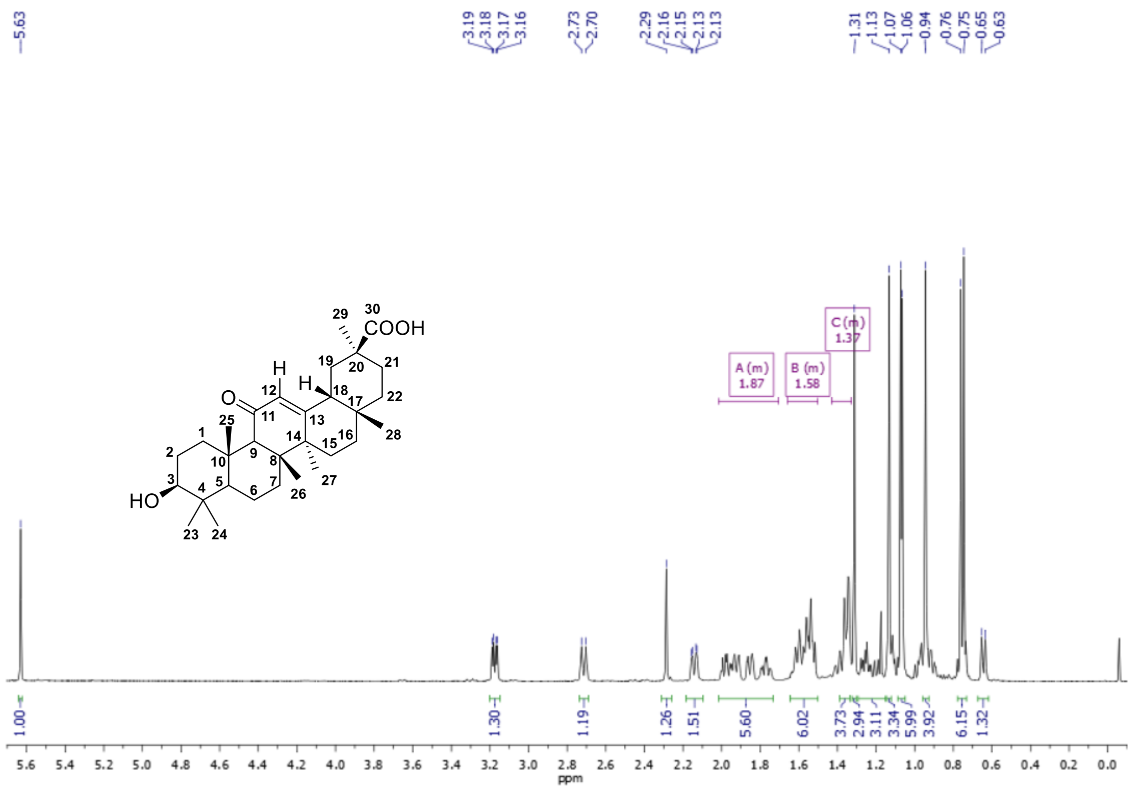

Figure S12. ^{1}H NMR (600 MHz) spectrum of **18β-GA** in $CDCl_3$

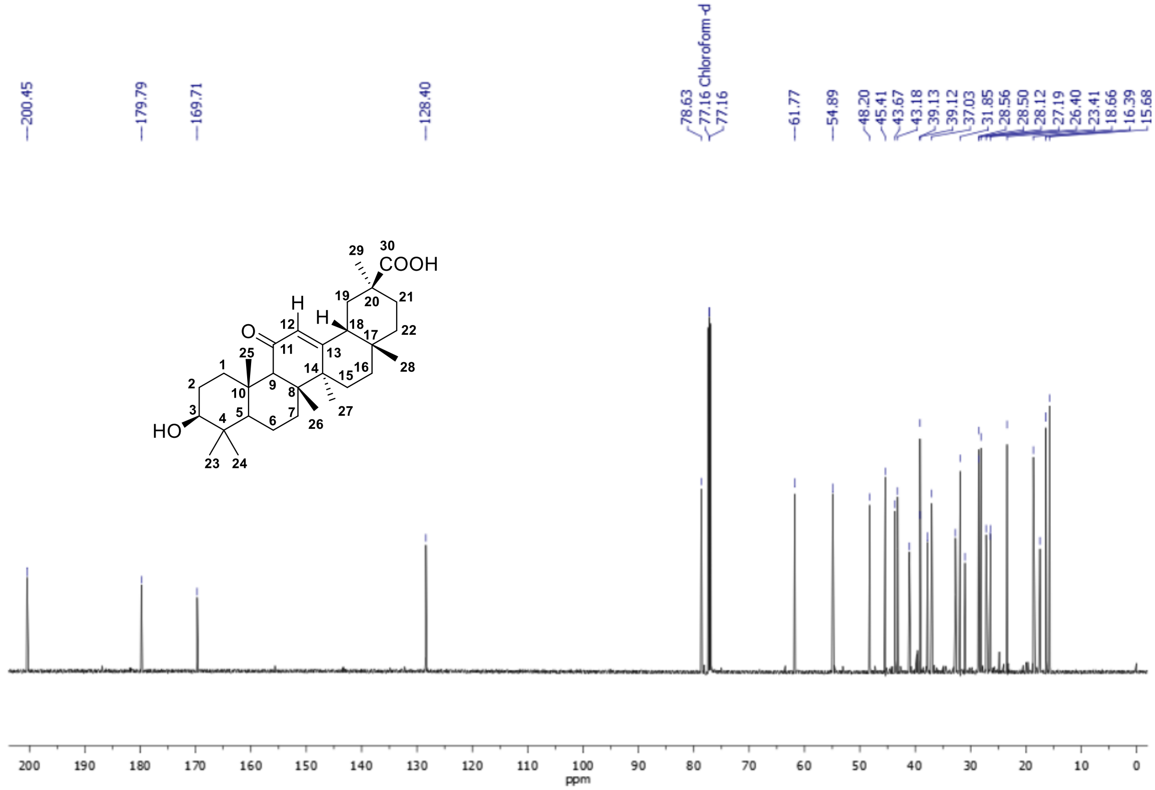

Figure S13. ^{13}C NMR (151 MHz) spectrum of **18β-GA** in $CDCl_3$

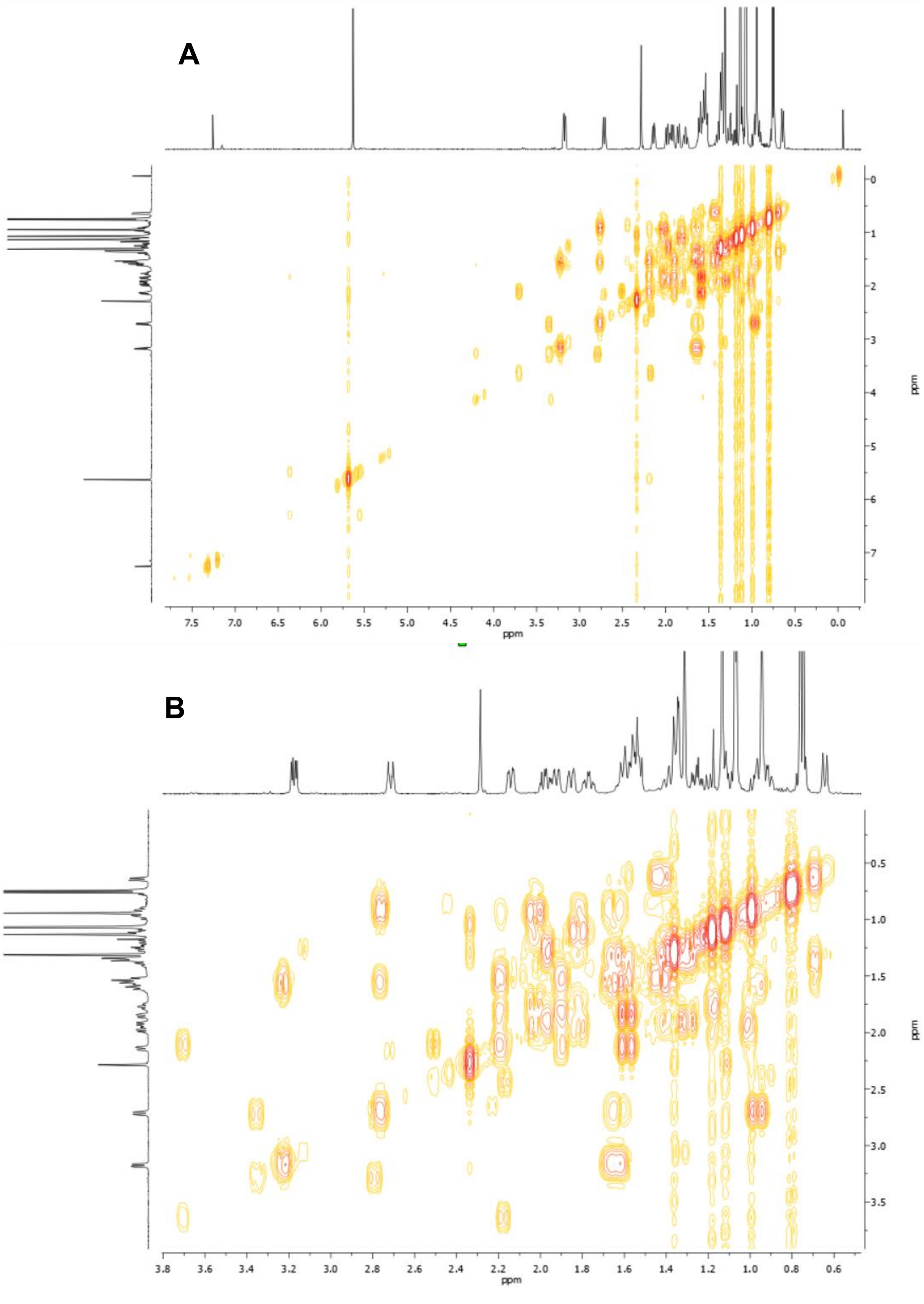

Figure S14. A) Full COSY spectra of **18β-GA** in $CDCl_3$. **B)**. Highfield spectra.

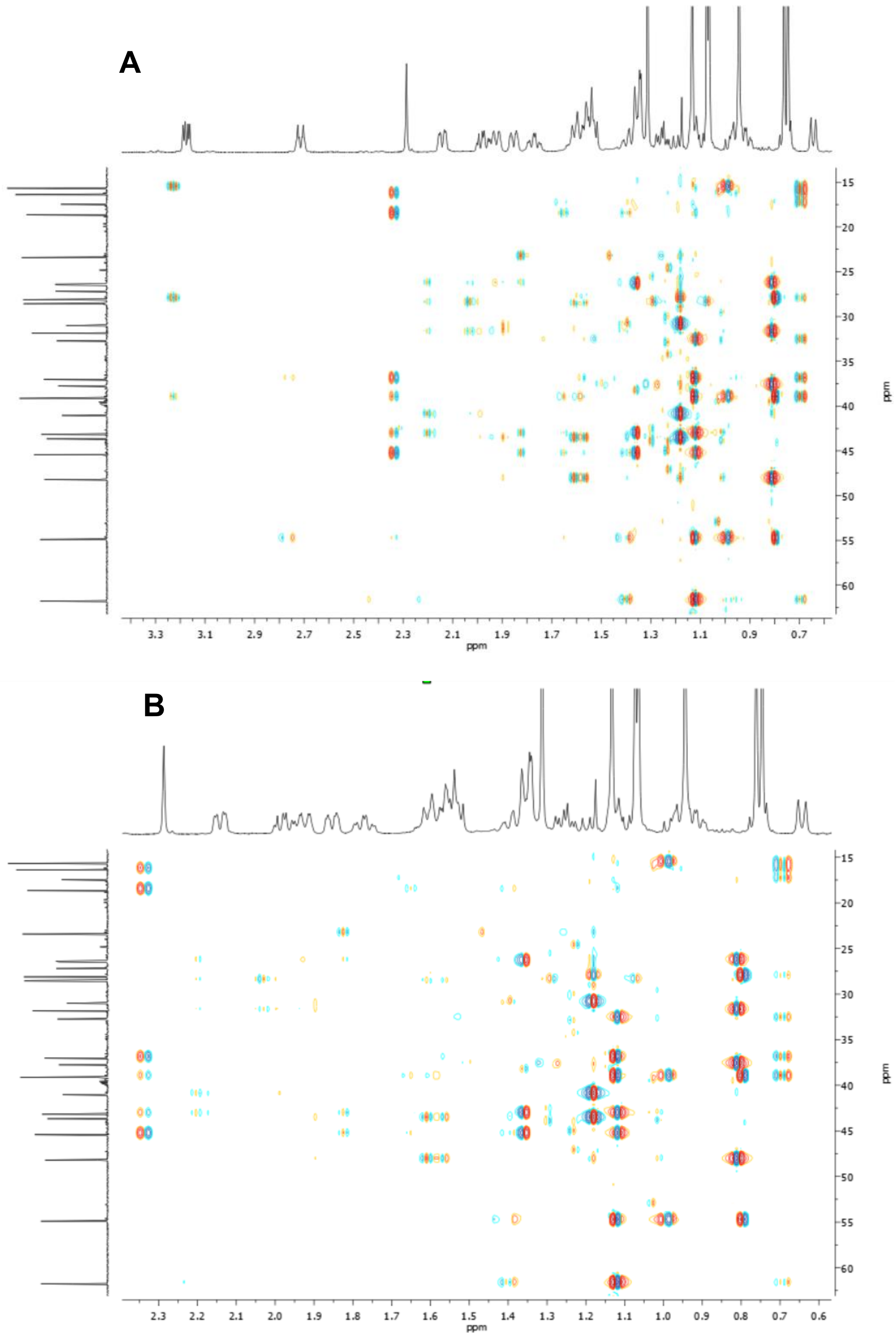

Figure S15. A) Full HMBC spectra of **18β-GA** in $CDCl_3$. **B)** Highfield spectra.

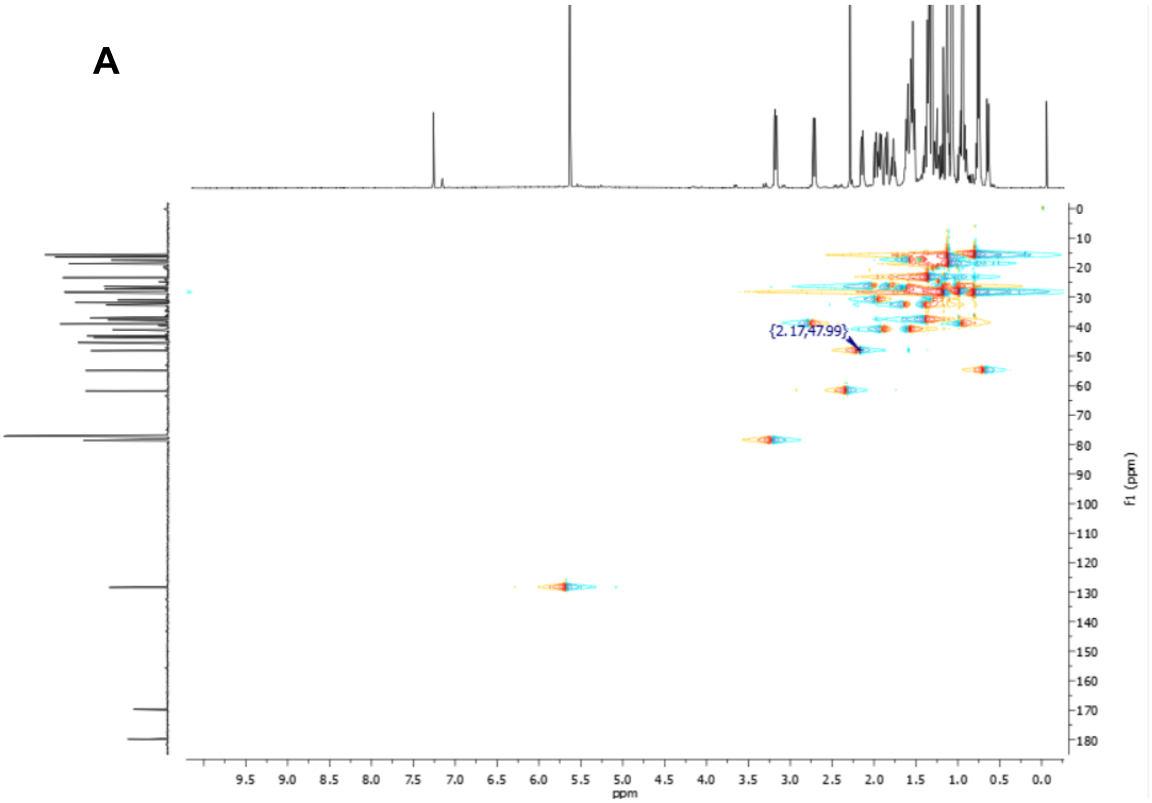

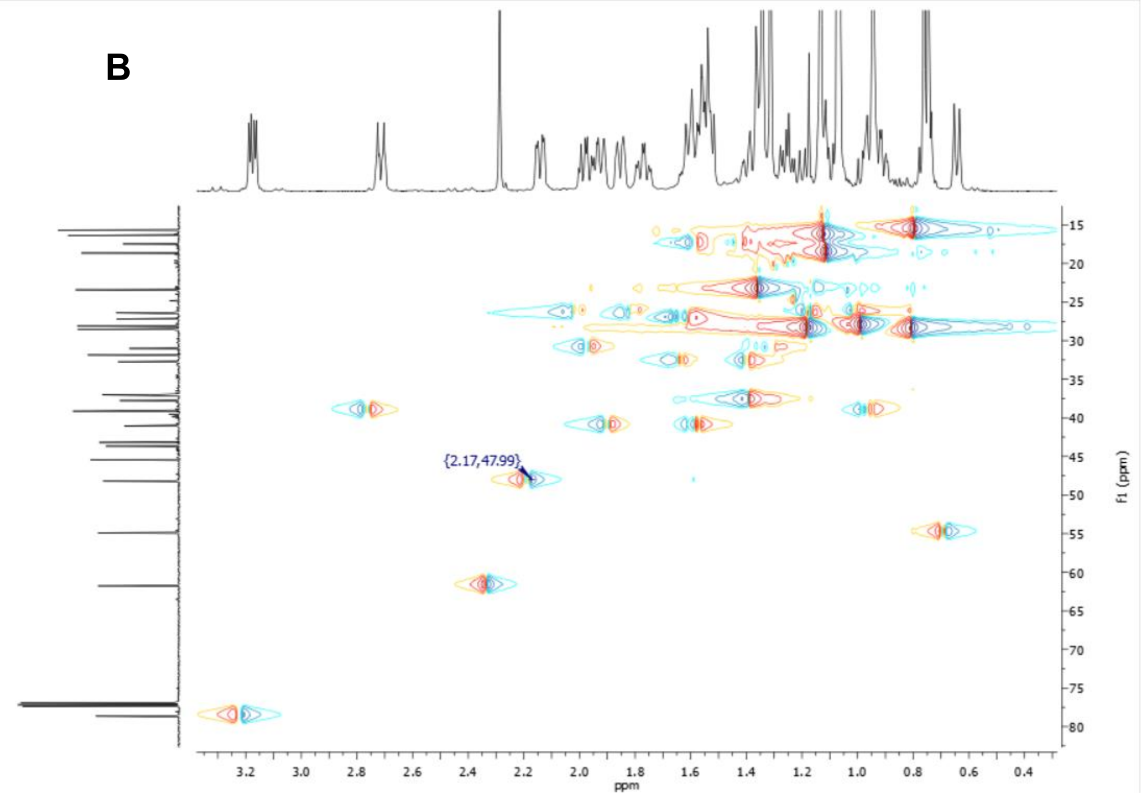

Figure S16. A) Full HSQC spectra of **18β-GA** in $CDCl_3$. **B)** Highfield spectra.

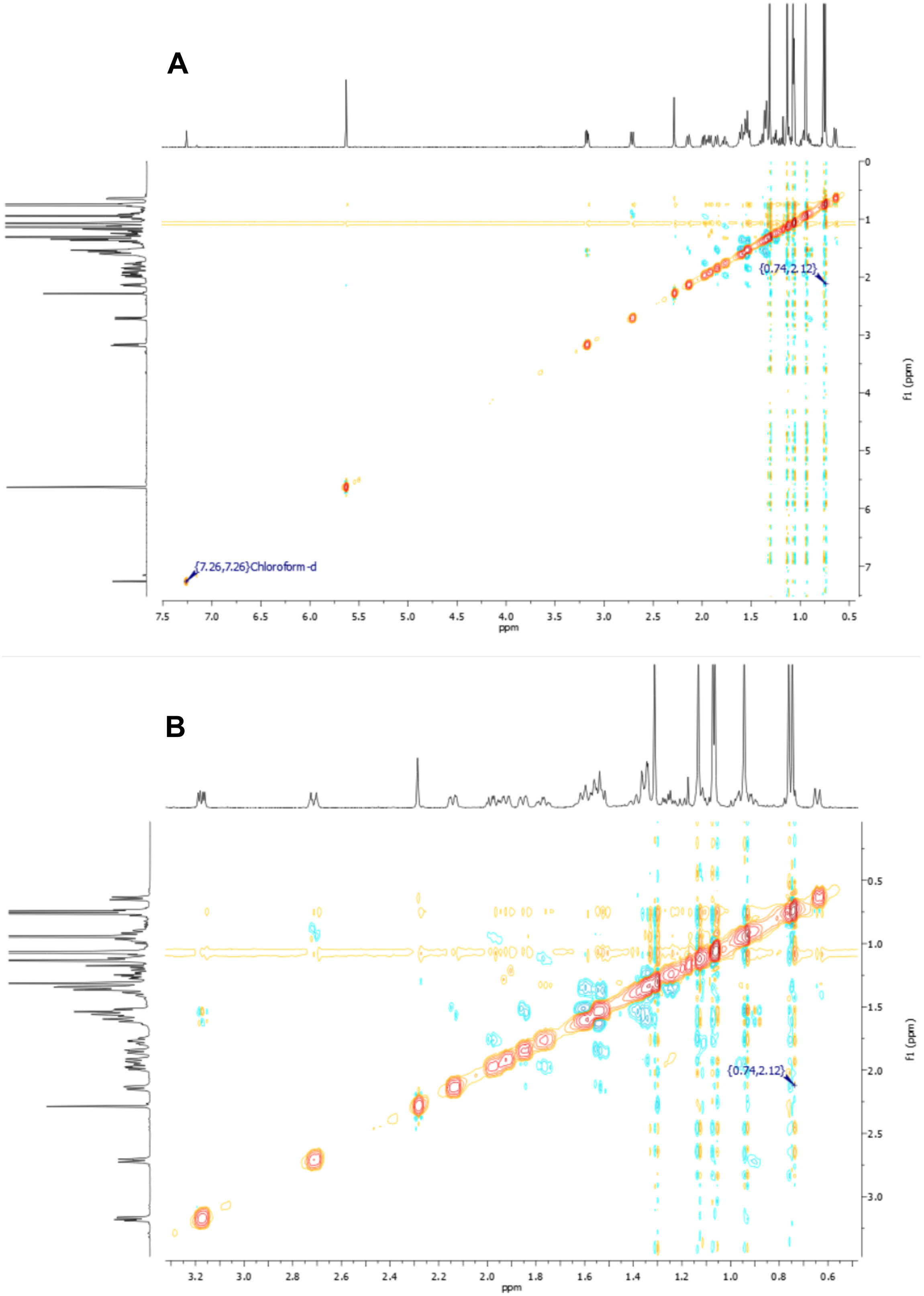

Figure S17. A) Full NOESY spectra of **18β-GA** in $CDCl_3$. **B**) Highfield spectra.

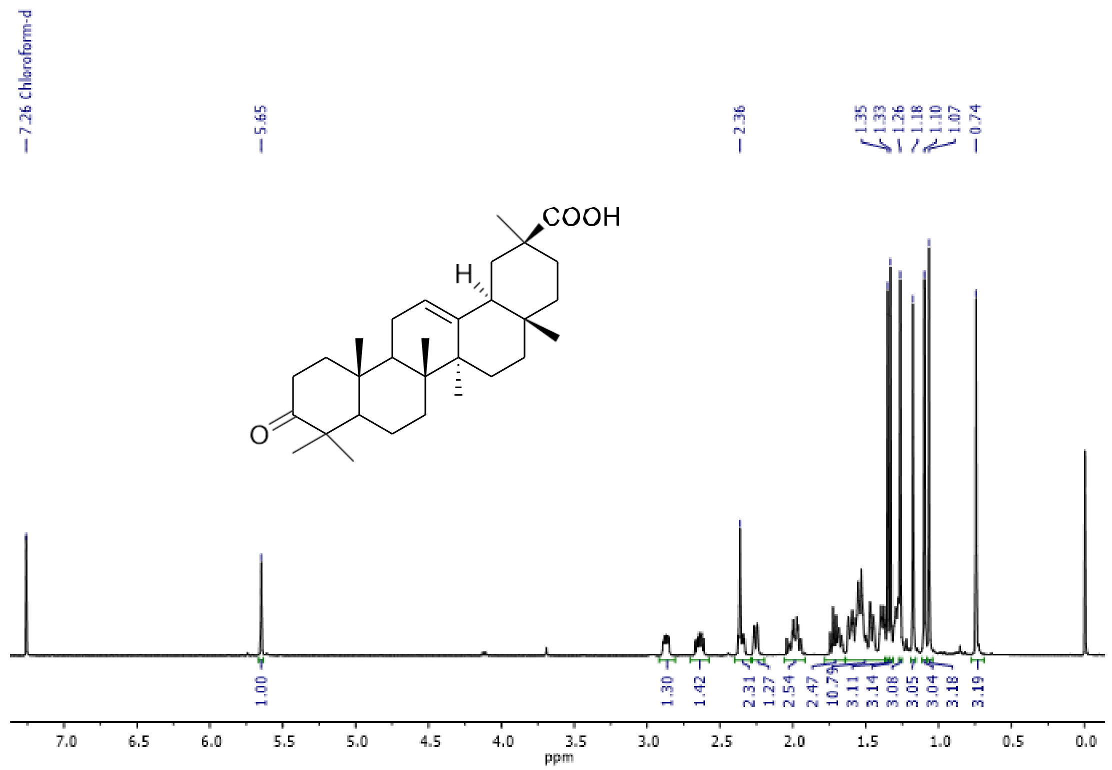

Figure S18. ^{1}H NMR (600 MHz) spectrum of compound **2a** in $CDCl_3$

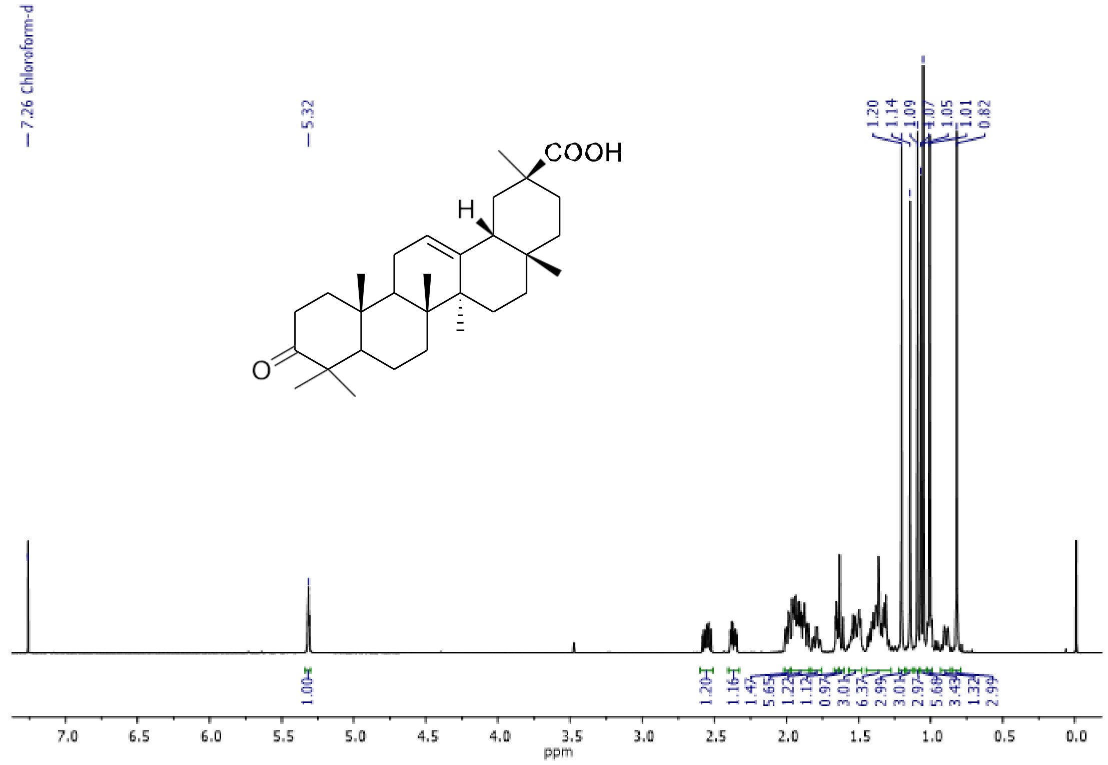

Figure S19. ^{1}H NMR (600 MHz) spectrum of compound **2b** in $CDCl_3$

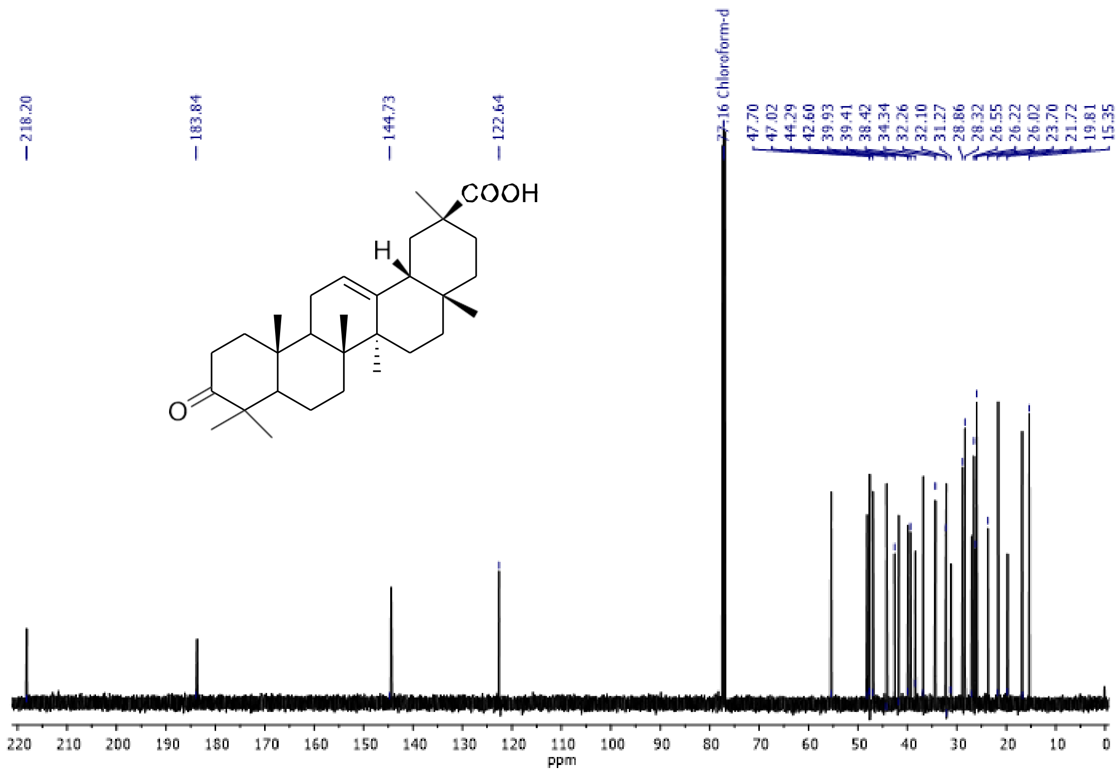

Figure S20. ^{13}C NMR (151 MHz) spectrum of compound **2b** in $CDCl_3$

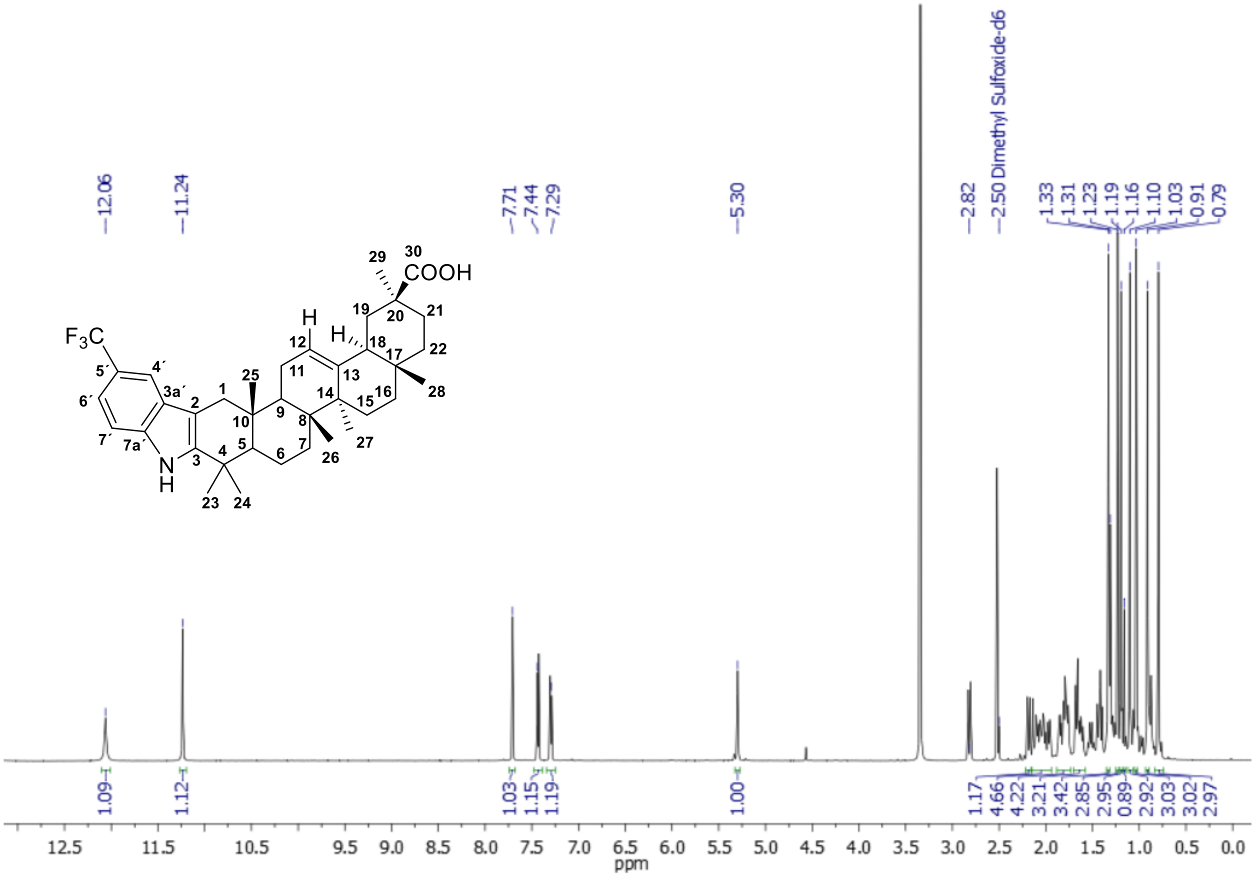

Figure S21. ^{1}H NMR (600 MHz) spectrum of compound **3a** in DMSO-d6

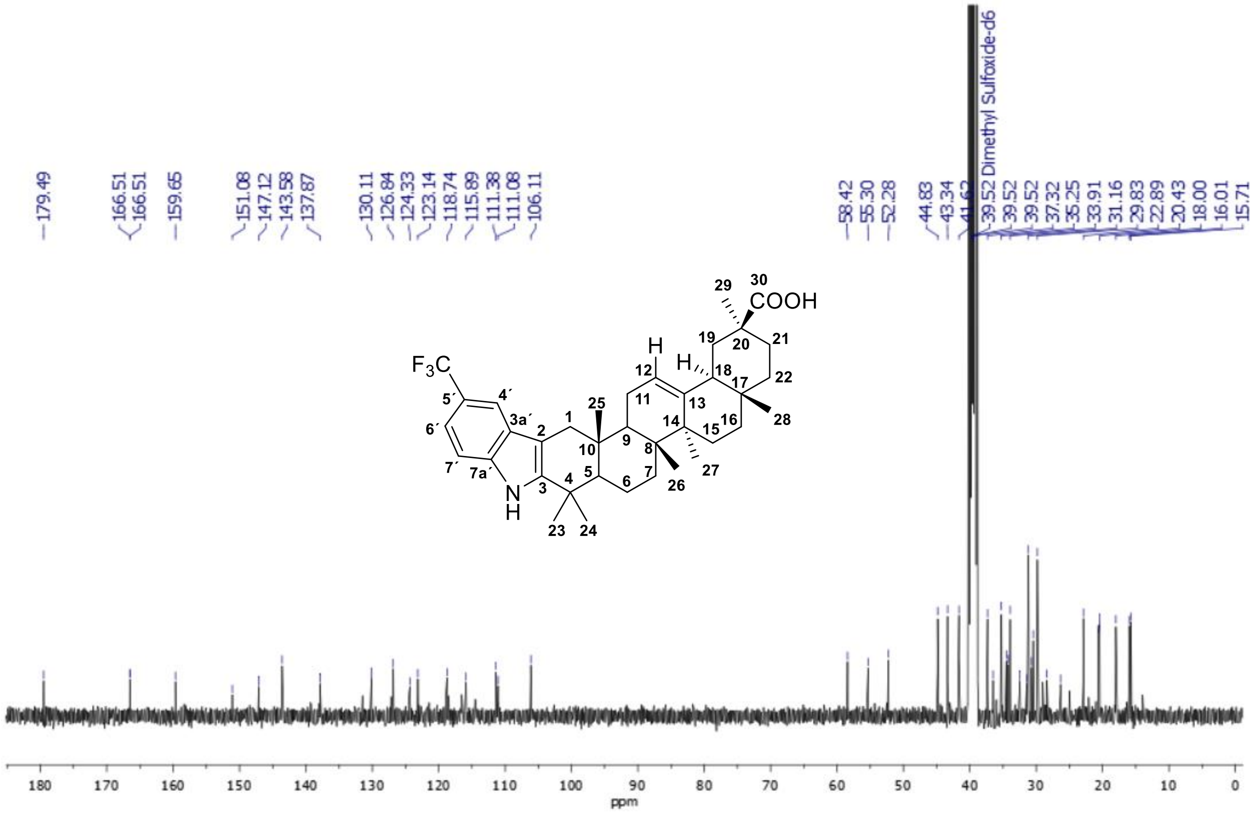

Figure S22. ^{13}C NMR (101 MHz) spectrum of compound **3a** in DMSO-d$_6$

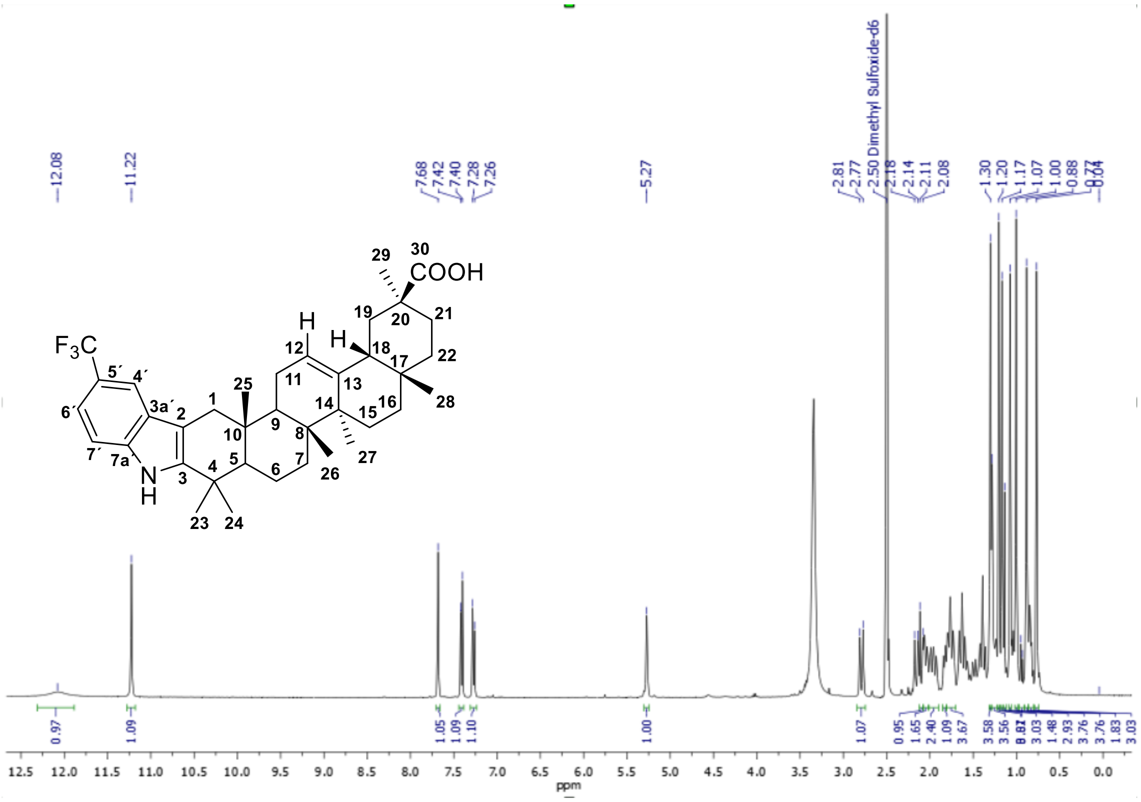

Figure S23. ^{1}H NMR (400 MHz) spectrum of compound **3b** in DMSO-d$_6$

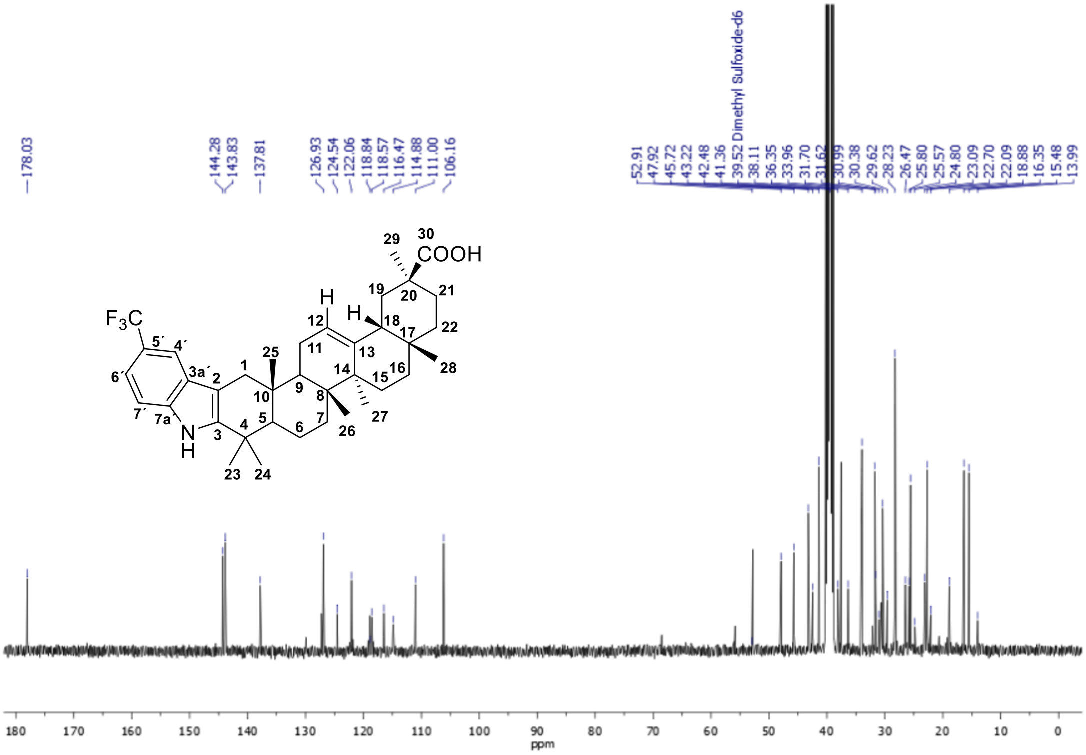

Figure S24. ^{13}C NMR (151 MHz) spectrum of compound **3b** in DMSO-d_6

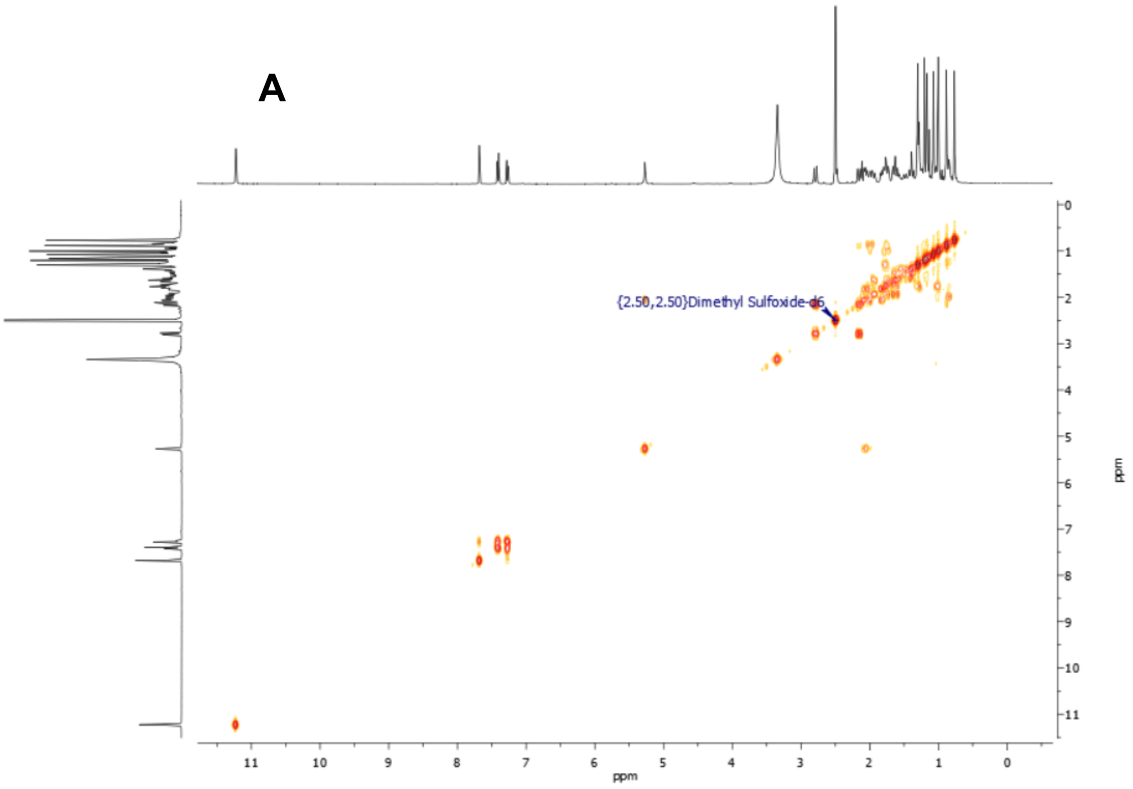

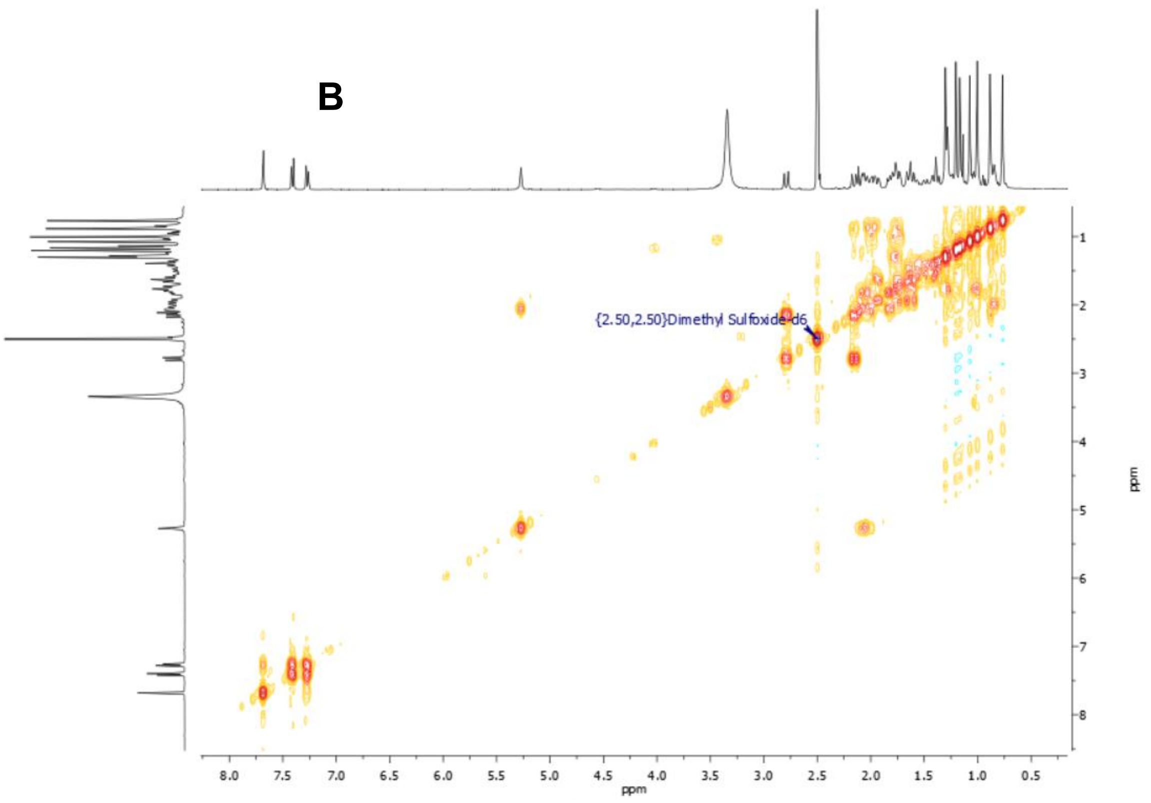
B
{2.50,2.50}Dimethyl Sulfoxide-d6
ppm
ppm

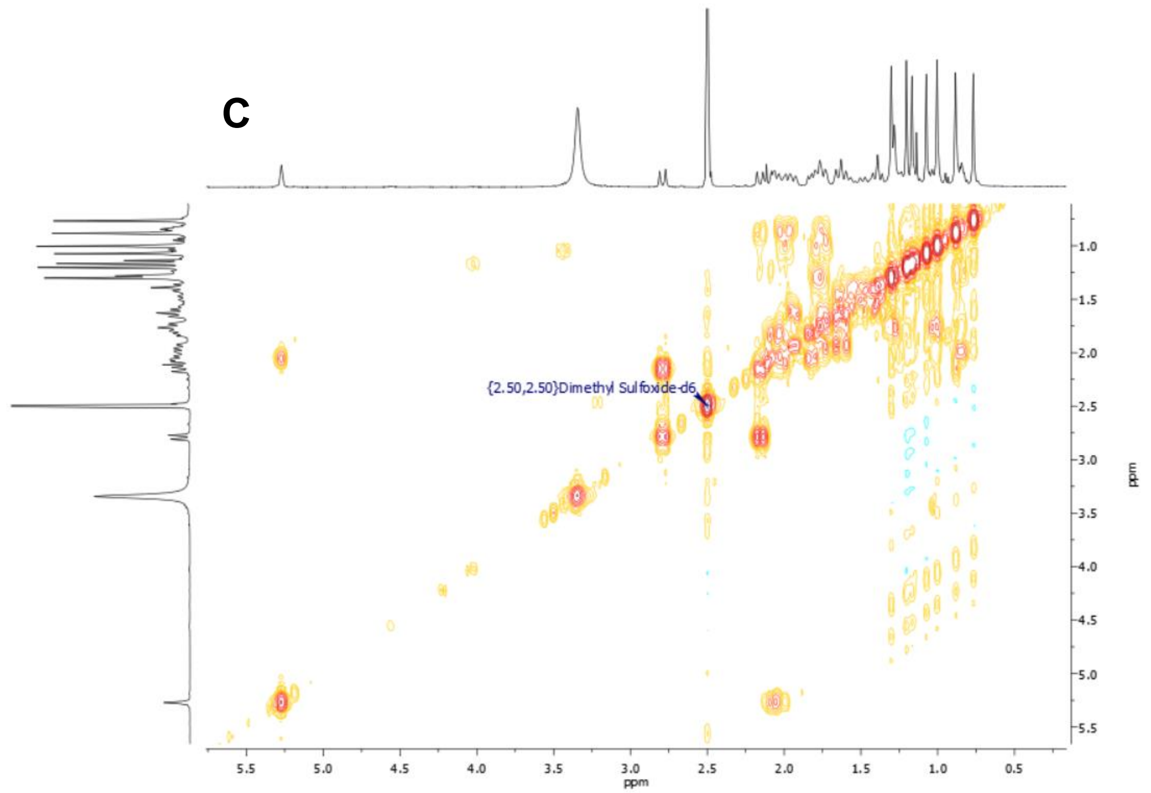
C
{2.50,2.50}Dimethyl Sulfoxide-d6
ppm
ppm

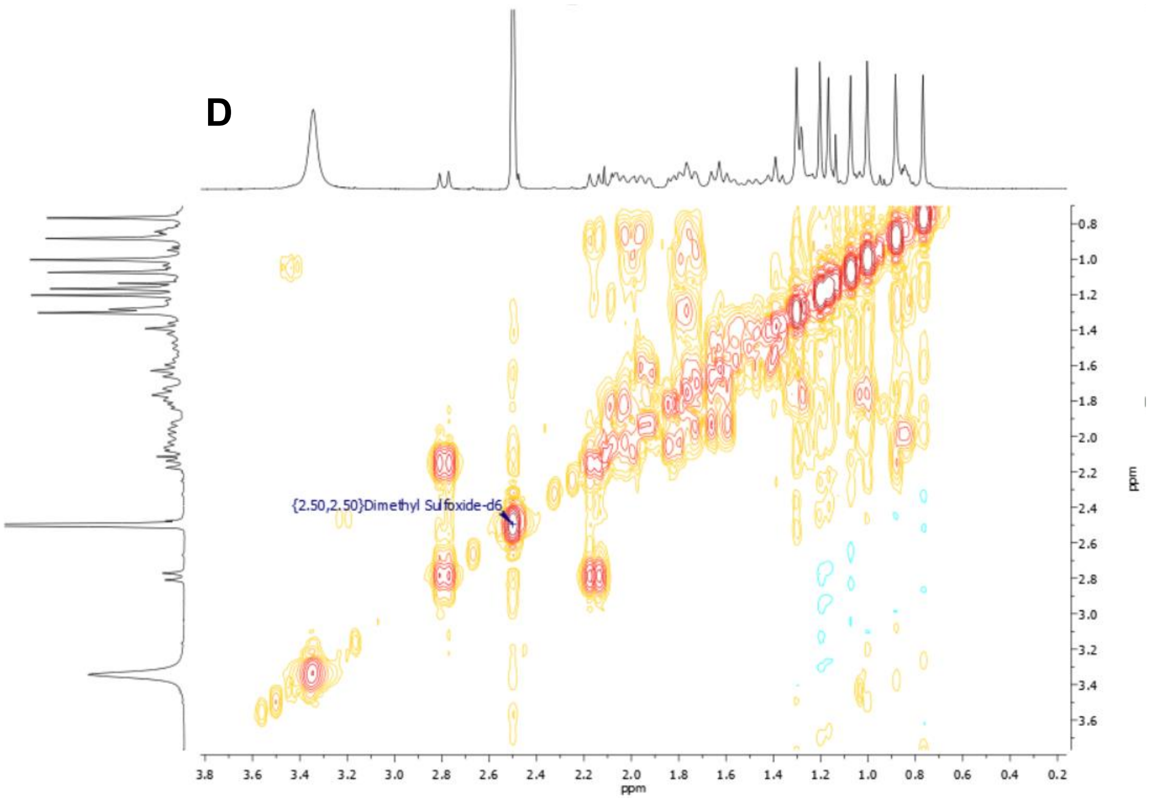

Figure S25. A) Full COSY spectra of **3b** in DMSO-d_6. **B), C)** and **D)** Highfield spectra.

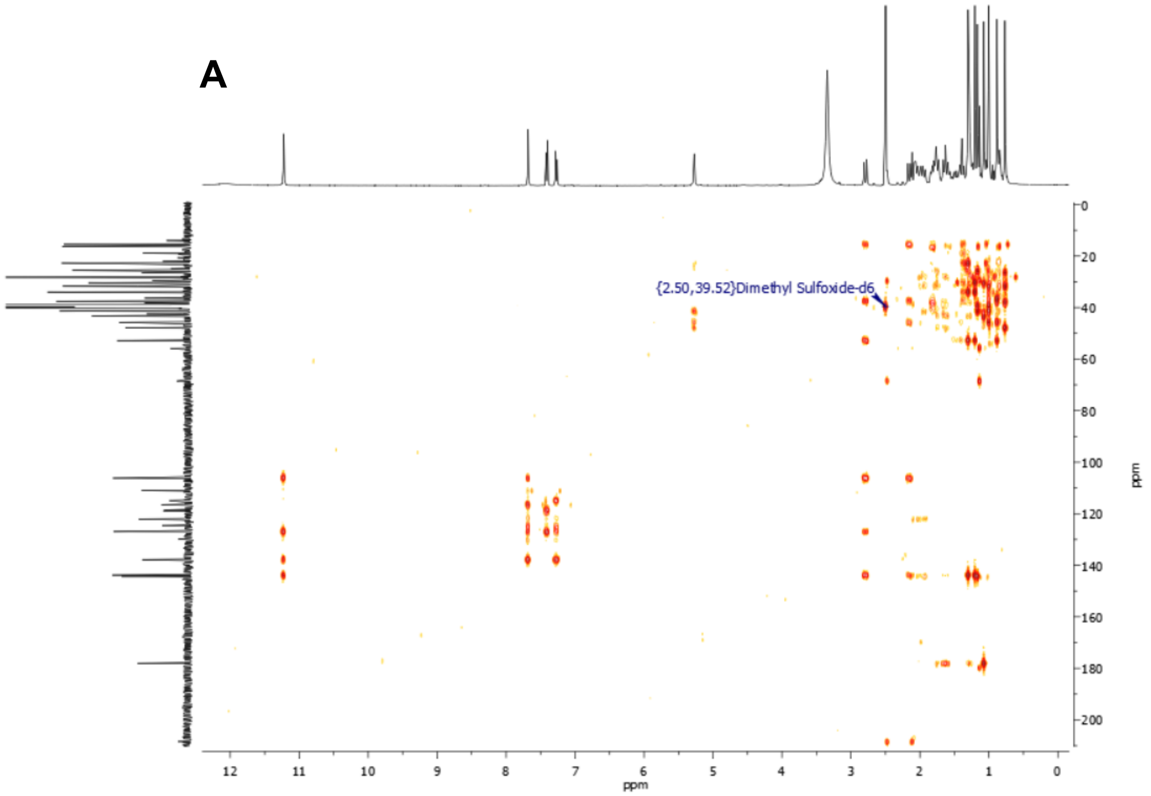

B

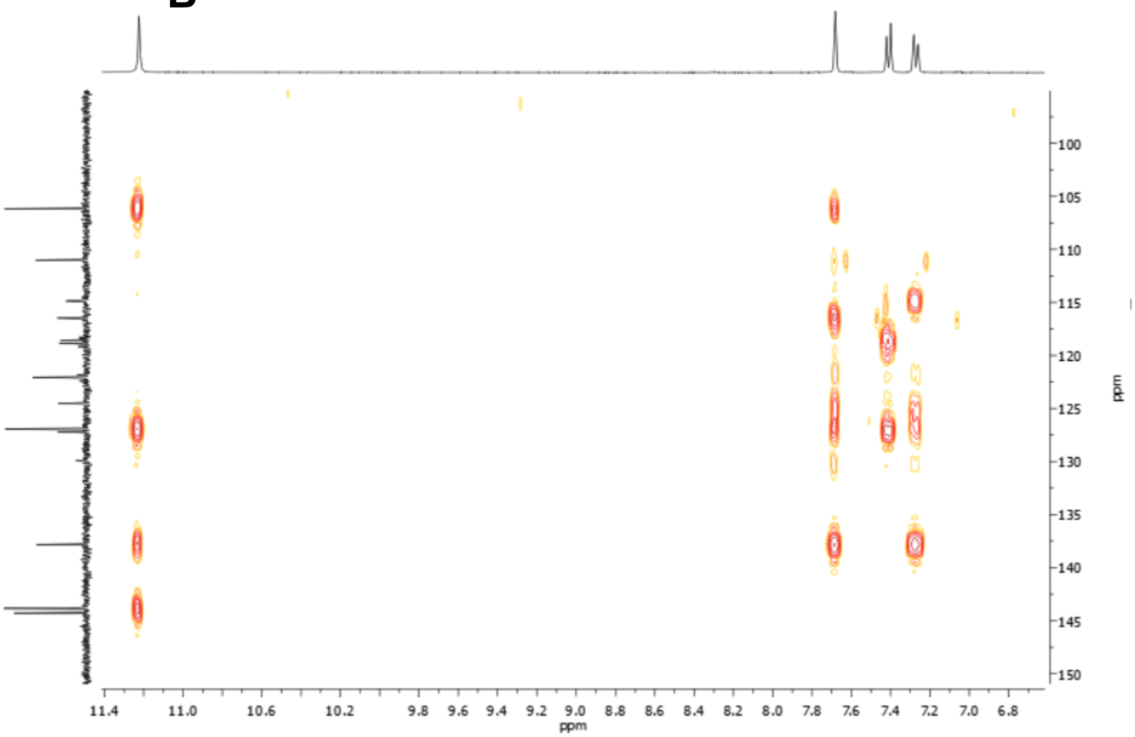

C

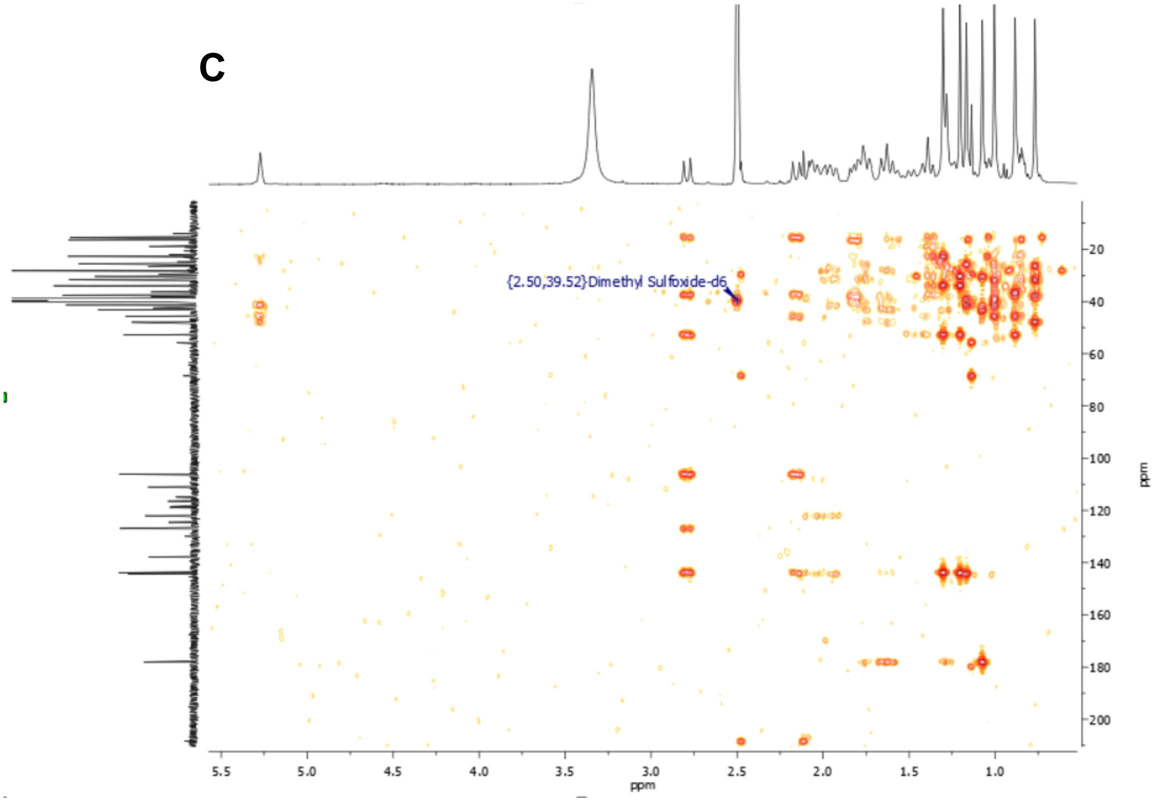

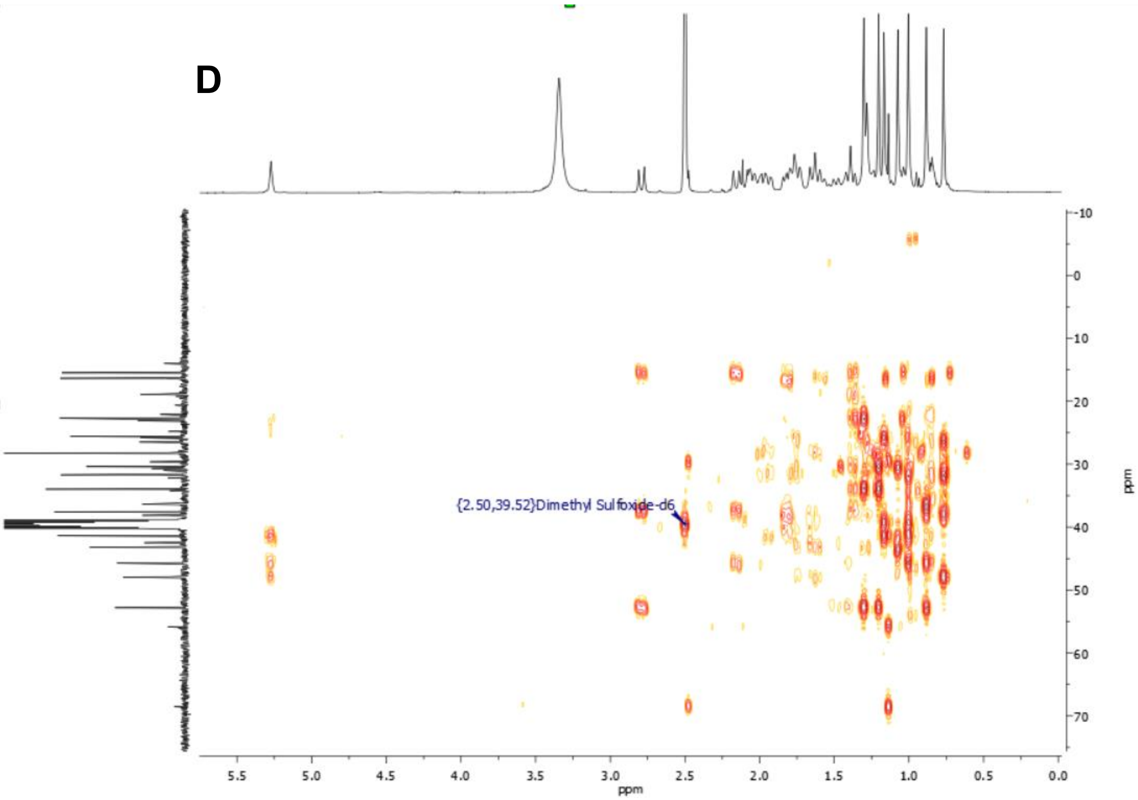

Figure S26. A) Full HMBC spectra of **3b** in DMSO-d6**. B), C)** and **D**) Highfield spectra.

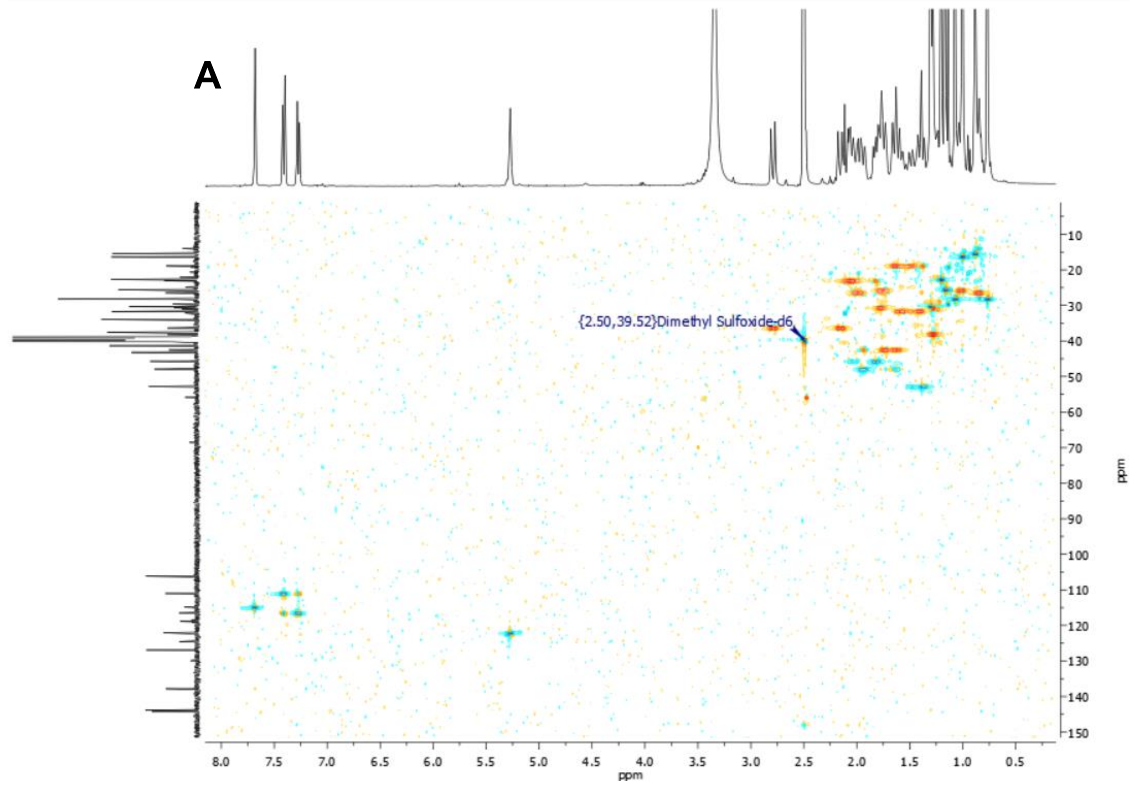

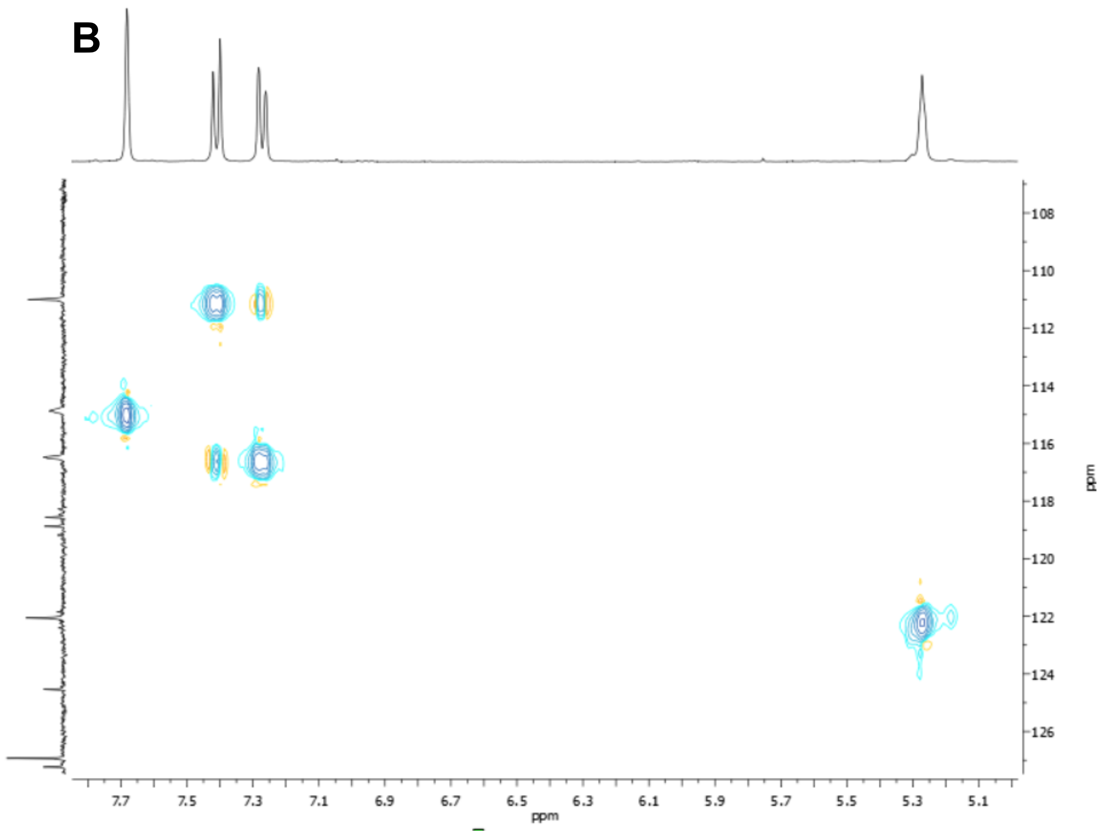

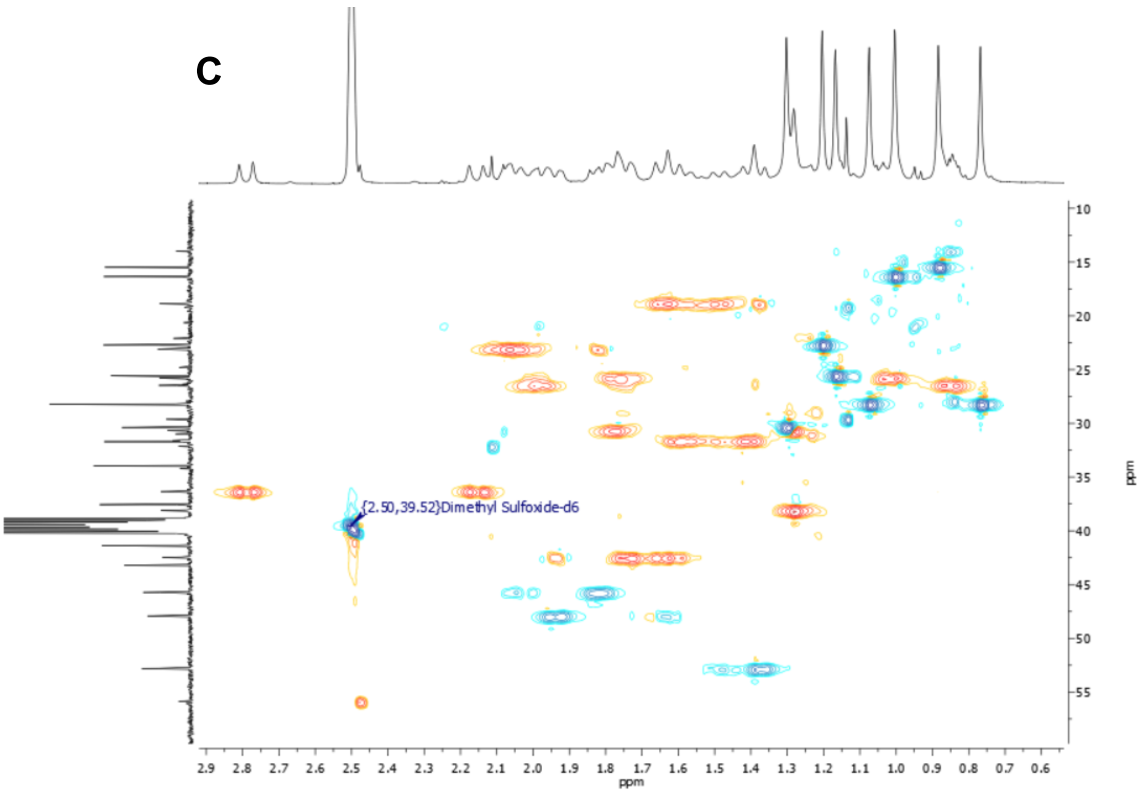

Figure S27. A) F u l l HSQC spectra of **3b** in DMSO-d_6. **B)** and **C)** Highfield spectra.

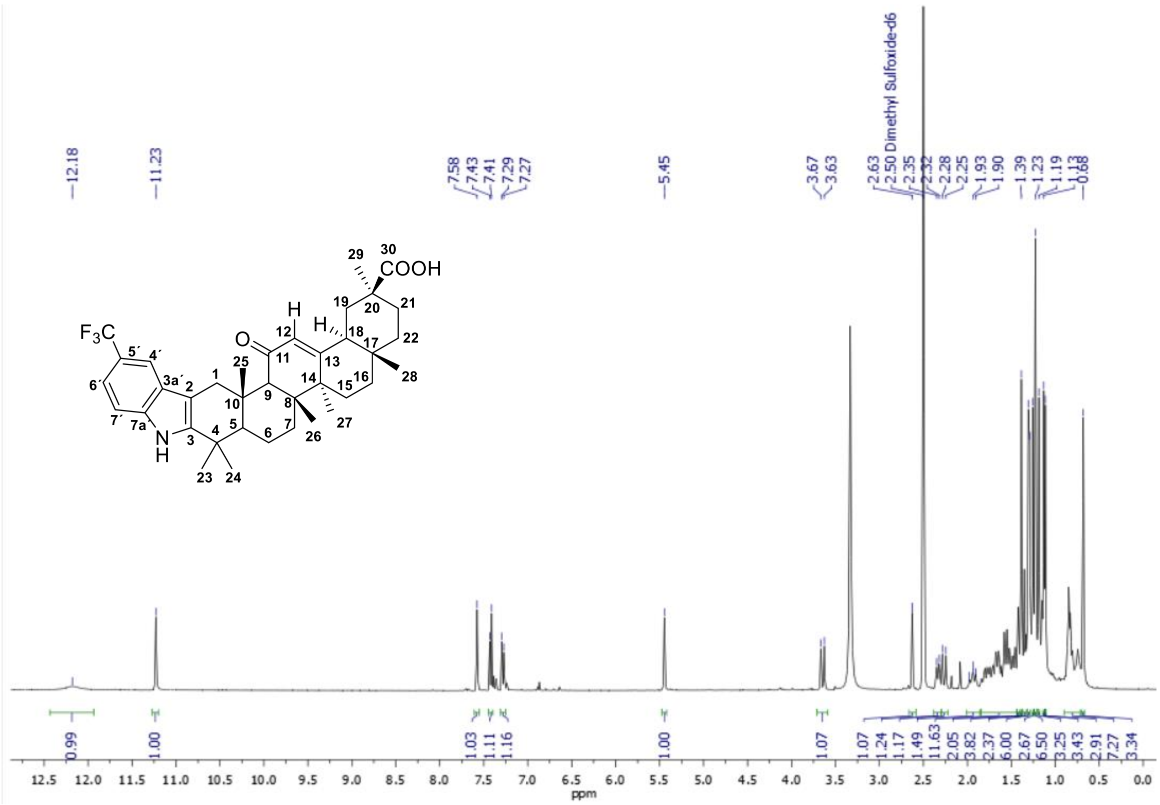

Figure S28. ^{1}H NMR (400 MHz) spectrum of compound **3c** in DMSO-d_6.

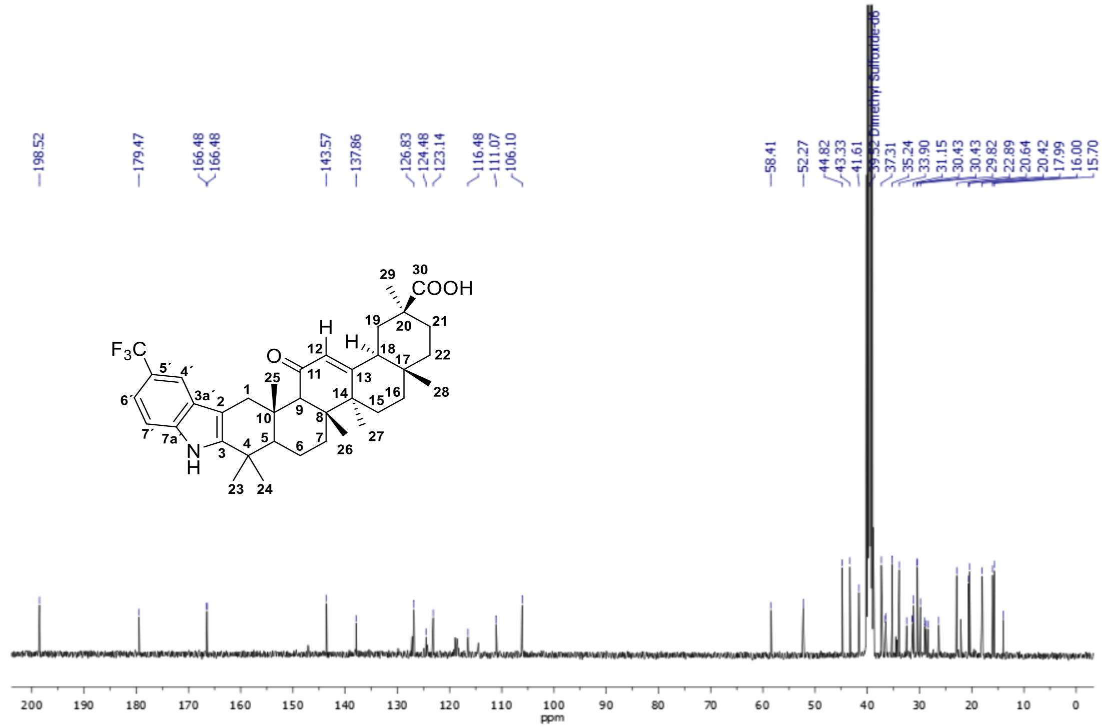

Figure S29. ^{13}C NMR (101 MHz) spectrum of compound **3c** in DMSO-d_6

A

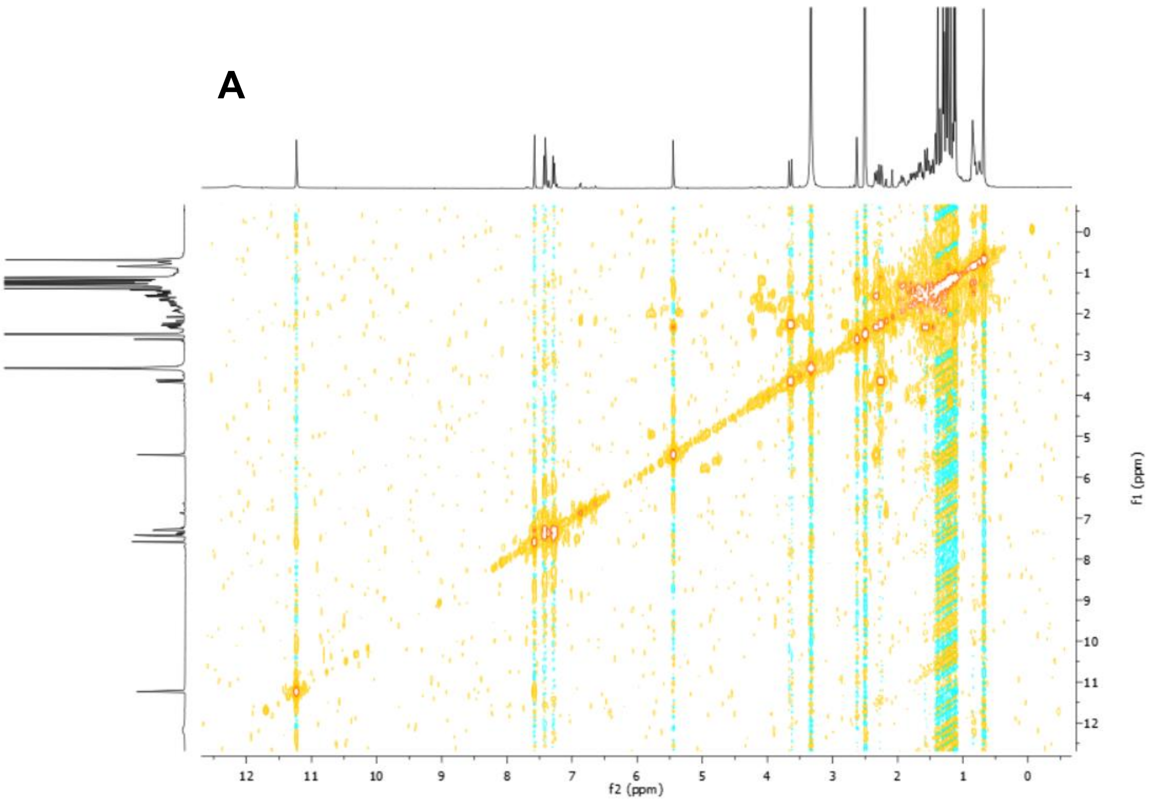

B

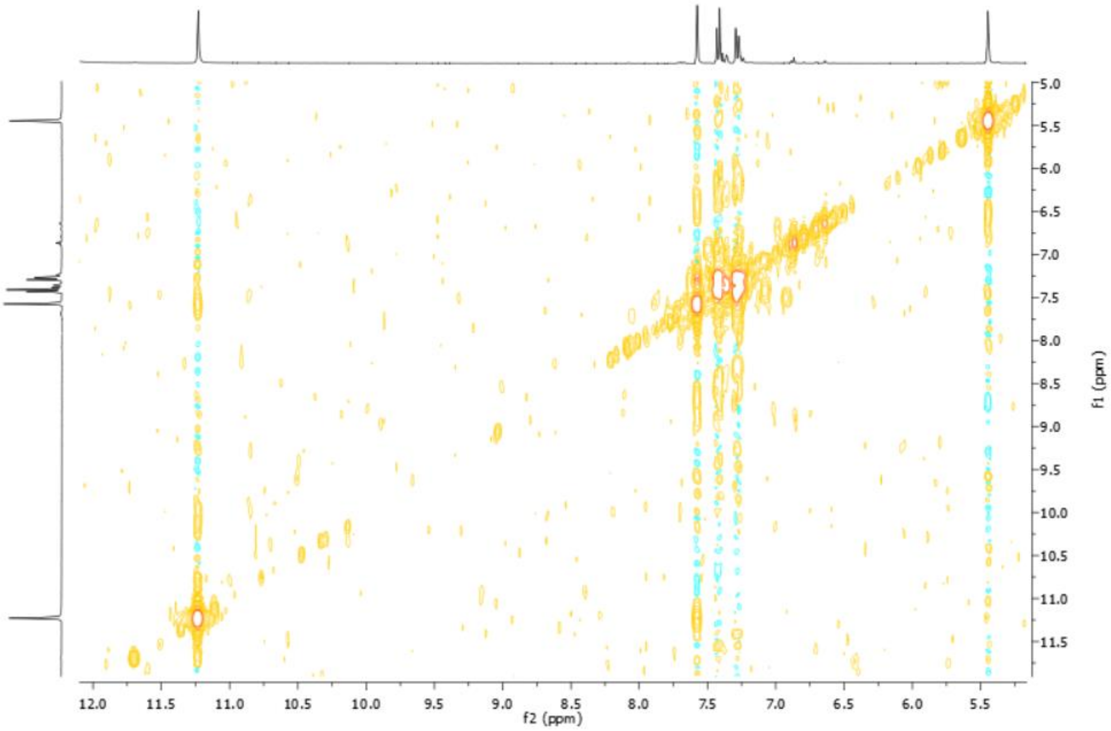

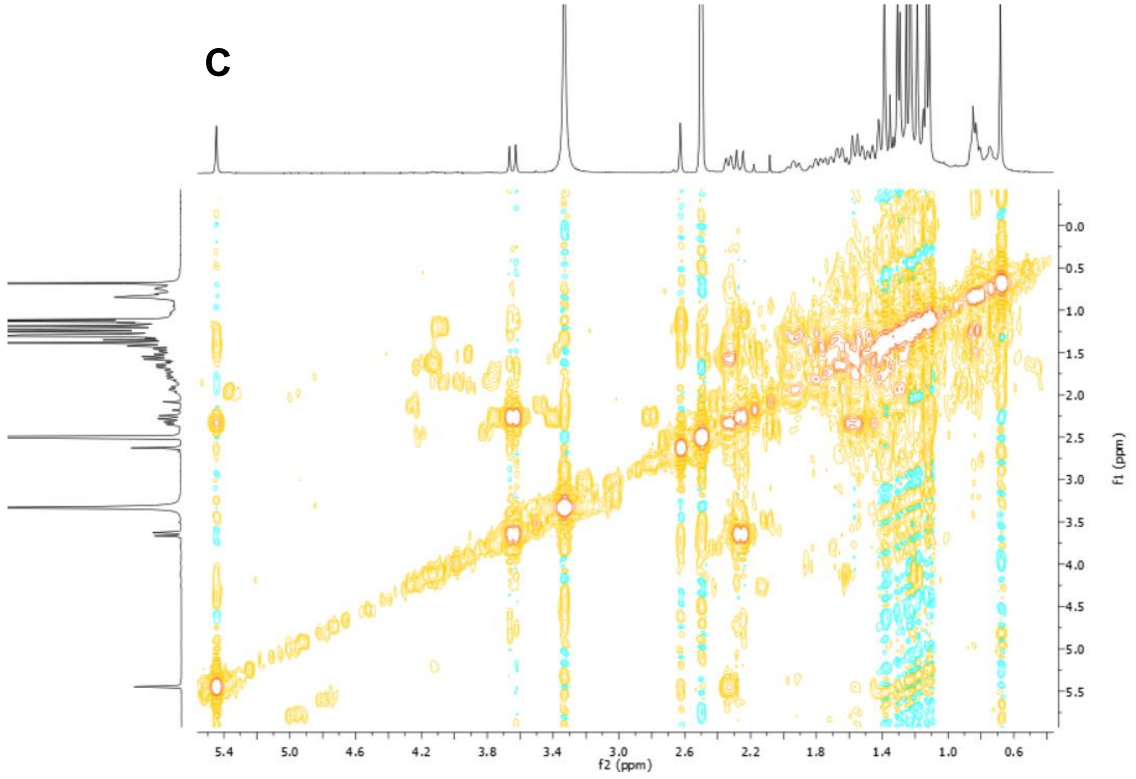

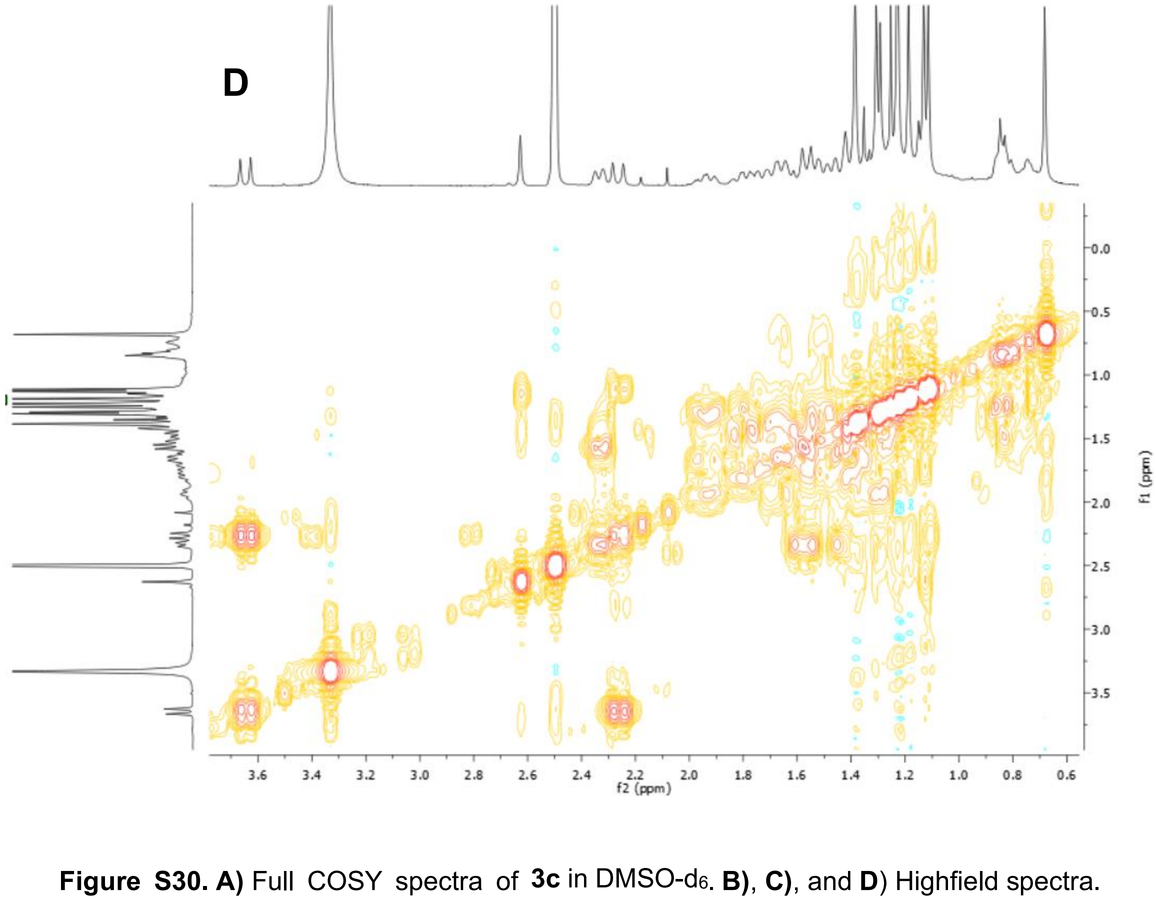

Figure S30. A) Full COSY spectra of **3c** in DMSO-d_6. **B)**, **C)**, and **D)** Highfield spectra.

A

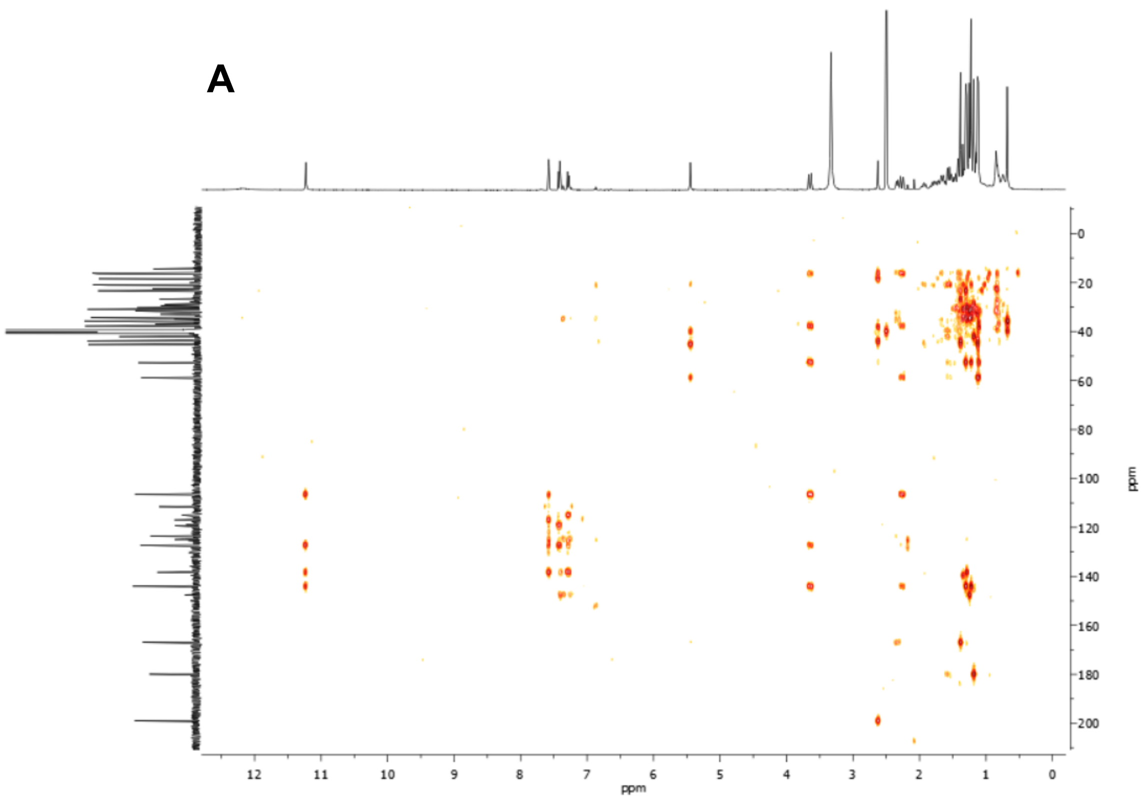
12
11
10
9
8
7
6
5
4
3
2
1
0
ppm
0
20
40
60
80
100
120
140
160
180
200
ppm

B

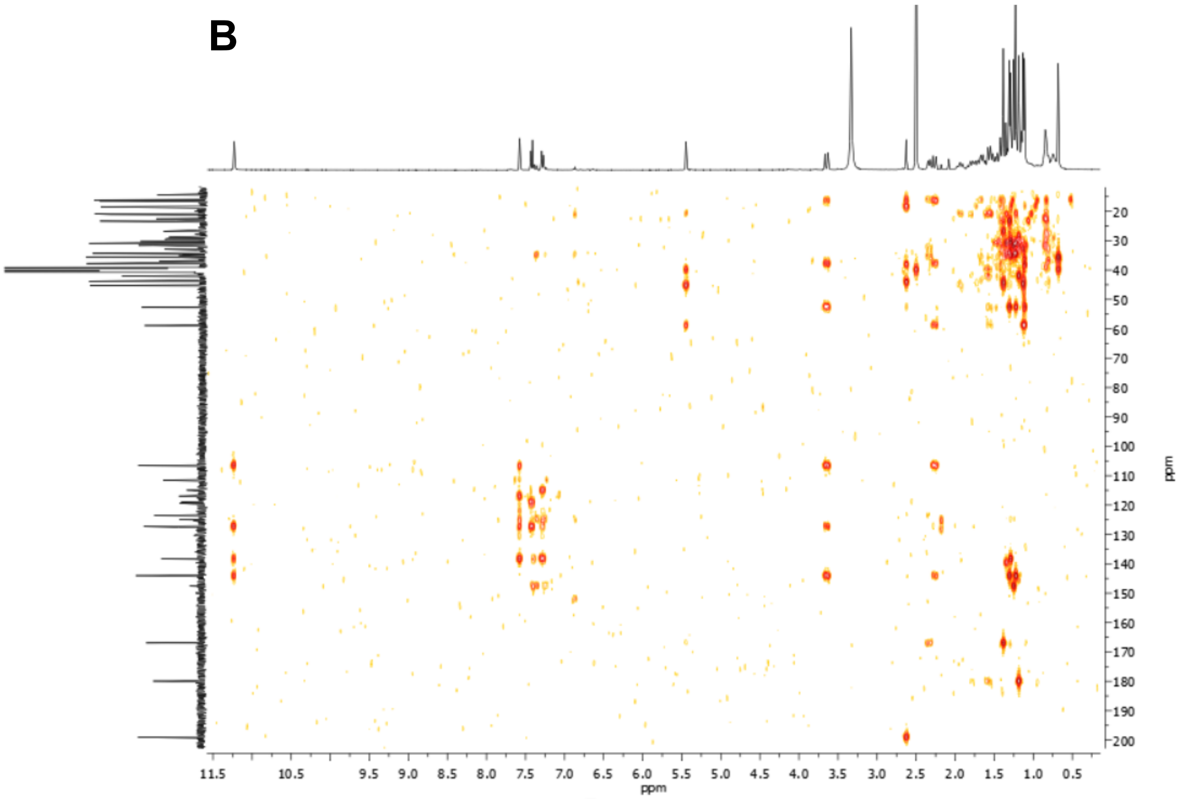
11.5
10.5
9.5
9.0
8.5
8.0
7.5
7.0
6.5
6.0
5.5
5.0
4.5
4.0
3.5
3.0
2.5
2.0
1.5
1.0
0.5
ppm
20
30
40
50
60
70
80
90
100
110
120
130
140
150
160
170
180
190
200
ppm

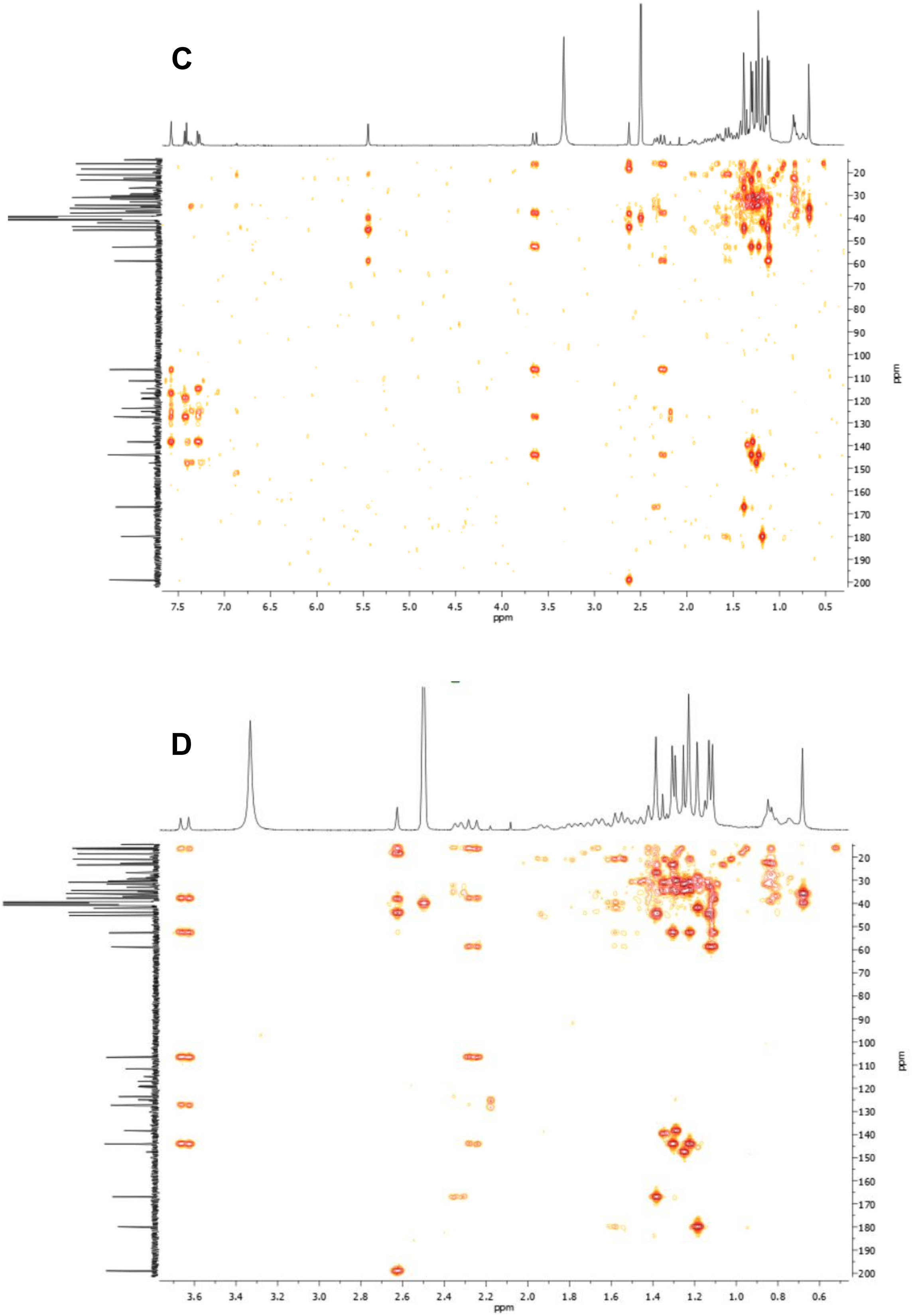

Figure S31. A) Full HMBC spectra of **3c** in DMSO-d_6. **B) C)** and **D**) Highfield spectra.

A

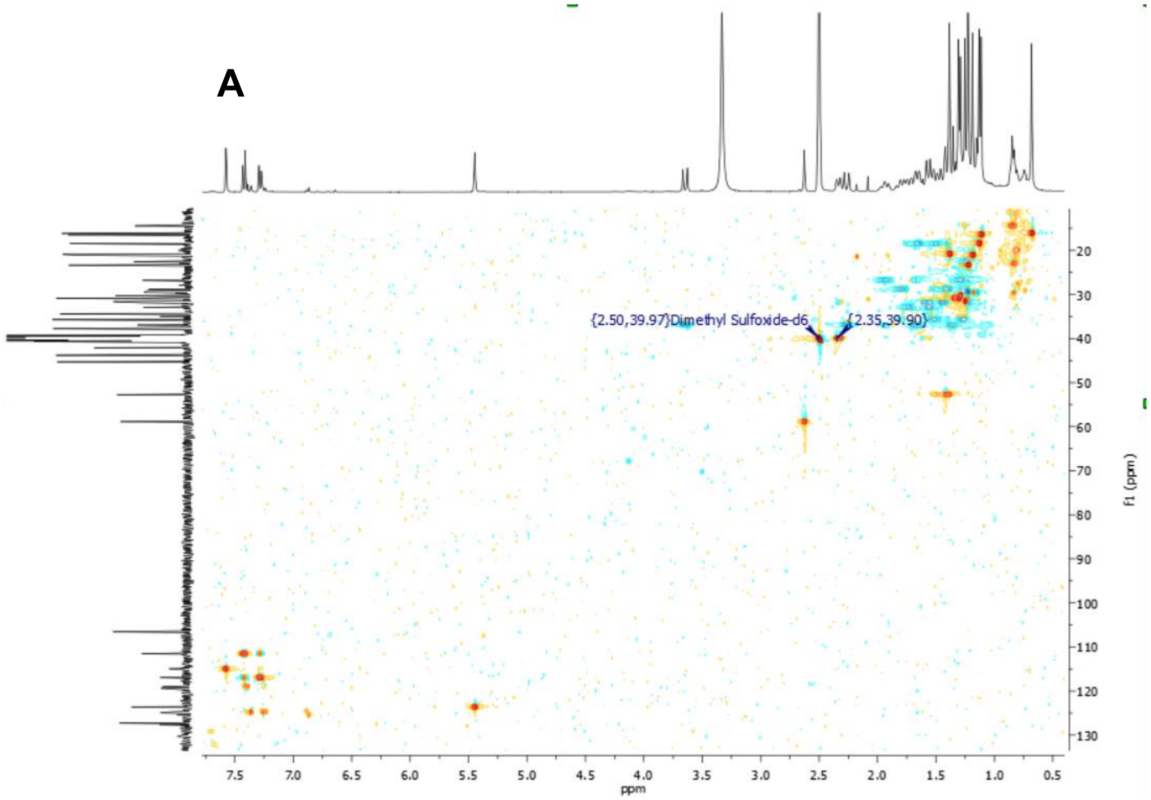

B

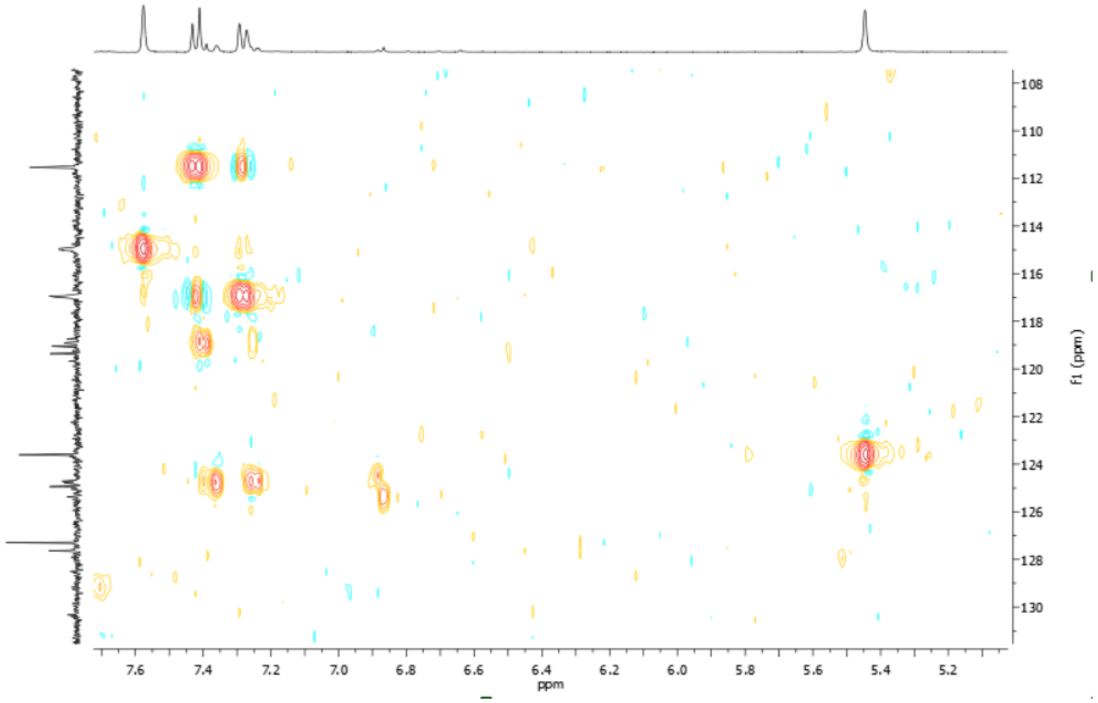

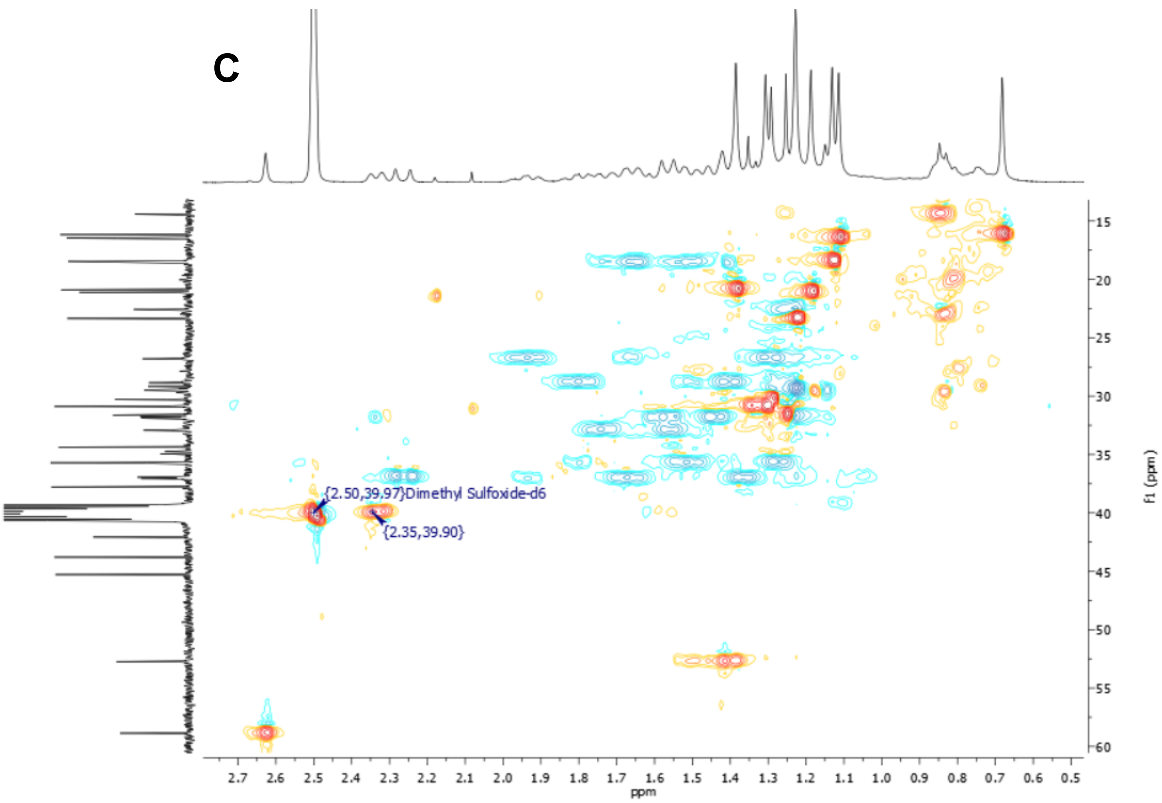

Figure S32. A) Full HSQC spectra of **3c** in DMSO-d6. **B)** and **C**) Highfield spectra.

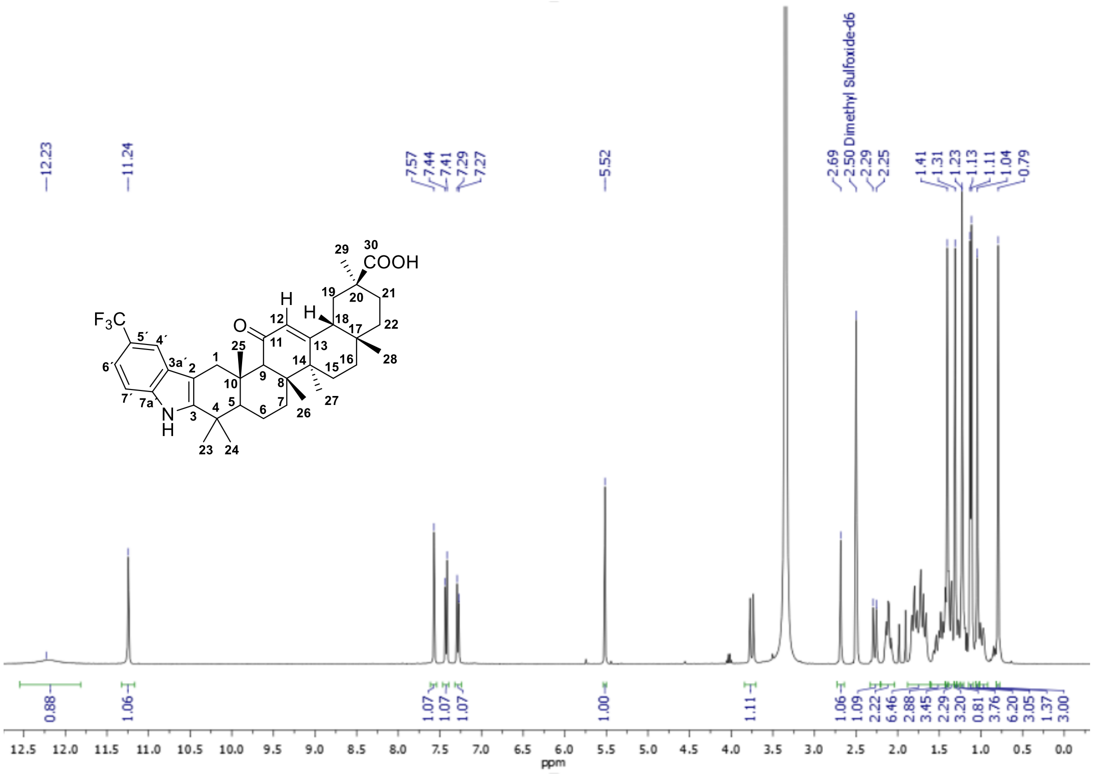

Figure S33. ^{1}H NMR (400 MHz) spectrum of compound **FC-114** in DMSO-d6.

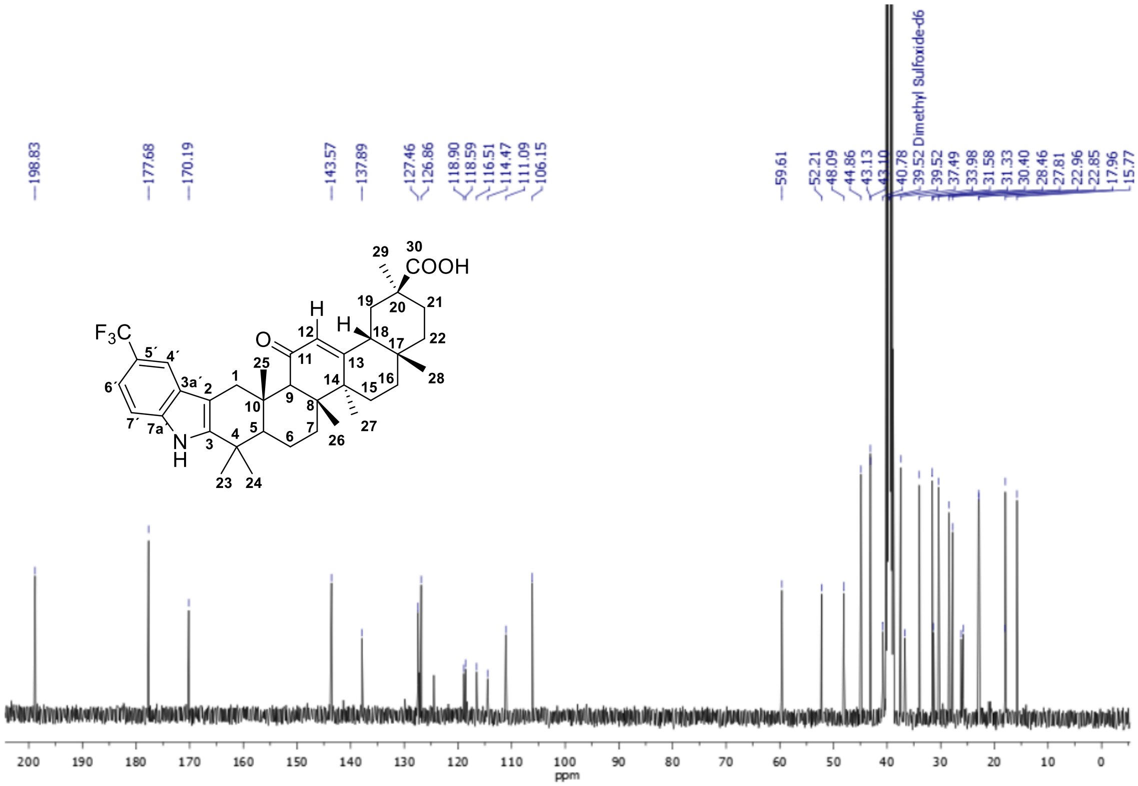

Figure S34. ^{13}C NMR (101 MHz) spectrum of compound **FC114** in DMSO-d_6.

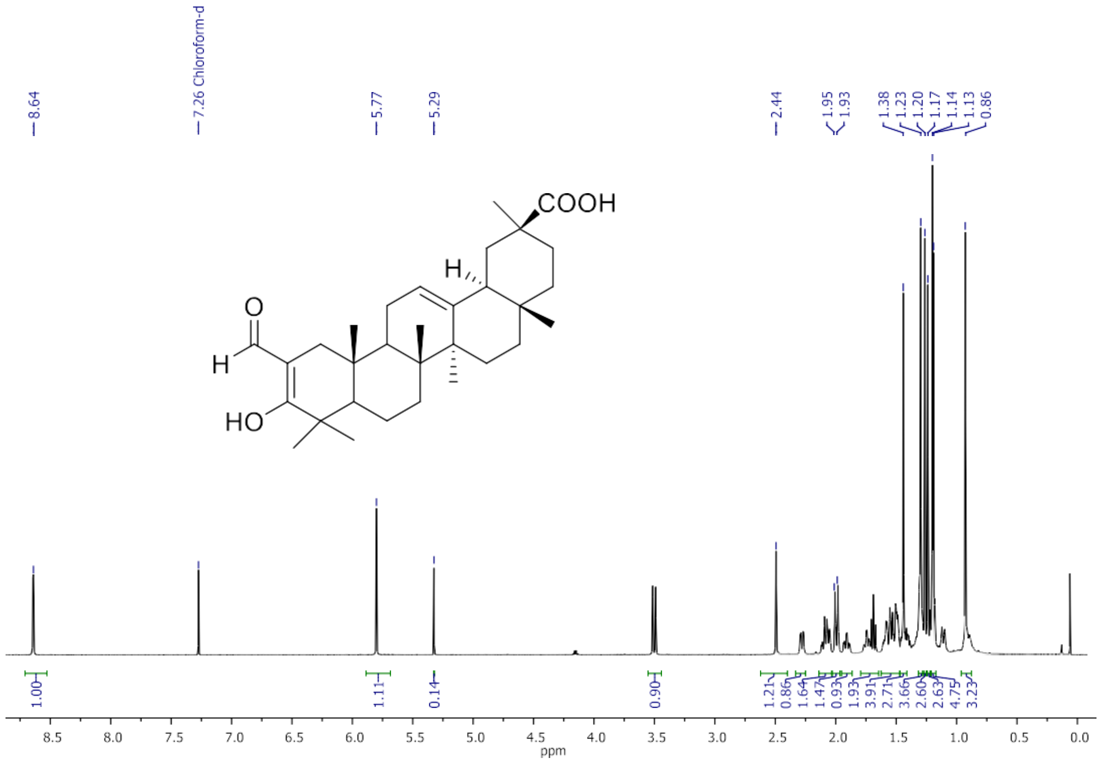

Figure S35. ^{1}H NMR (600 MHz) spectrum of compound **4a** in $CDCl_3$.

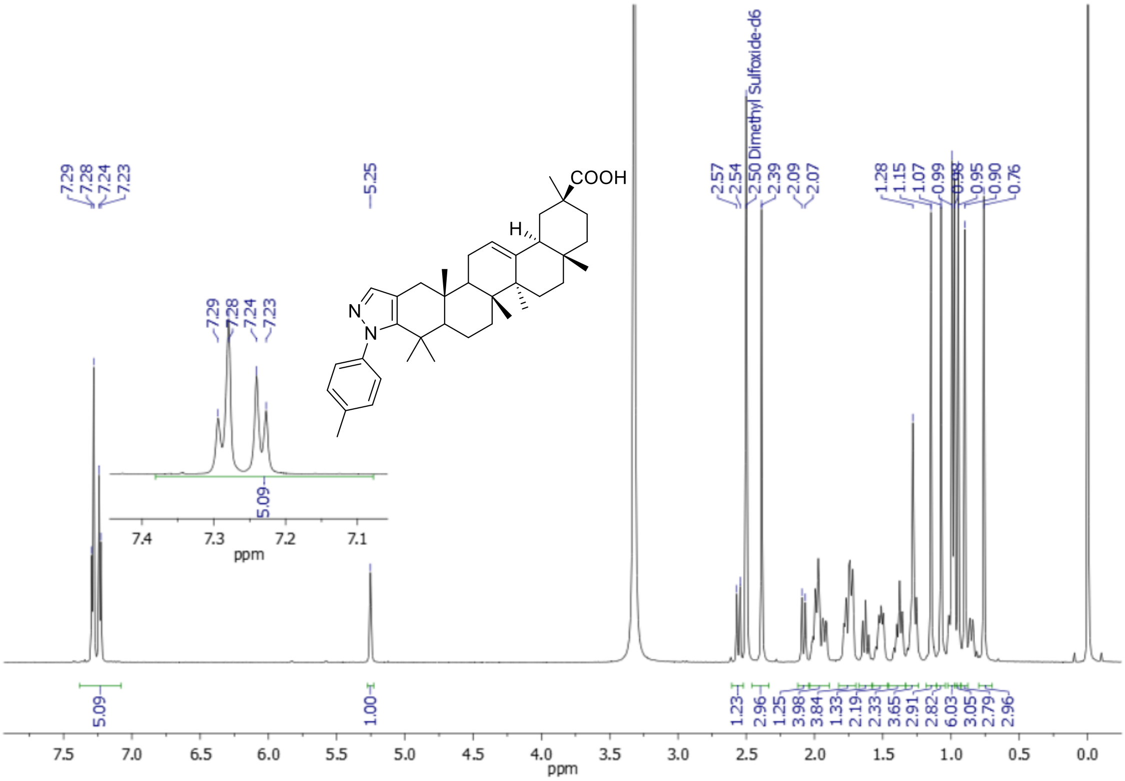

Figure S36. ^{1}H NMR (600 MHz) spectrum of compound **5a** in DMSO-d_6.

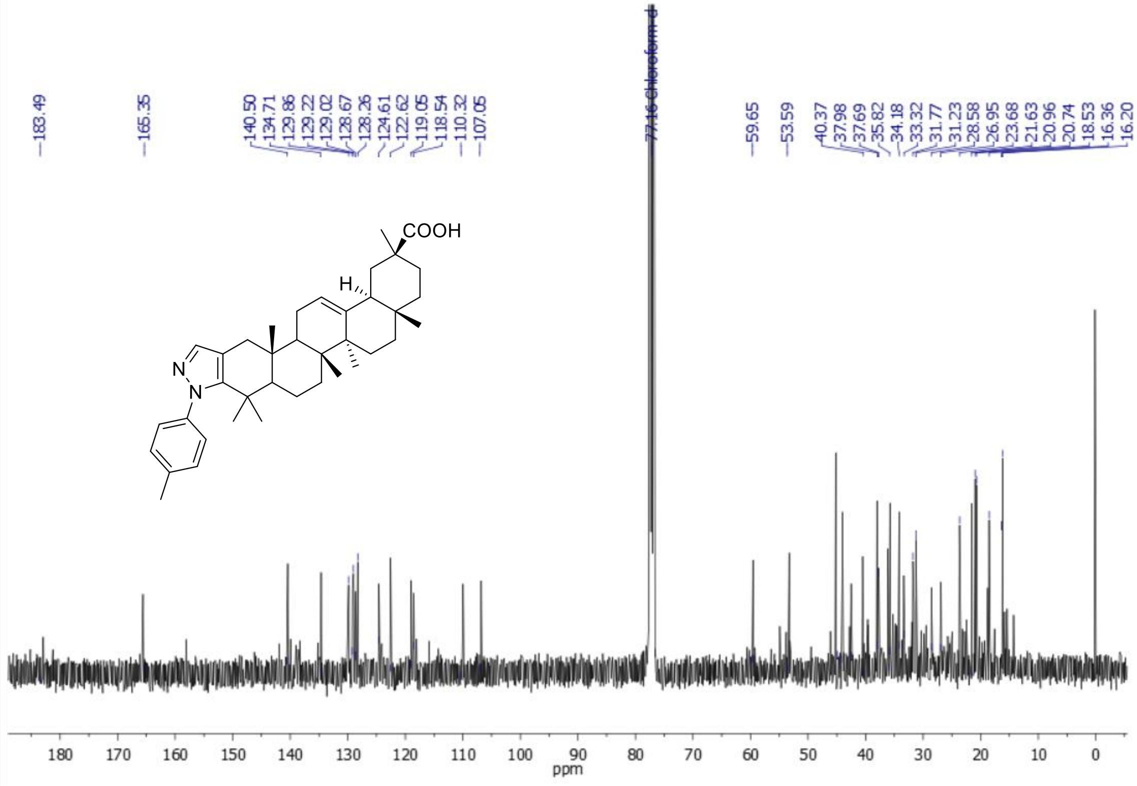

Figure S37. ^{13}C NMR (101 MHz) spectrum of compound **5a** in $CDCl_3$.

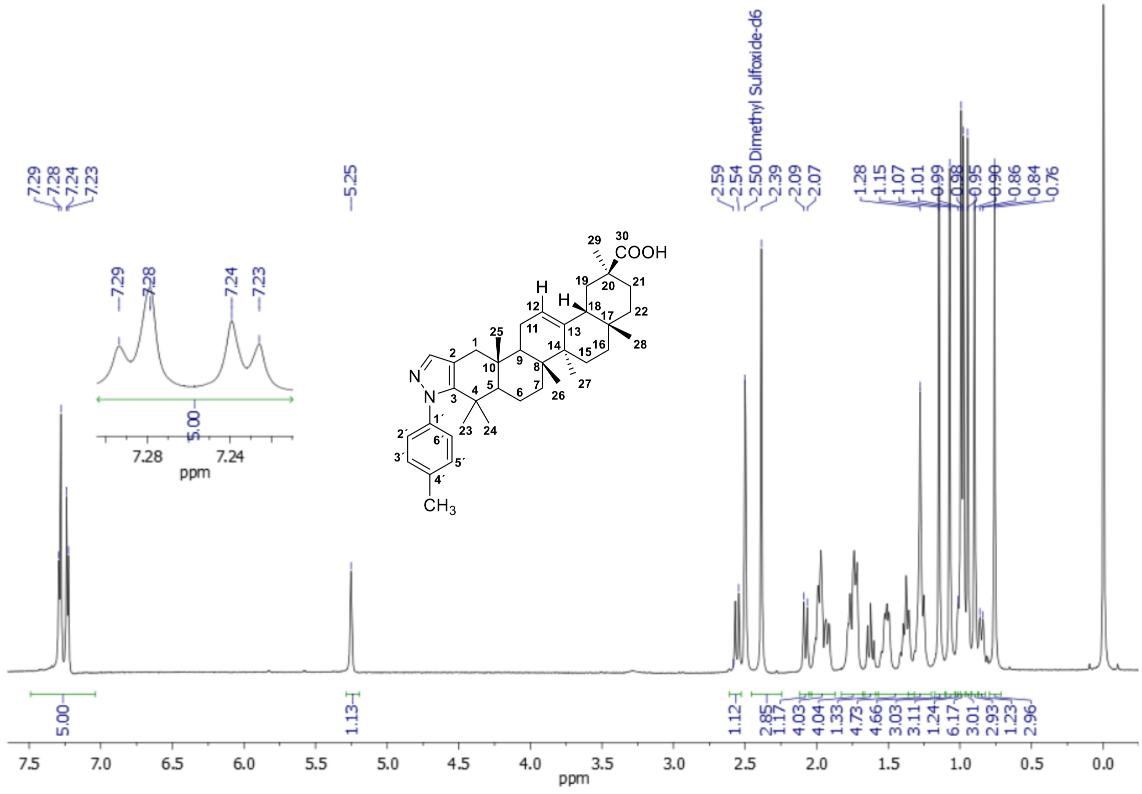

Figure S38. ^{1}H NMR (600 MHz) spectrum of compound **5b** in DMSO-d_6.

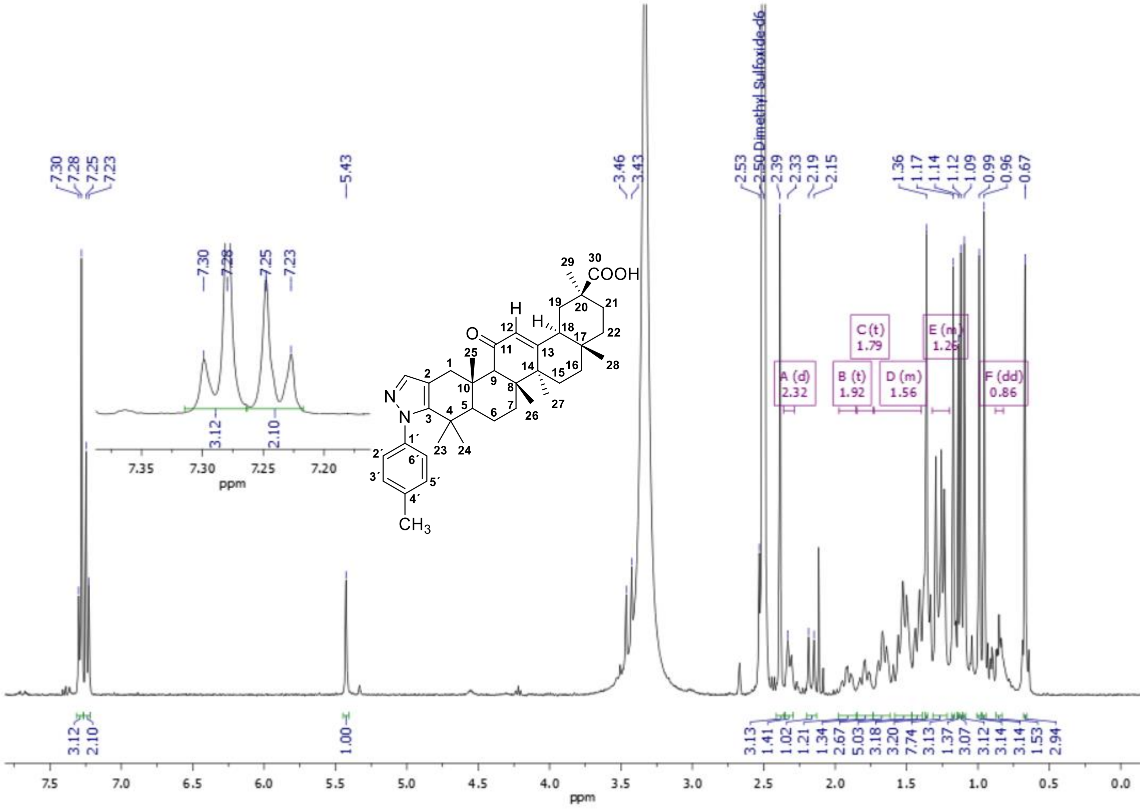

Figure S39. ^{1}H NMR (400 MHz) spectrum of compound **5c** in DMSO-d_6.

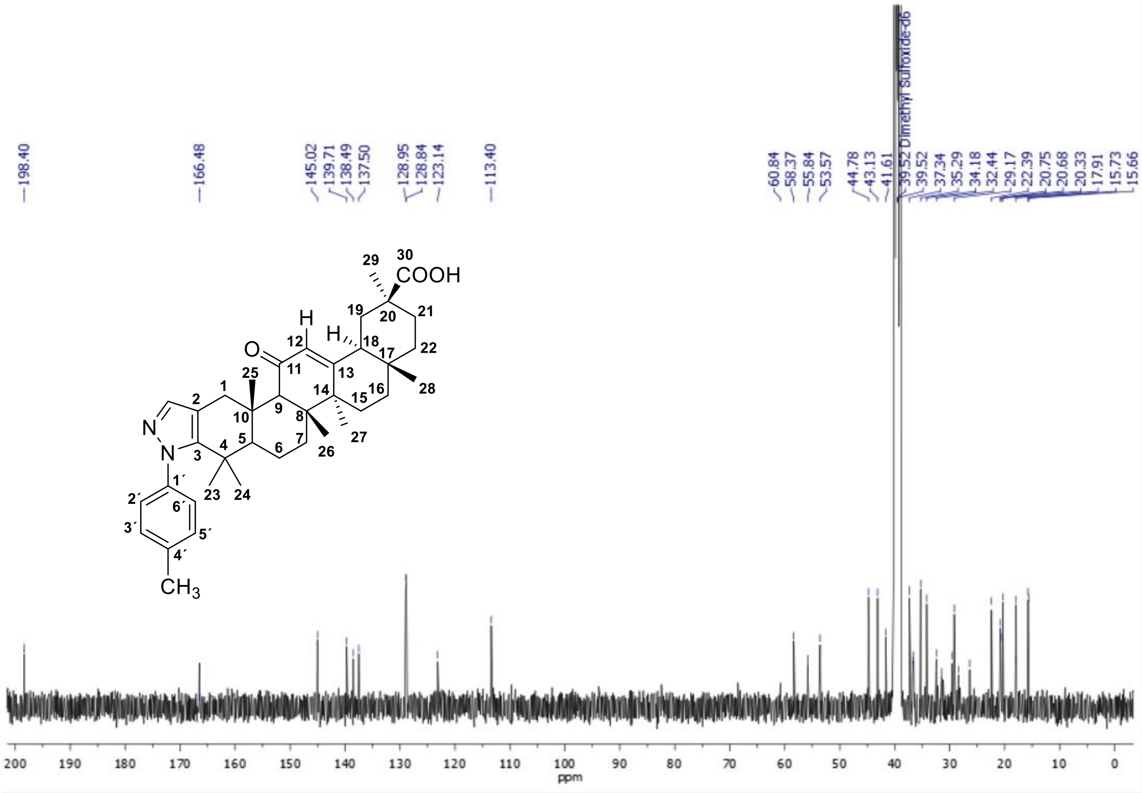

Figure S40. ^{13}C NMR (101 MHz) spectrum of compound **5c** in DMSO-d_6.

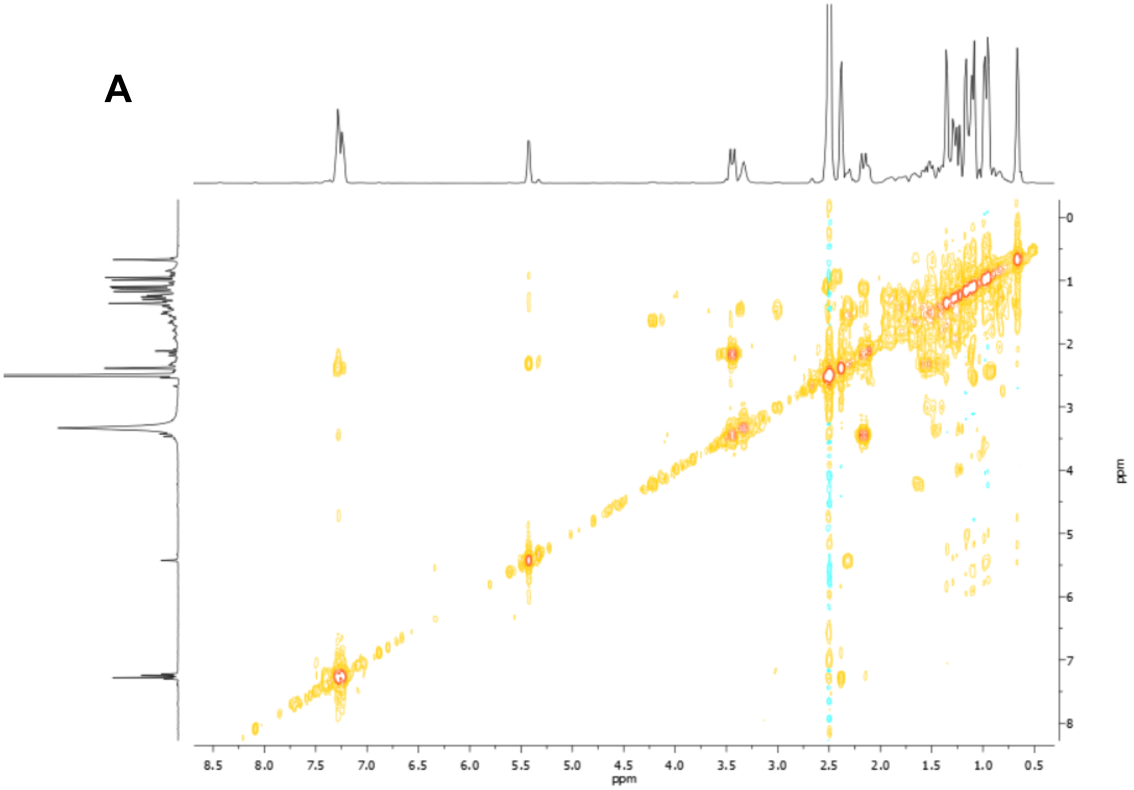

B

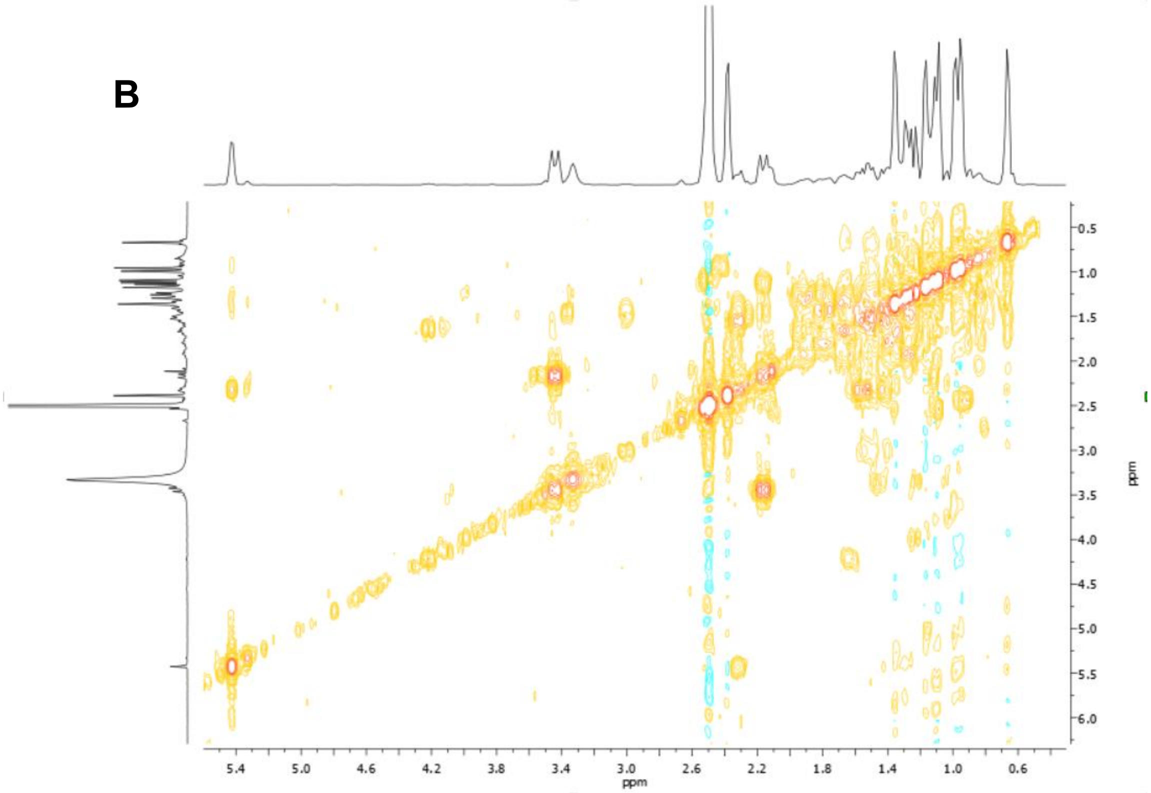

C

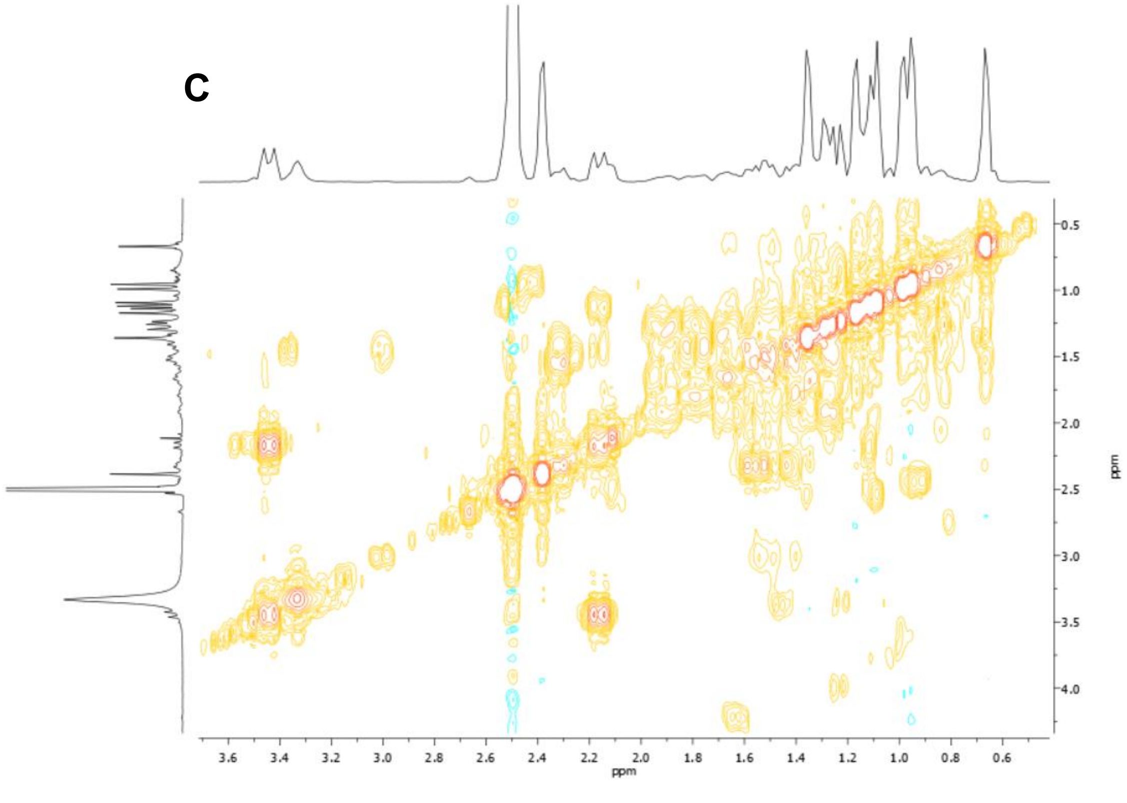

D

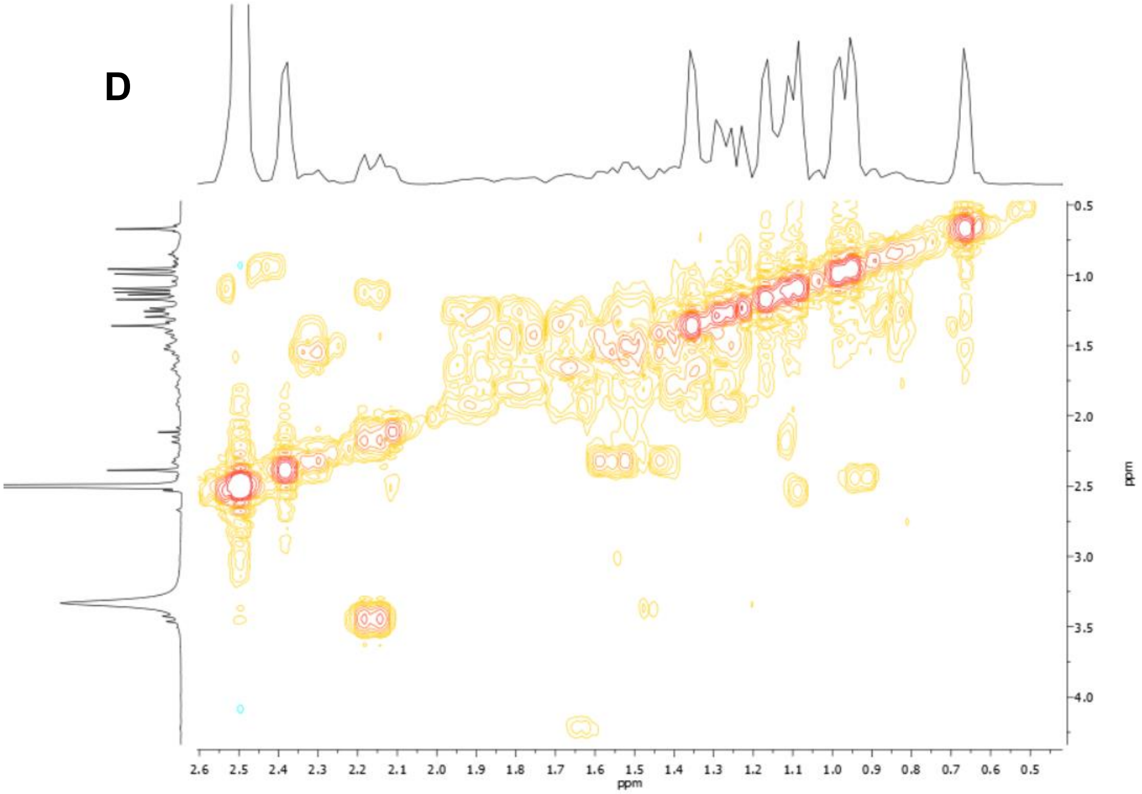

Figure S41. A) Full COSY spectra of **5c** in DMSO-d_6. **B), C),** and **D**) Highfield spectra.

A

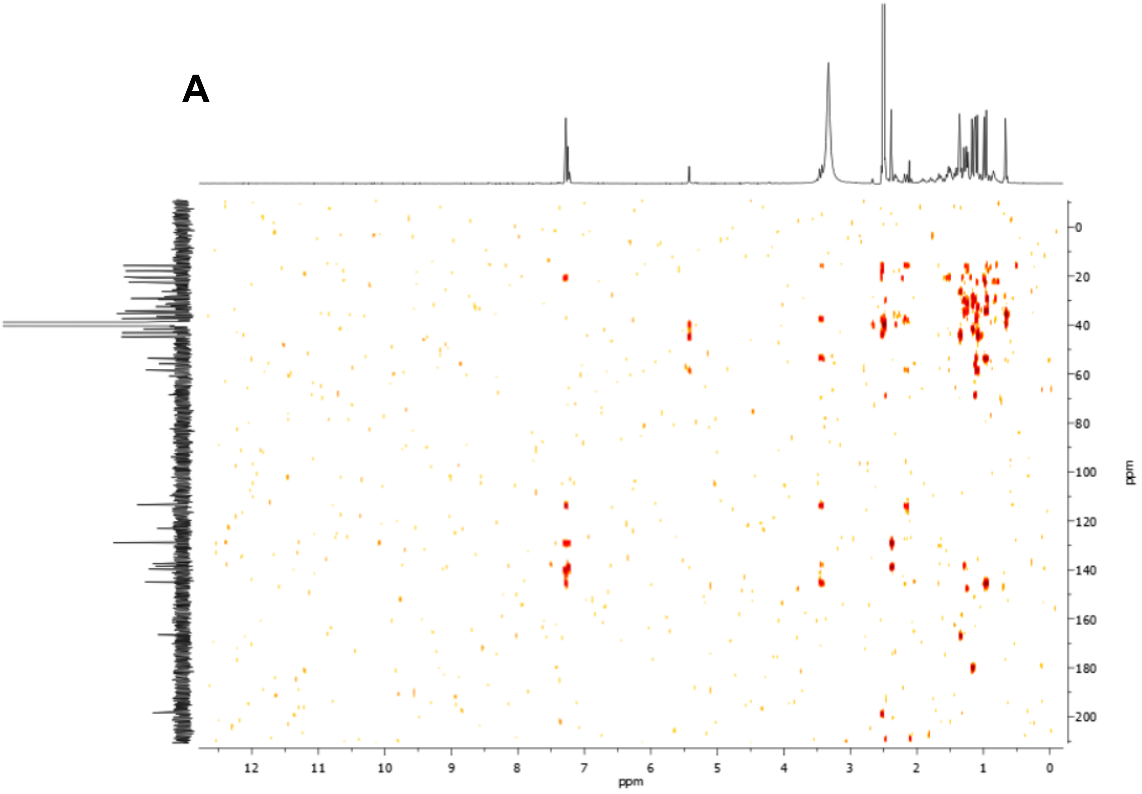

B

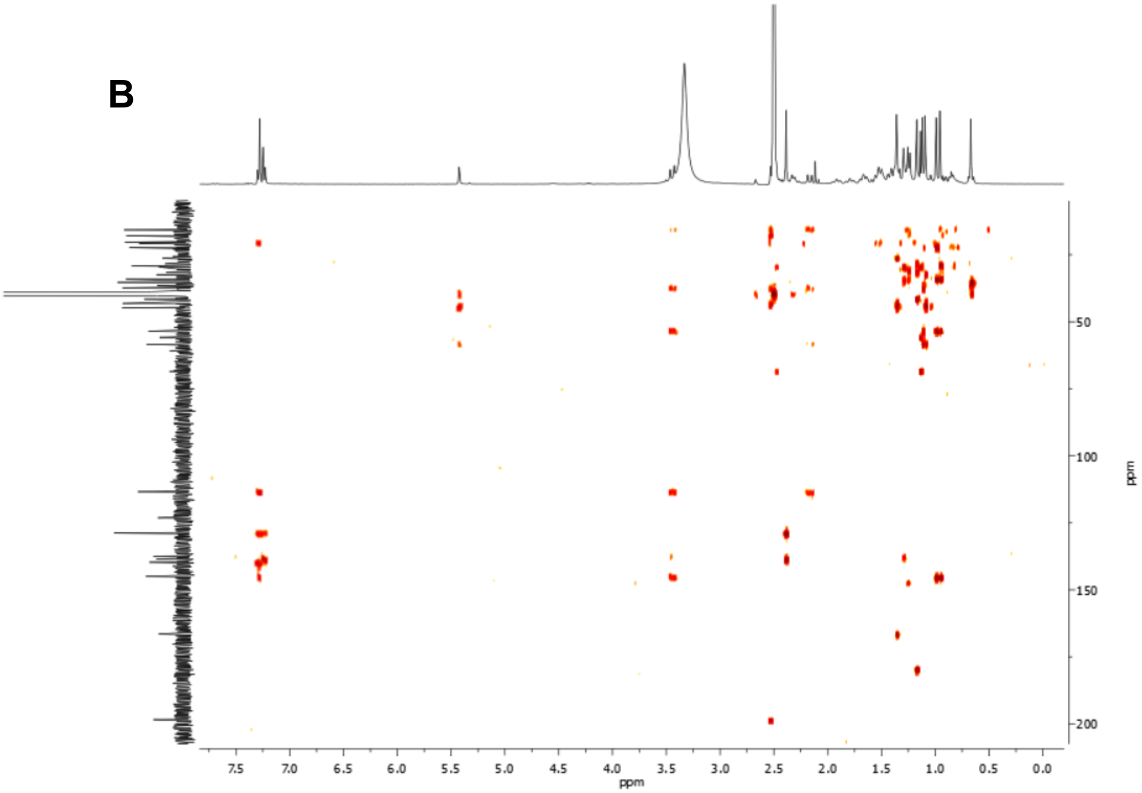

C

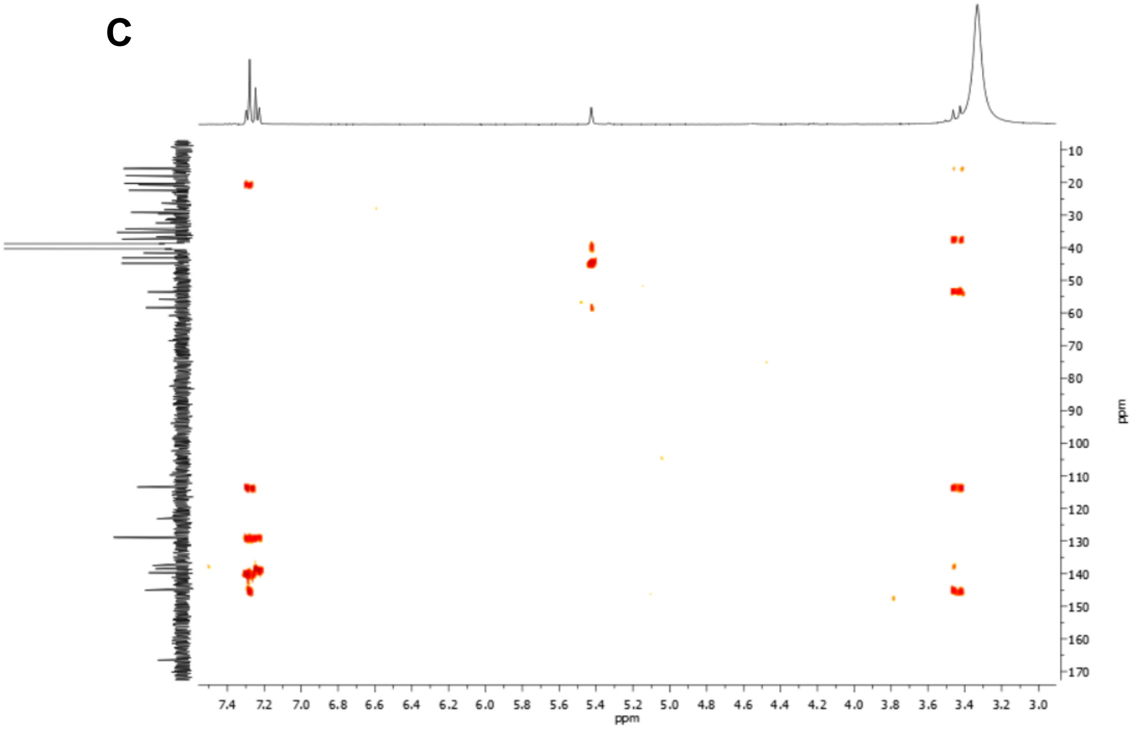

D

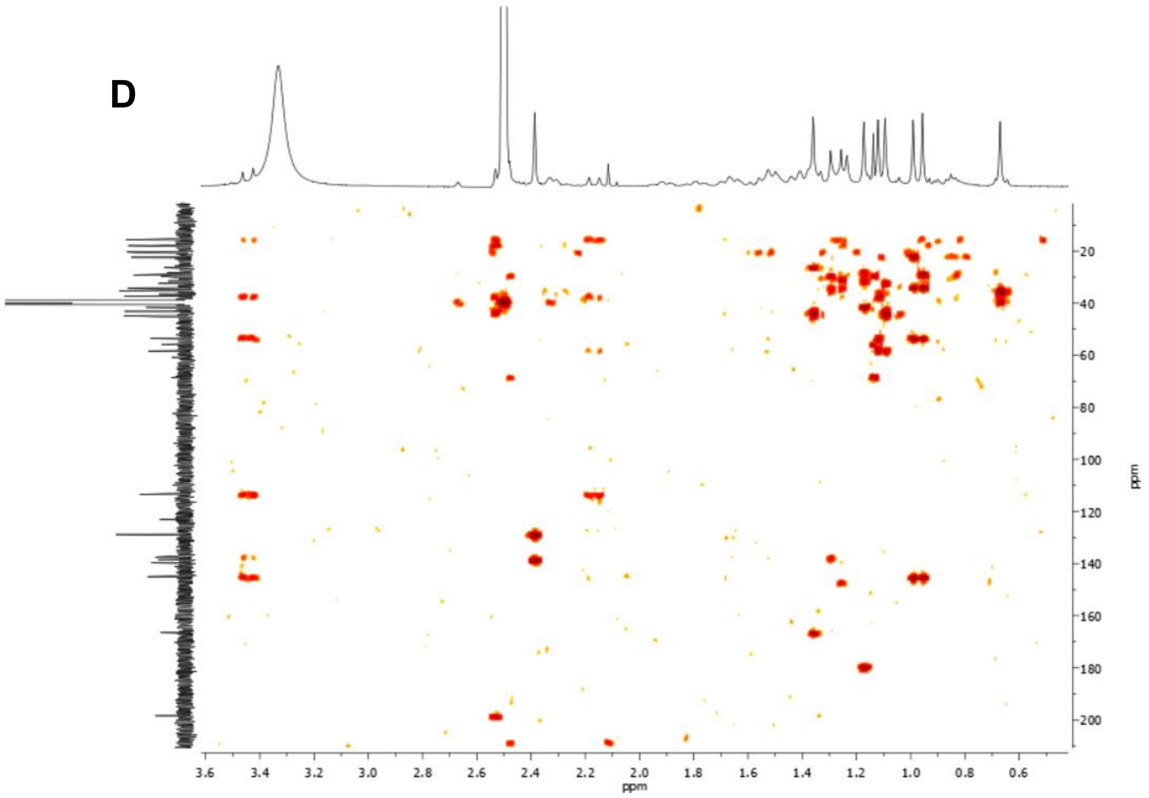

Figure S42. Full HMBC spectra of **5c** in DMSO-d_6. **B), C),** and **D**) Highfield spectra.

A

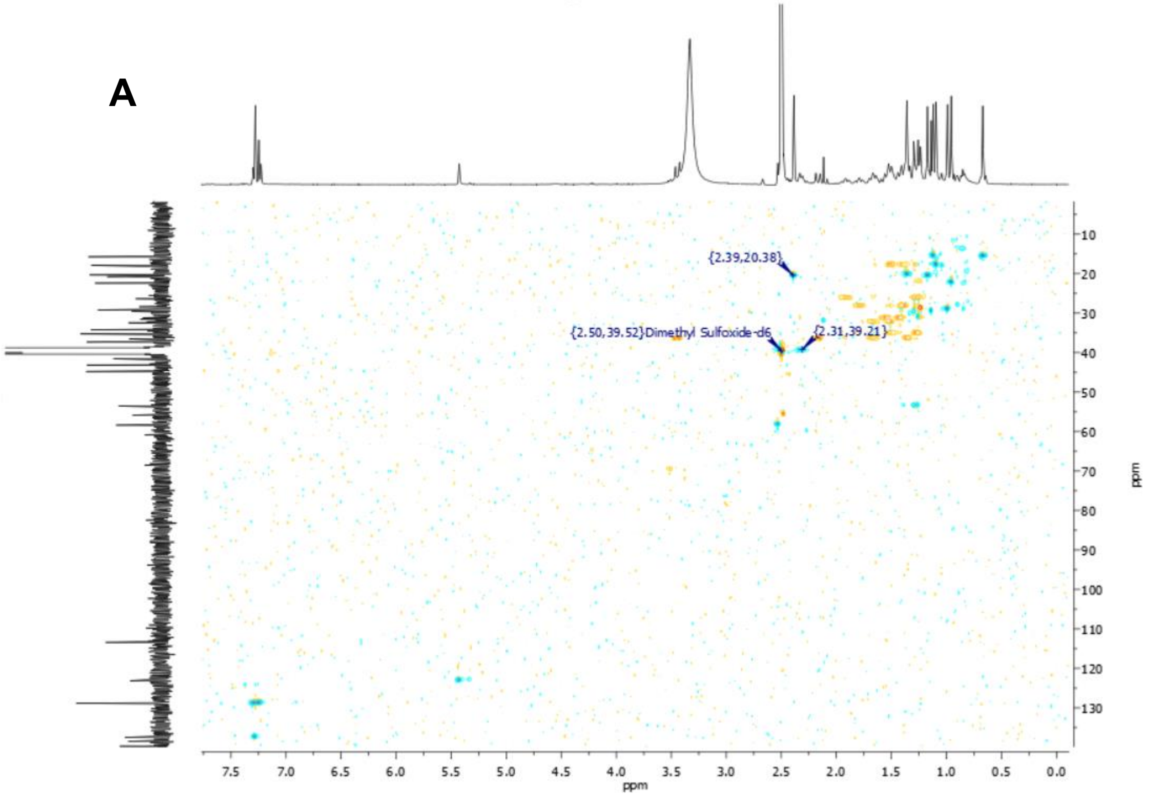

B

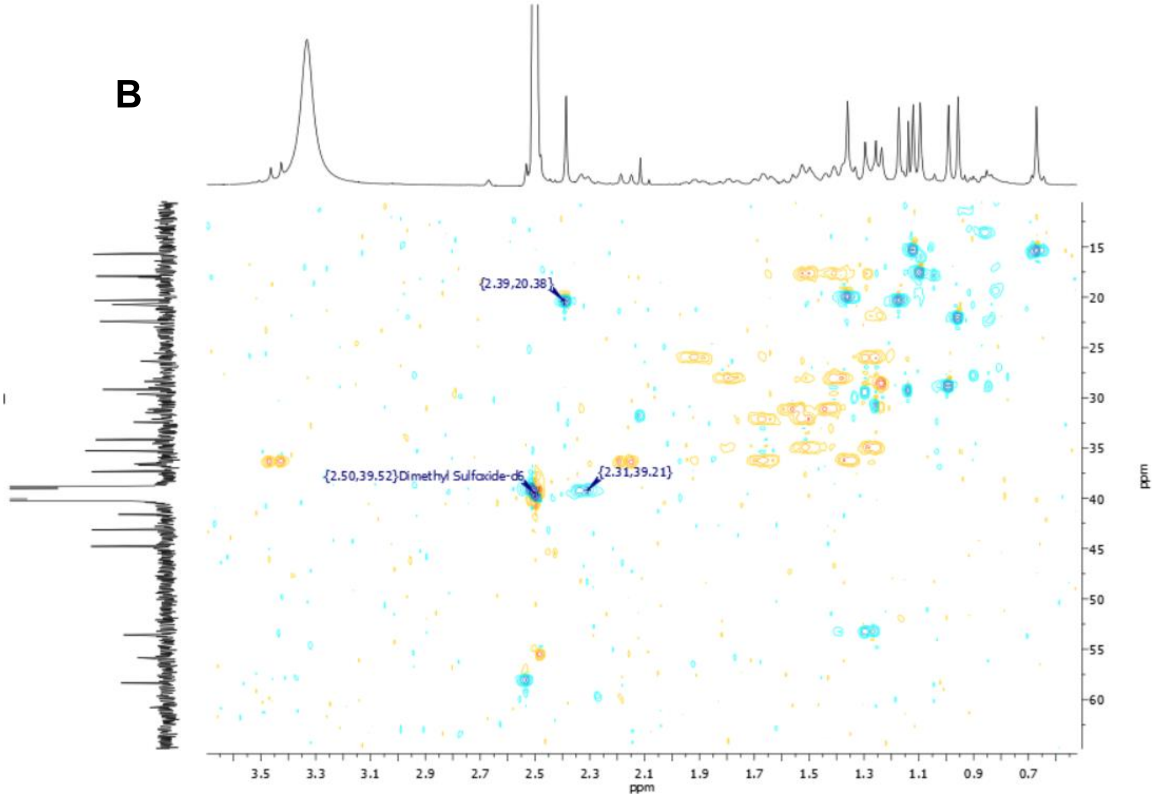
{2.39,20.38}
{2.50,39.52}Dimethyl Sulfoxide-d6
{2.31,39.21}
ppm
ppm

C

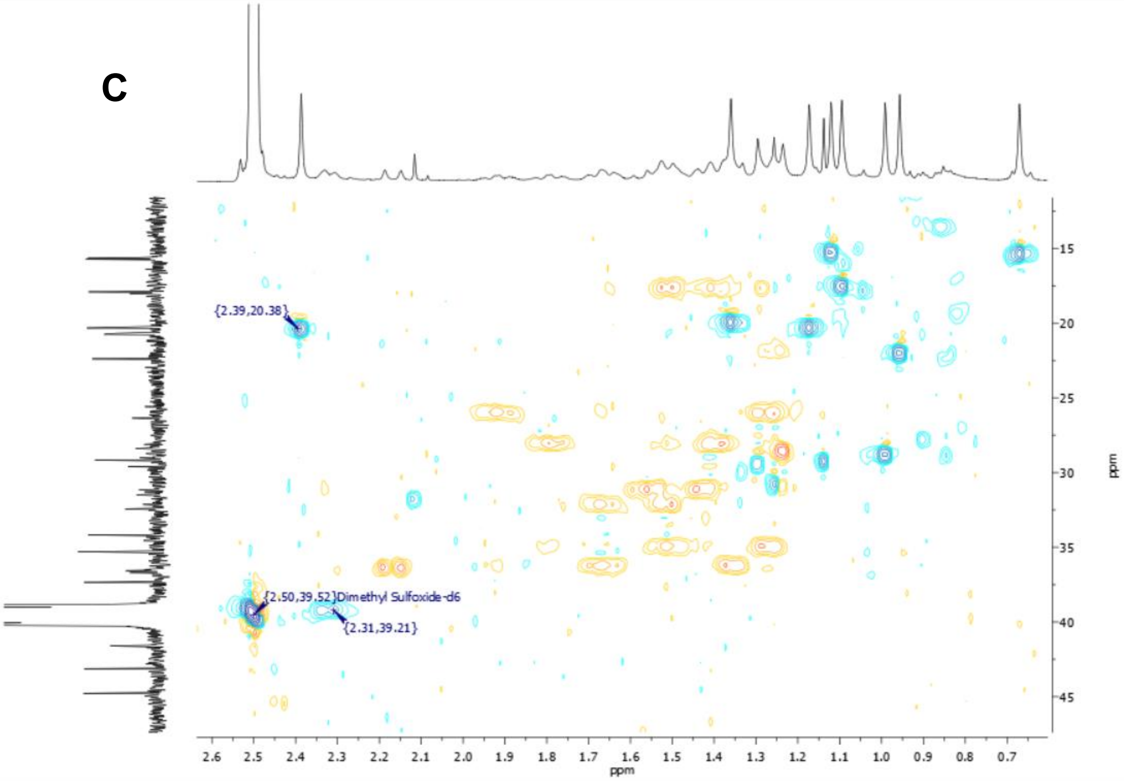
{2.39,20.38}
{2.50,39.52}Dimethyl Sulfoxide-d6
{2.31,39.21}
ppm
ppm

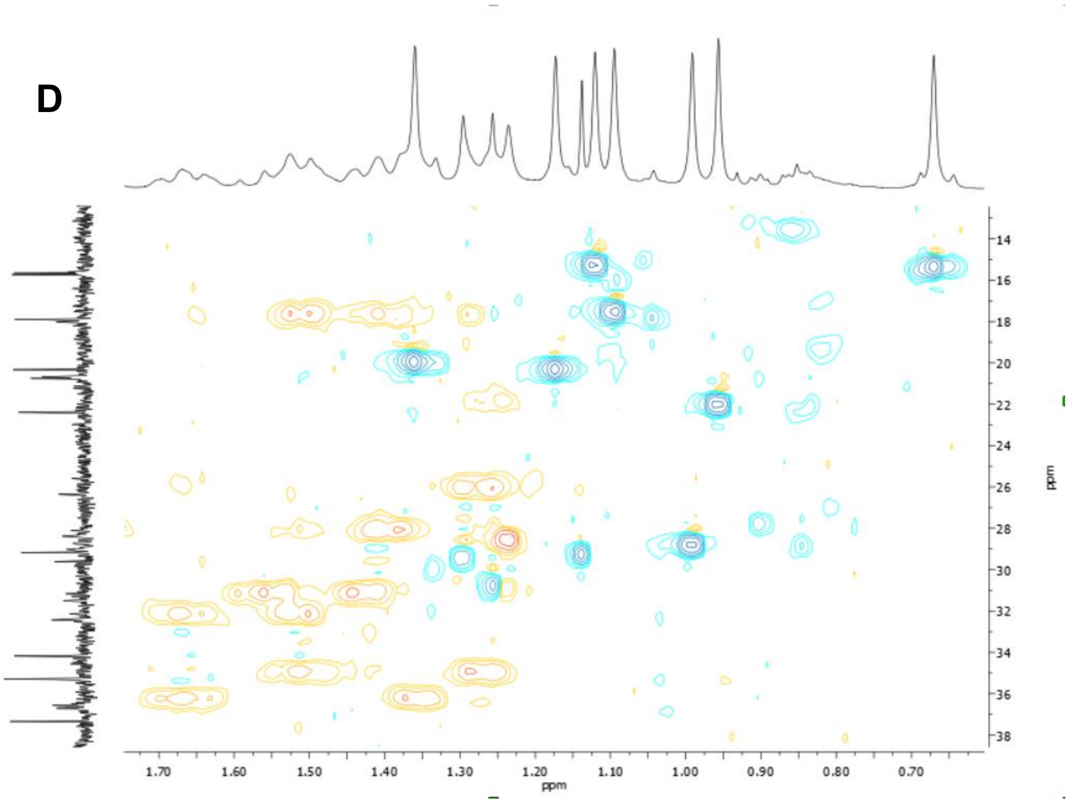

Figure S43. A) Full HSQC spectra of **5c** in DMSO-d6. **B), C),** and **D**) Highfield spectra.

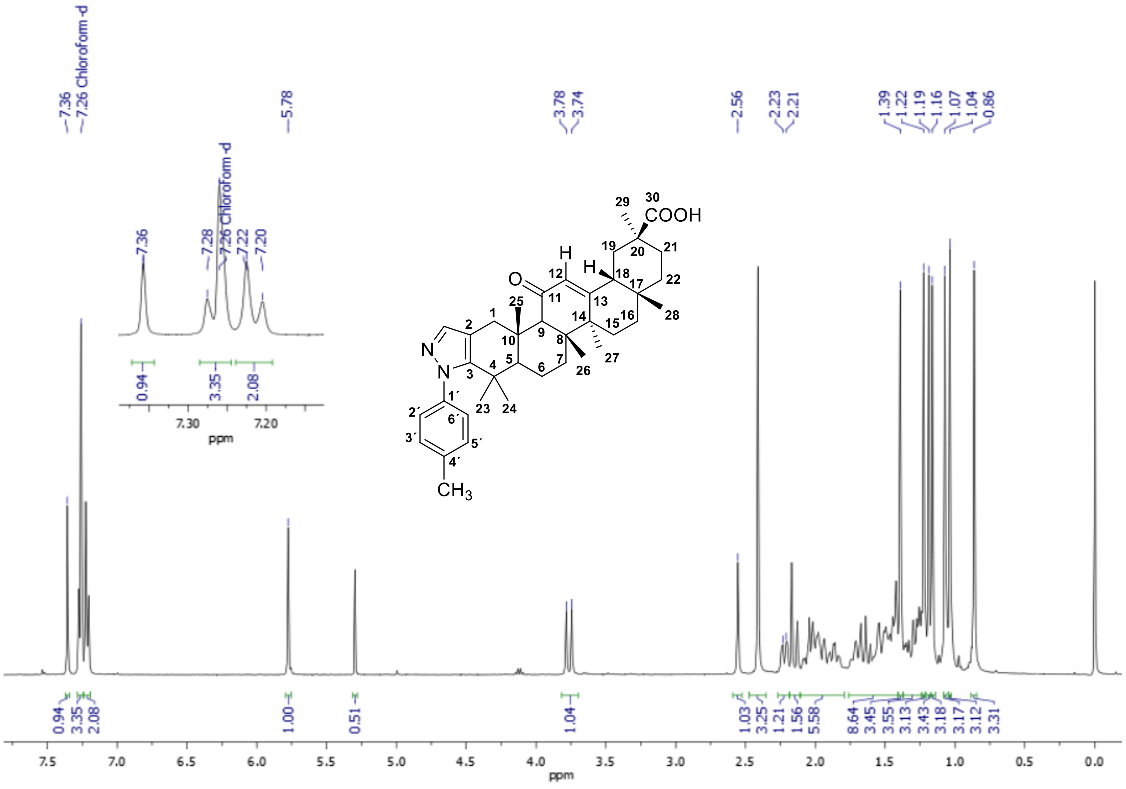

Figure S44. ^{1}H NMR (400 MHz) spectrum of compound **FC-122** in DMSO-d6.

Table S1. Crystal data, structure solution, and refinement parameters for compound **4d**

Empirical formula	$C_{31}H_{44}O_5$
Formula weight	496.66
Temperature/K	293
Crystal system	orthorhombic
Space group	$P2_12_12_1$
a/Å	8.52250(10)
b/Å	11.18080(10)
c/Å	28.7762(4)
α/°	90
β/°	90
γ/°	90
Volume/Å^3	2742.04(6)
Z	4
ρ_{calc}g/cm^3	1.203
μ/mm^{-1}	0.632
F(000)	1080
Crystal size/mm^3	0.5 × 0.5 × 0.4
Radiation	Cu Kα (λ = 1.54184)
2Θ range for data collection/°	6.142 to 155.194
Index ranges	-10 ≤ h ≤ 10, -14 ≤ k ≤ 14, -35 ≤ l ≤ 36
Reflections collected	35867
Independent reflections	5802 [R_{int} = 0.0247, R_{sigma} = 0.0122]
Data/restraints/parameters	5802/2/340
Goodness-of-fit on F^2	1.055
Final R indexes [I>=2σ (I)]	R_1 = 0.0429, wR_2 = 0.1161
Final R indexes [all data]	R_1 = 0.0435, wR_2 = 0.1171
Largest diff. peak/hole / e Å^{-3}	0.23/-0.26
Flack parameter	-0.04(4)

Table S2. Crystal data, structure solution, and refinement parameters for compound **5b**

**Compound 5b**	
Empirical formula	$C_{76}H_{104}N_4O_4$
Formula weight	1137.63
Temperature/K	295
Crystal system	orthorhombic
Space group	$P2_12_12_1$
a/Å	7.8759(8)
b/Å	12.3317(12)
c/Å	68.630(5)
α/°	90
β/°	90
γ/°	90
Volume/Å^3	6665.5(11)
Z	4
ρ_{calc}g/cm^3	1.134
μ/mm^{-1}	0.529
F(000)	2480
Crystal size/mm^3	0.2 × 0.1 × 0.05
Radiation	Cu Kα (λ = 1.54184)
2Θ range for data collection/°	7.284 to 156.658
Index ranges	-9 ≤ h ≤ 9, -15 ≤ k ≤ 11, -85 ≤ l ≤ 79
Reflections collected	32962
Independent reflections	13870 [R_{int} = 0.1278, R_{sigma} = 0.1789]
Data/restraints/parameters	13870/4/781
Goodness-of-fit on F^2	0.974
Final R indexes [I>=2σ (I)]	R_1 = 0.0915, wR_2 = 0.1819
Final R indexes [all data]	R_1 = 0.1834, wR_2 = 0.2597
Largest diff. peak/hole / e Å^{-3}	0.17/-0.28
Flack parameter	-0.8(7)

Mass Spectrum SmartFormula Report

Analysis Info

Analysis Name	D:\Data\Monica Rincon\Francisco Cortes Benitez\20240819_DLC-3-102.d	Acquisition Date	8/21/2024 2:22:08 AM
Method	Tune Low extendido.m	Operator	Admin
Sample Name	20240819_DLC-3-102	Instrument	micrOTOF 213750.00410
Comment			

Acquisition Parameter

Source Type	ESI	Ion Polarity	Positive	Set Nebulizer	0.5 Bar
Focus	Active			Set Dry Heater	150 °C
Scan Begin	50 m/z	Set Capillary	4500 V	Set Dry Gas	4.0 l/min
Scan End	3000 m/z	Set End Plate Offset	-500 V	Set Divert Valve	Waste

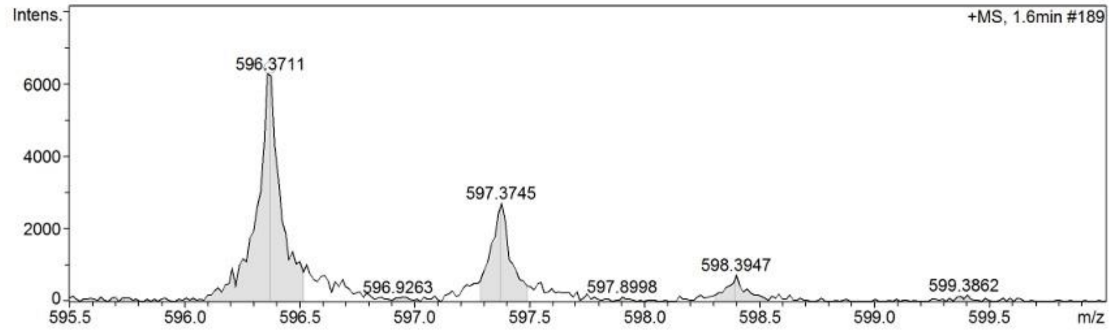

Meas. m/z	#	Ion Formula	m/z	err [ppm]	|err| [mDa]	z	mSigma	# mSigma	e⁻ Conf
596.3711	1	C37H49F3NO2	596.3710	-0.2	0.1	1+	45.0	1	even

Figure S45. Mass spectrum formula report of compound **3a.**

Mass Spectrum SmartFormula Report

Analysis Info

Analysis Name	D:\Data\Monica Rincon\Francisco Cortes Benitez\202300824_DLC-3-38.d	Acquisition Date	8/24/2023 3:48:29 PM
Method	Tune Low extendido.m	Operator	Admin
Sample Name	202300824_DLC-3-38	Instrument	micrOTOF 213750.00410
Comment			

Acquisition Parameter

Source Type	ESI	Ion Polarity	Positive	Set Nebulizer	0.5 Bar
Focus	Active			Set Dry Heater	150 °C
Scan Begin	50 m/z	Set Capillary	4500 V	Set Dry Gas	4.0 l/min
Scan End	3000 m/z	Set End Plate Offset	-500 V	Set Divert Valve	Waste

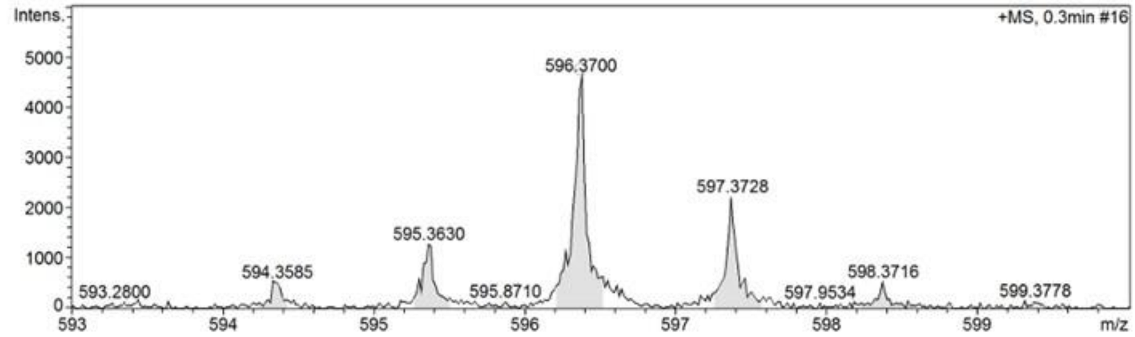

Meas. m/z	#	Ion Formula	Sum Formula	m/z	err [ppm]	|err| [mDa]	mSigma	# mSigma
596.3700	1	C37H49F3NO2	C37H48F3NO2	596.3710	1.6	1.0	38.0	1

Figure S46. Mass spectrum formula report of compound **3b**.

Mass Spectrum SmartFormula Report

Analysis Info

Analysis Name	D:\Data\Monica Rincon\Francisco Cortes Benitez\202300824_DLC-3-92.d	Acquisition Date	8/24/2023 4:10:28 PM
Method	Tune Low extendido.m	Operator	Admin
Sample Name	202300824_DLC-3-92	Instrument	micrOTOF 213750.00410
Comment			

Acquisition Parameter

Source Type	ESI	Ion Polarity	Positive	Set Nebulizer	0.5 Bar
Focus	Active			Set Dry Heater	150 °C
Scan Begin	50 m/z	Set Capillary	4500 V	Set Dry Gas	4.0 l/min
Scan End	3000 m/z	Set End Plate Offset	-500 V	Set Divert Valve	Waste

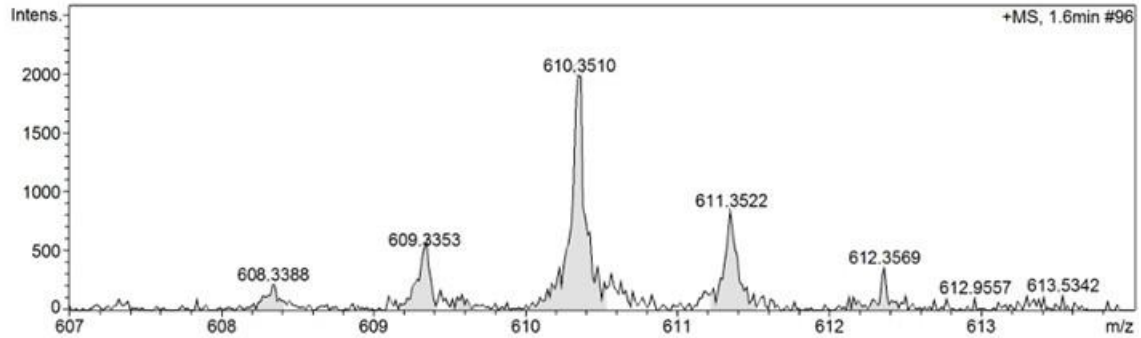

Meas. m/z	#	Ion Formula	Sum Formula	m/z	err [ppm]	|err| [mDa]	mSigma	# mSigma
610.3510	1	C37H47F3NO3	C37H46F3NO3	610.3503	-1.2	0.7	47.0	1

Figure S47. Mass spectrum formula report of compound **3c**.

Mass Spectrum SmartFormula Report

Analysis Info

Analysis Name	D:\Data\Monica Rincon\Francisco Cortes Benitez\20240819_DLC-3-104 .d	Acquisition Date	8/21/2024 2:38:10 AM
Method	Tune Low extendido.m	Operator	Admin
Sample Name	20240819_DLC-3-98	Instrument	micrOTOF 213750.00410
Comment			

Acquisition Parameter

Source Type	ESI	Ion Polarity	Positive	Set Nebulizer	0.5 Bar
Focus	Active			Set Dry Heater	150 °C
Scan Begin	50 m/z	Set Capillary	4500 V	Set Dry Gas	4.0 l/min
Scan End	3000 m/z	Set End Plate Offset	-500 V	Set Divert Valve	Waste

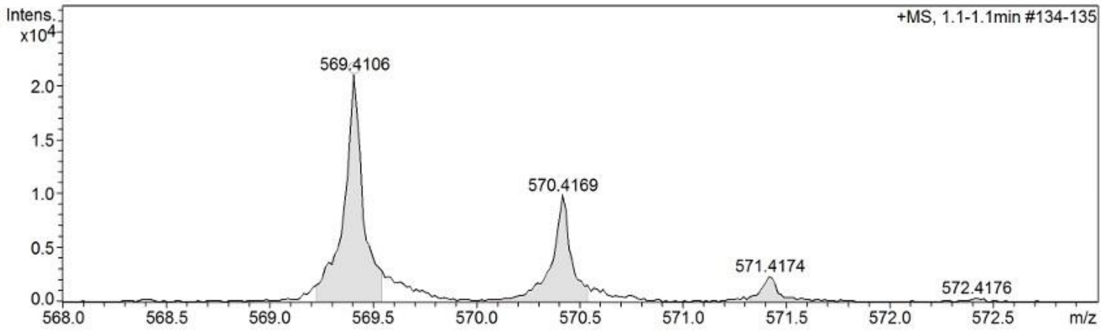

Meas. m/z	#	Ion Formula	Sum Formula	m/z	err [ppm]	|err| [mDa]	z	mSigma	# mSigma	e⁻ Conf
569.4106	1	C38H53N2O2	C38H52N2O2	569.4102	-0.8	0.4	1+	24.4	1	even

Figure S48. Mass spectrum formula report of compound **5a**.

Mass Spectrum SmartFormula Report

Analysis Info

Analysis Name	D:\Data\Monica Rincon\Francisco Cortes Benitez\20240819_DLC-3-98.d	Acquisition Date	8/21/2024 1:59:45 AM
Method	Tune Low extendido.m	Operator	Admin
Sample Name	20240819_DLC-3-98	Instrument	micrOTOF 213750.00410
Comment			

Acquisition Parameter

Source Type	ESI	Ion Polarity	Positive	Set Nebulizer	0.5 Bar
Focus	Active			Set Dry Heater	150 °C
Scan Begin	50 m/z	Set Capillary	4500 V	Set Dry Gas	4.0 l/min
Scan End	3000 m/z	Set End Plate Offset	-500 V	Set Divert Valve	Waste

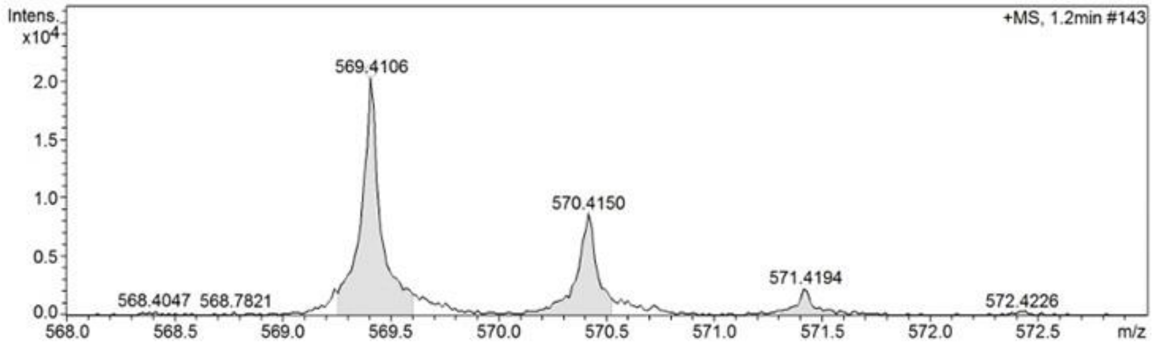

Meas. m/z	#	Ion Formula	Sum Formula	m/z	err [ppm]	|err| [mDa]	z	mSigma	# mSigma	e⁻ Conf
569.4106	1	C38H53N2O2	C38H52N2O2	569.4102	-0.7	0.4	1+	10.6	1	even

Figure S49. Mass spectrum formula report of compound **5b**.

Mass Spectrum SmartFormula Report

Analysis Info

Analysis Name	D:\Data\Monica Rincon\Francisco Cortes Benitez\20230804_DLC-3-91_.d	Acquisition Date	8/4/2023 5:15:35 PM
Method	Tune Low extendido.m	Operator	Admin
Sample Name	20230804_DLC-3-91_	Instrument	micrOTOF 213750.00410
Comment			

Acquisition Parameter

Source Type	ESI	Ion Polarity	Positive	Set Nebulizer	0.5 Bar
Focus	Active			Set Dry Heater	150 °C
Scan Begin	50 m/z	Set Capillary	4500 V	Set Dry Gas	4.0 l/min
Scan End	3000 m/z	Set End Plate Offset	-500 V	Set Divert Valve	Waste

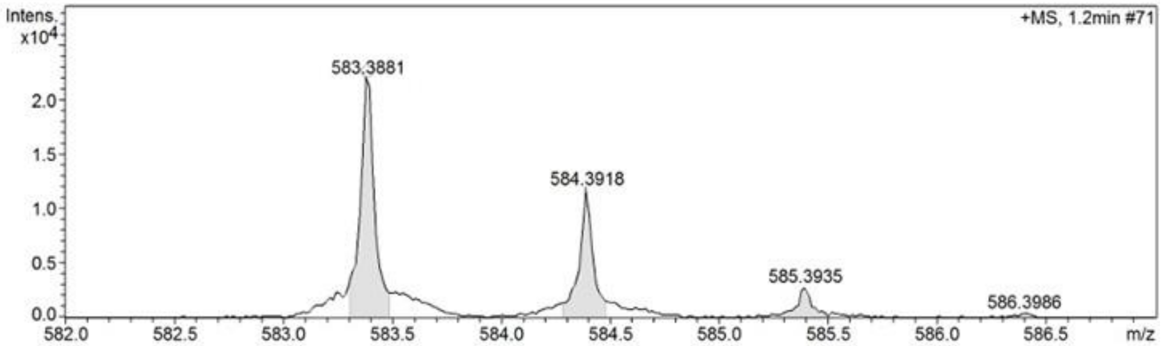

Meas. m/z	#	Ion Formula	Sum Formula	m/z	err [ppm]	|err| [mDa]	mSigma	# mSigma
583.3881	1	C38H51N2O3	C38H50N2O3	583.3894	2.3	1.3	56.7	1

Figure S50. Mass spectrum formula report of compound **5c**.

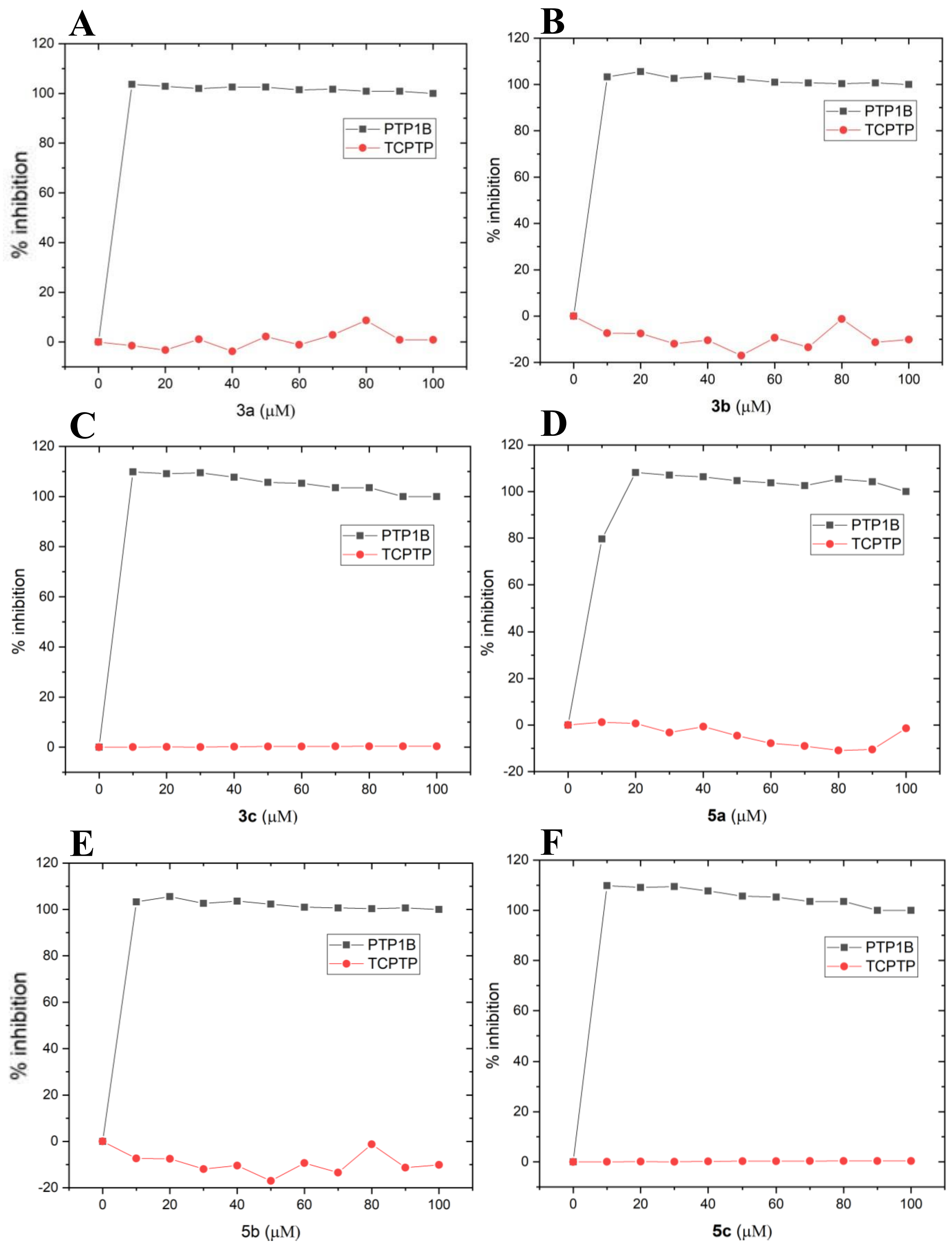
A
% inhibition
PTP1B
TCPTP
3a (μM)
B
% inhibition
PTP1B
TCPTP
3b (μM)
C
% inhibition
PTP1B
TCPTP
3c (μM)
D
% inhibition
PTP1B
TCPTP
5a (μM)
E
% inhibition
PTP1B
TCPTP
5b (μM)
F
% inhibition
PTP1B
TCPTP
5c (μM)

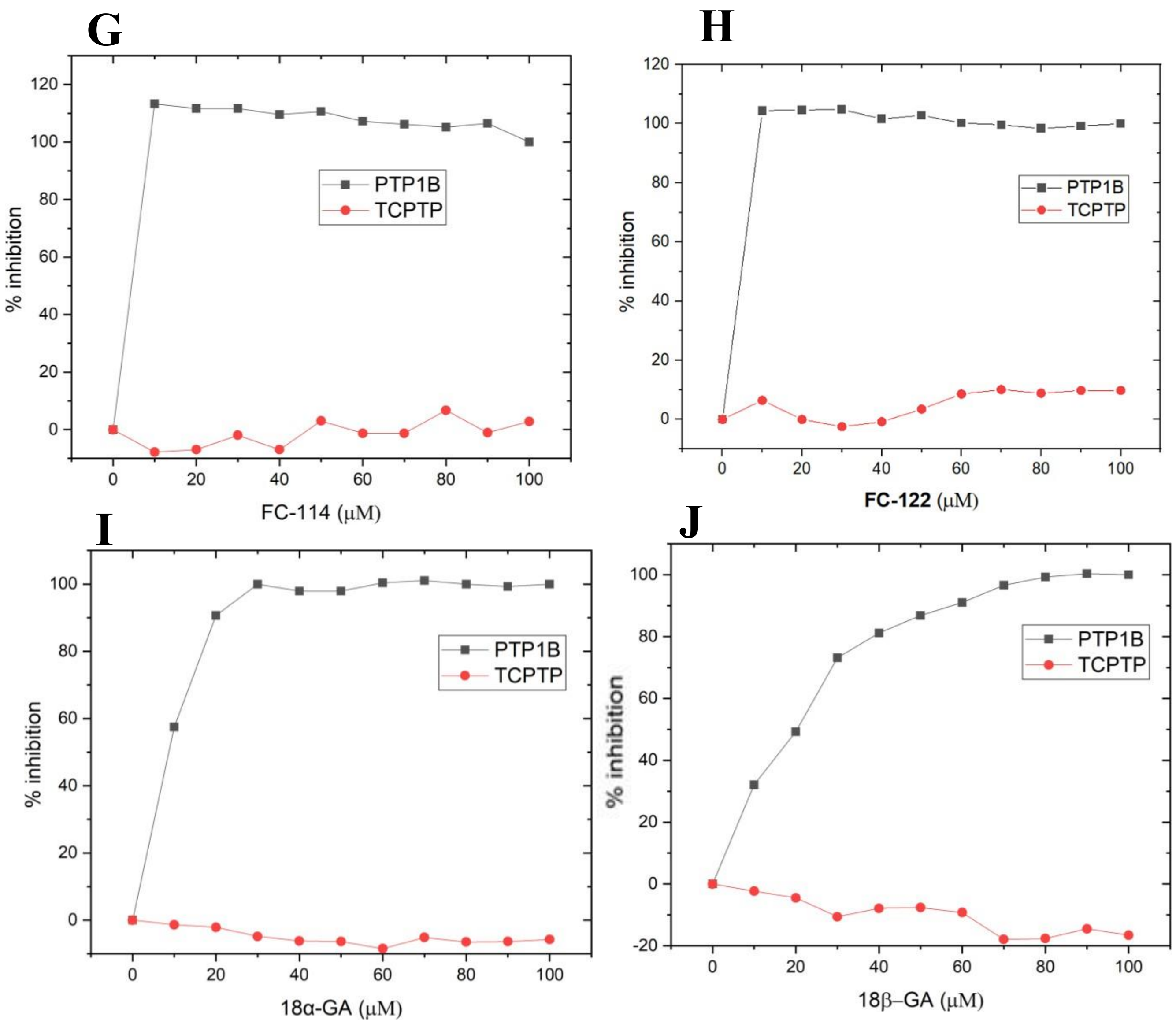

Figure S51. Selectivity of the inhibition for PTP1B over TCPTP for GA and its derivatives at 100 μM. **(A)** compound **3a**; **(B)** compound **3b**; **(C)** compound **3c**; **(D)** compound **5a**; **(E)** compound **5b**; **(F)** compound **5c; (G)** FC-114; **(H)** FC-122; **(I)** 18α-GA; **(J)** 18β-GA.

A

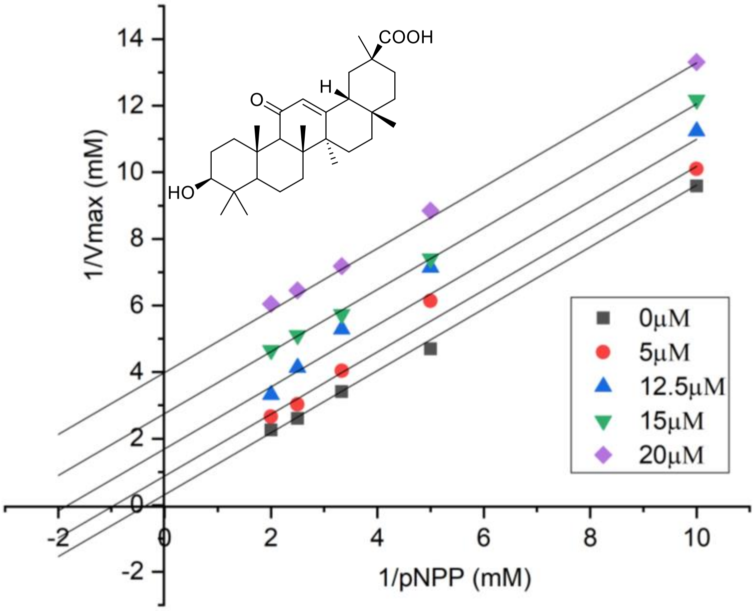
COOH
H
O
HO
1/Vmax (mM)
1/pNPP (mM)
0μM
5μM
12.5μM
15μM
20μM
14
12
10
8
6
4
2
0
-2
-2
0
2
4
6
8
10

B

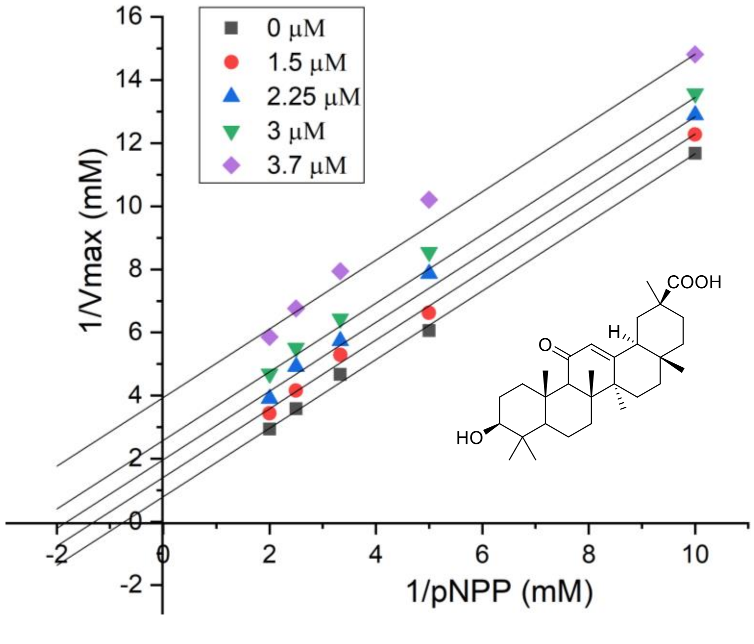
0 μM
1.5 μM
2.25 μM
3 μM
3.7 μM
COOH
H
O
HO
1/Vmax (mM)
1/pNPP (mM)
16
14
12
10
8
6
4
2
0
-2
-2
0
2
4
6
8
10

C

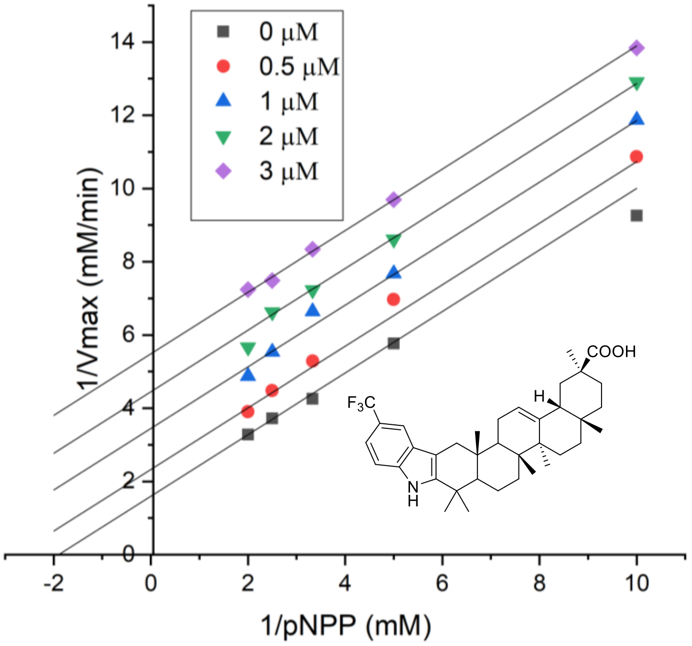

0 μM
0.5 μM
1 μM
2 μM
3 μM
1/Vmax (mM/min)
1/pNPP (mM)
COOH
H
F_3C
N
H

D

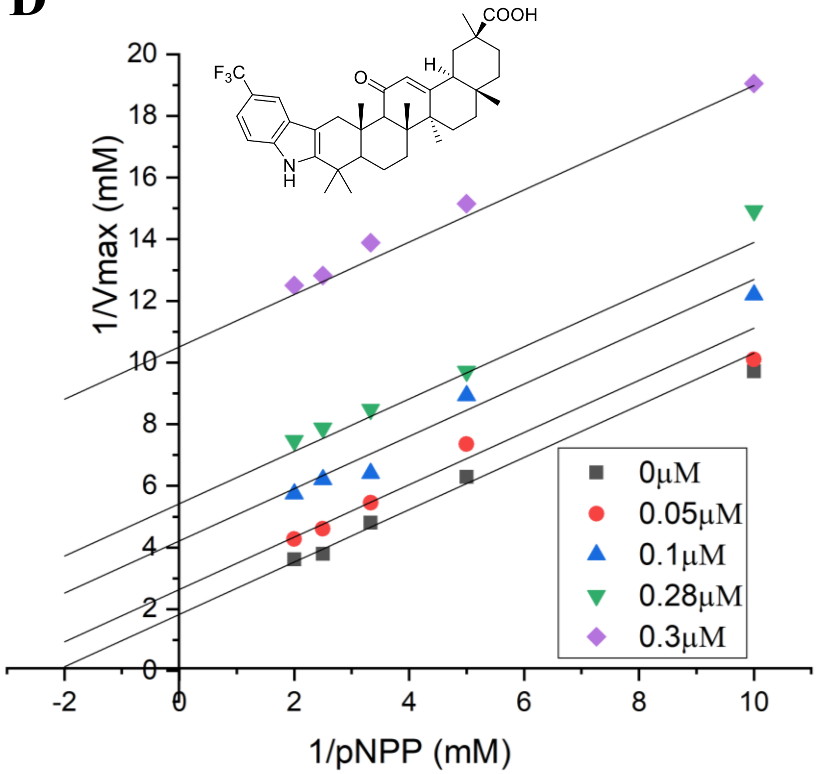

COOH
F_3C
O
H
N
H
1/Vmax (mM)
0μM
0.05μM
0.1μM
0.28μM
0.3μM
1/pNPP (mM)

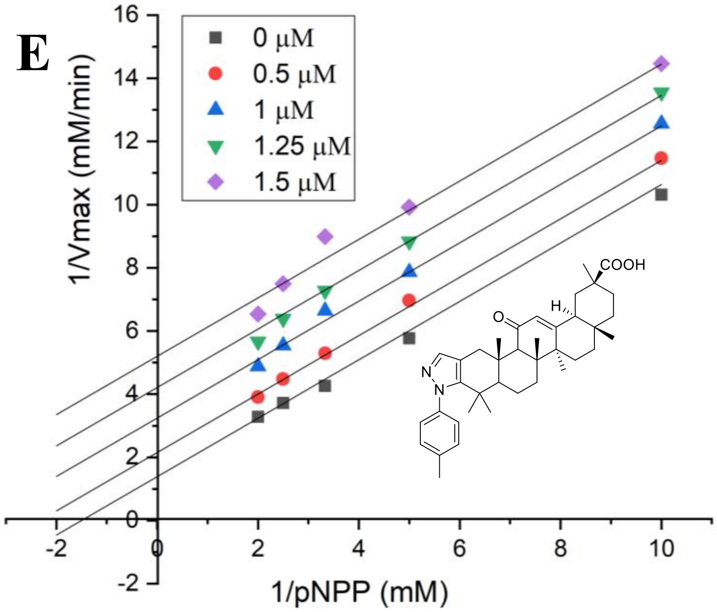

Figure S52. Lineweaver–Burk plots for *h*PTP1B$_{1\text{-}400}$ inhibition by 18β-GA (**A**), 18α-GA (**B**), compound **3a** (**C**), compound **3c** (**D**), and compound **5c** (**E**).

Equation 1 was used to obtain enzyme kinetic parameters by fitting data to the Michaelis-Menten model (Origin Pro 2018 (64 bit) SR1).

$$y - \frac{V_{max}\, i}{K_m + i} \qquad \textbf{E1}$$

The equations defined for the following non-linear inhibition models were used to determine the mechanism of PTP1B inhibition: competitive (E2), non-competitive (E3), uncompetitive (E4), and mixed (E5) (Origin Pro 2018 (64 bit) SR1).

$$y = \frac{V_{max}\,(x)}{K_m\left(1+\left(\frac{i}{Ki}\right)\right)+x} \qquad \textbf{E2}$$

$$y = V_{max} \frac{x^{nh}}{\left(1+\left(\frac{i}{K_i}\right)\right)(x_{05}nh)+\left(1+\left(\frac{i}{K_i}\right)\right)(x_{05}nh)} \quad \textbf{E3}$$

$$y = \frac{\mathrm{V_{max}(x)}}{((\mathrm{K_m})/(\frac{1+i}{\mathrm{K_I}}))+x} \quad \textbf{E4}$$

$$y = \frac{((V_{max}\,(x))/(\frac{1+i}{\alpha \mathrm{K_I}}))}{x+\mathrm{K_m}((\frac{1+i}{\mathrm{K_I}})/(\frac{1+i}{\alpha \mathrm{K_I}}))} \quad \textbf{E5}$$

Vmax is the maximum velocity, x is the substrate concentration, i is the inhibitor concentration, and nh is the number of Hill. Ki is the inhibition constant, and Km is the Michaelis constant.

Table S3. Quality parameters of the generated $PTP1B_{1-400}$ model provided by the *Molprobity* server.

All-atom contacts	Clashscore, all atoms:	0.78		99th percentile * (N=1784, all resolutions)
	Clashscore is the number of serious steric overlaps (> 0.4Å) per 1000 atoms			
Protein geometry	Poor rotamers	5	1.39%	Goal: <0.3%
	Favored rotamers	350	97.22%	Goal: >98%
	Ramachandran outliers	1	0.25%	Goal: <0.05%
	Ramachandran favored	384	96.48%	Goal: >98%
	Rama distribution Z-score	-0.23±0.38		Goal: abs(Z score) < 2
	MolProbity score	1.08		100th percentile * (N=27675, 0Å–99Å)
	Cβ deviations >0.25Å	0	0.00%	Goal: 0
	Bad bonds:	6/3310	0.18%	Goal: 0%
	Bad angles:	6/4472	0.13%	Goal: <0.1%
Peptide Omegas	Cis prolines:	4/29	13.79%	Expected: ≤1 per chain, or ≤5%
	Cis nonProlines:	3/370	0.81%	Goal: <0.05%
	Twisted Peptides:	1/399	0.25%	
Low-resolution criteria	CaBLAM outliers	6	1.5%	Goal: <1.0%
	CA geometry outliers	3	0.76%	Goal: <0.5%
Additional validations	Chiral volume outliers	0/466		
	Waters with clashes	0/0	0.00%	See UnDowser table for details

In the two-column results, the left column displays the raw count, and the right column shows the corresponding percentage.

* 100th percentile is the best among structures of comparable resolution; 0th percentile is the worst.

For clashscore, the comparative set of structures was selected in 2004, for MolProbity score in 2006.

^ MolProbity score combines the clashscore, rotamer, and Ramachandran evaluations into a single score, normalized to be on the same scale as X-ray resolution.

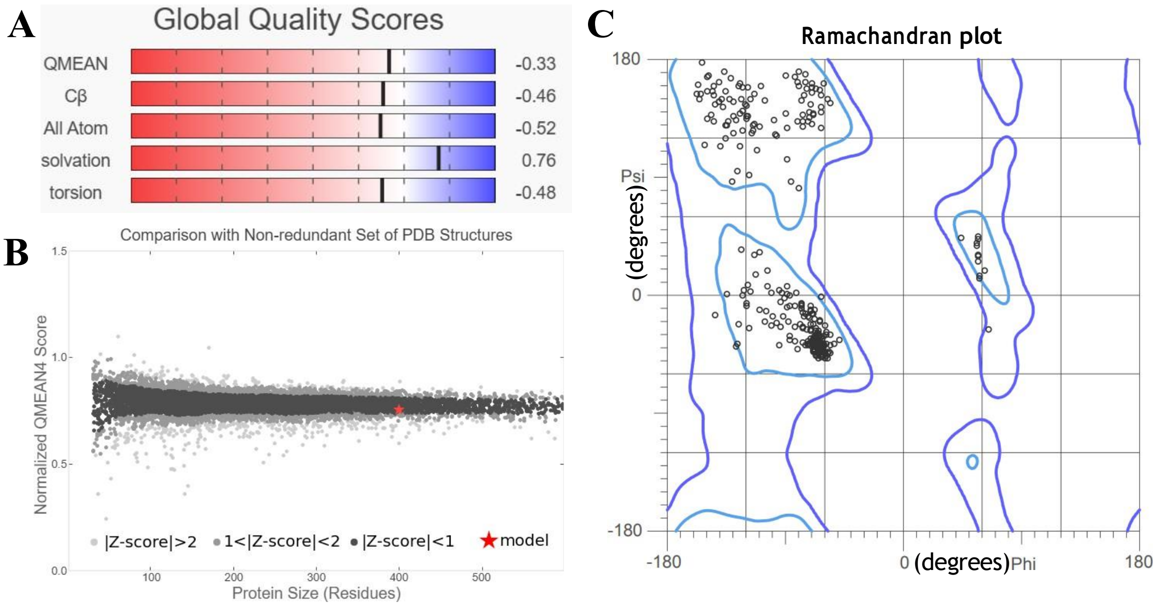

Figure S53. Validation of the PTP1B$_{1-400}$ model. (A) Representation of QMEAN score and its four terms. (B) Comparison of the Normalized QMEAN4 score of the Ramachandran plot of the PTP1B$_{1-400}$ modeled against a non-redundant set of PDB structures. (C) Ramachandran plot of the PTP1B$_{1-400}$ model.

Table S4. Quality parameters for the $TCPTP_{1\text{-}415}$ model provided by the *Molprobity* server.

All-atom contacts	Clashscore, all atoms:	0		99th percentile * (N=1784, all resolutions)
	Clashscore is the number of serious steric overlaps (> 0.4Å) per 1000 atoms			
Protein geometry	Poor rotamers	1	0.26%	Goal: <0.3%
	Favored rotamers	375	99.21%	Goal: >98%
	Ramachandran outliers	1	0.24%	Goal: <0.05%
	Ramachandran favored	402	97.34%	Goal: >98%
	Rama distribution Z-score	0.98±0.38		Goal: abs(Z score) < 2
	MolProbity score	0.63		100th percentile * (N=27675, 0Å–99Å)
	Cβ deviations >0.25Å	0	0.00%	Goal: 0
	Bad bonds:	4/3482	0.11%	Goal: 0%
	Bad angles:	7/4698	0.15%	Goal: <0.1%
Peptide Omegas	Cis prolines:	0/17	0.00%	Expected: ≤1 per chain, or ≤5%
	Cis nonProlines:	1/397	0.25%	Goal: <0.05%
Low-resolution criteria	CaBLAM outliers	4	1.0%	Goal: <1.0%
	CA geometry outliers	2	0.49%	Goal: <0.5%
Additional validations	Chiral volume outliers	0/500		
	Waters with clashes	0/0	0.00%	See UnDowser table for details

In the two-column results, the left column gives the raw count, and the right column shows the percentage.

* 100th percentile is the best among structures of comparable resolution; 0th percentile is the worst.

For clashscore, the comparative set of structures was selected in 2004, for MolProbity score in 2006.

^ MolProbity score combines the clashscore, rotamer, and Ramachandran evaluations into a single score, normalized to be on the same scale as X-ray resolution.

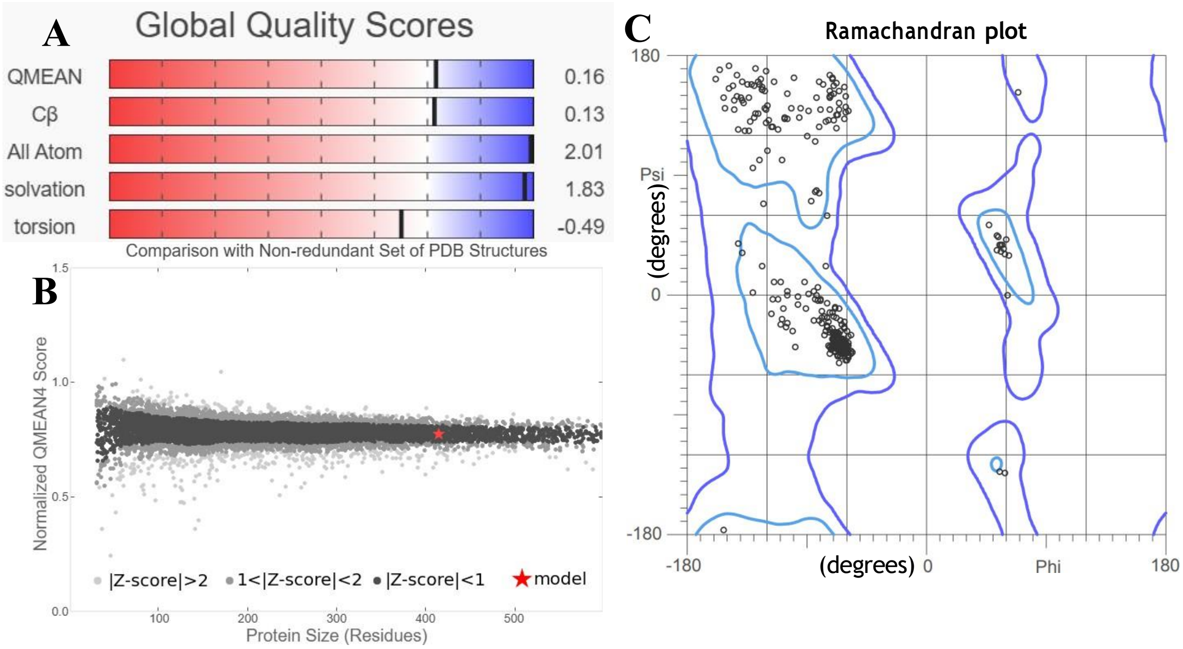

Figure S54. Validation of the TCPTP$_{1\text{-}415}$ model. (A) Representation of QMEAN score and its four terms. (B) Comparison of the Normalized QMEAN4 score of the Ramachandran plot of the TCPTP$_{1\text{-}415}$ modeled against a non-redundant set of PDB structures. (C) Ramachandran plot of the TCPTP$_{1\text{-}415}$ model.

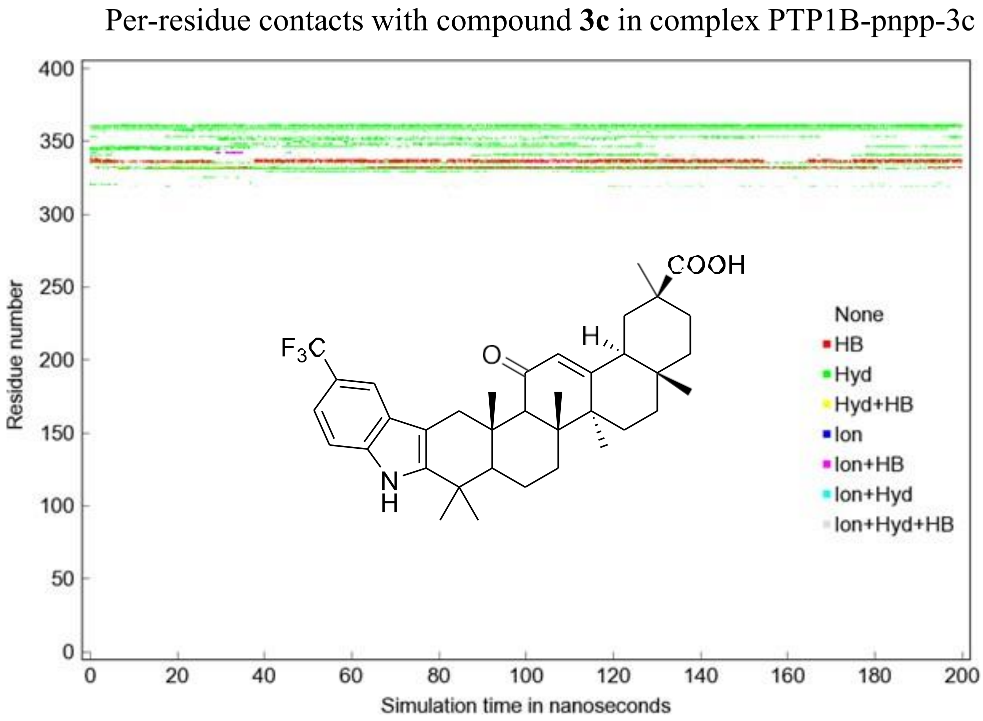

Figure S55. Per-residue contacts with compound **3c** during 200 ns MD simulation.

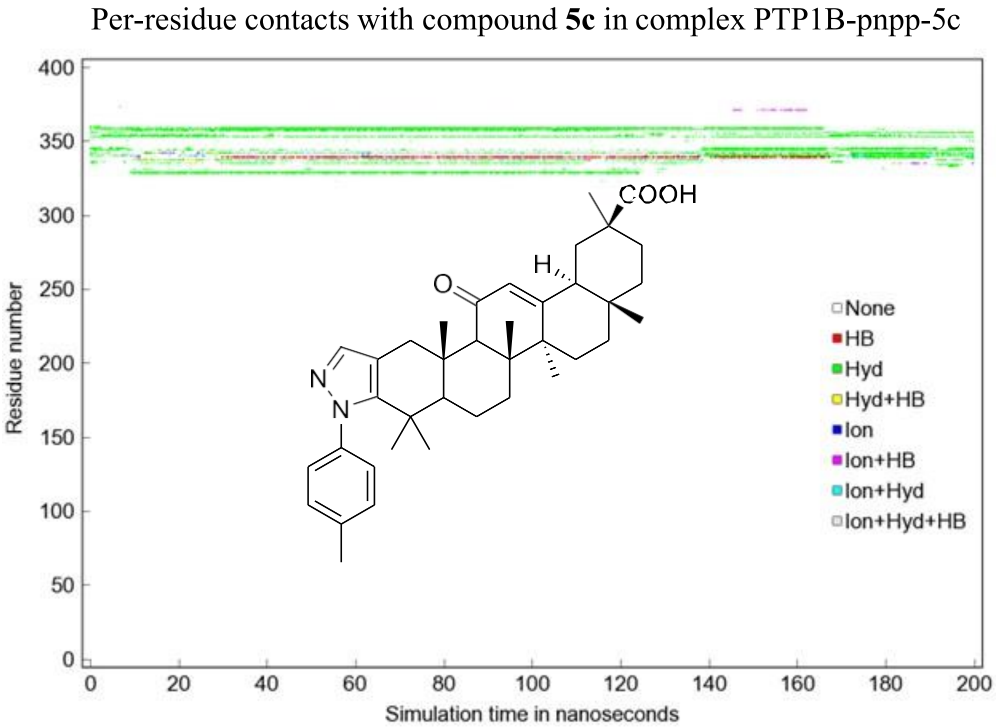

Figure S56. Per-residue contacts with compound **5c** during 200 ns MD simulation.

Per-residue contacts with compound **FC-114** in complex PTP1B-pnpp-FC-114

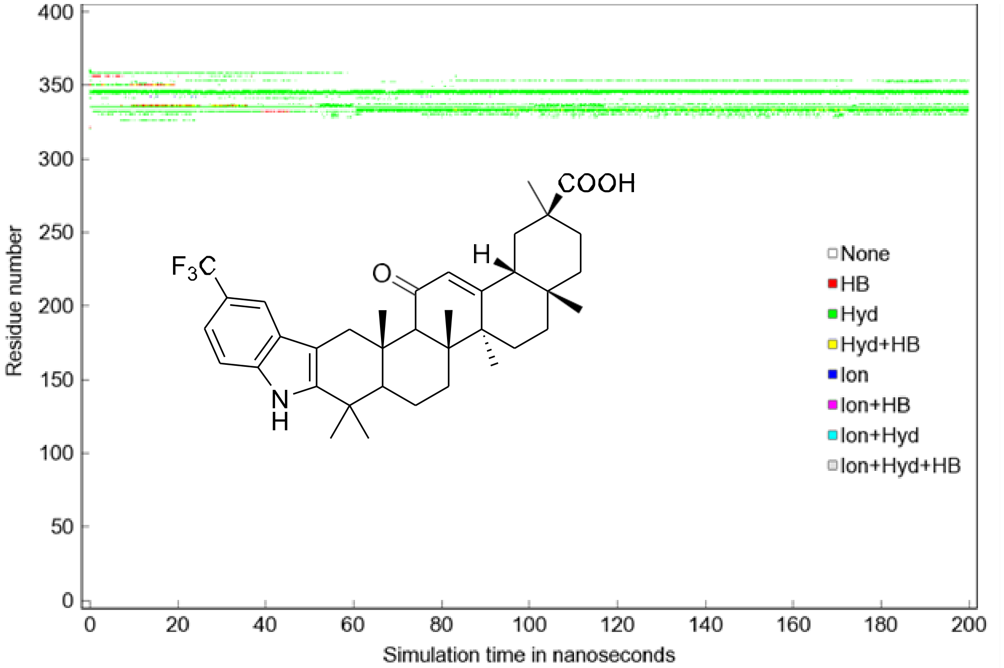

Figure S57. Per-residue contacts with compound **FC-114** during 200 ns MD simulation.

Per-residue contacts with compound **FC-122** in complex PTP1B-pnpp-FC-122

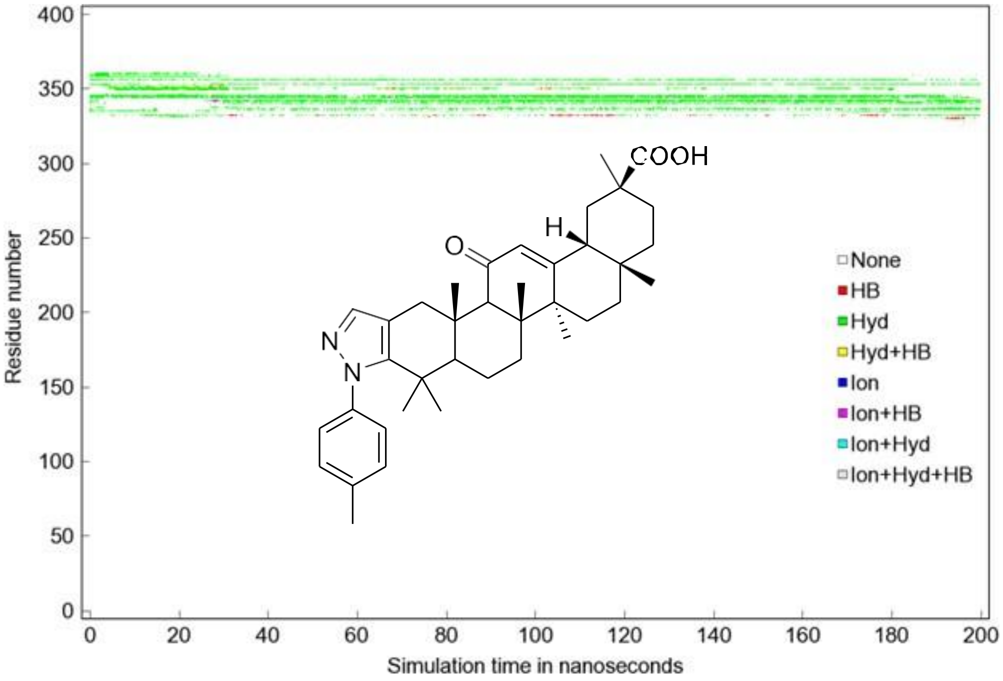

Figure S58. Per-residue contacts with compound **FC-122** during 200 ns MD simulation.

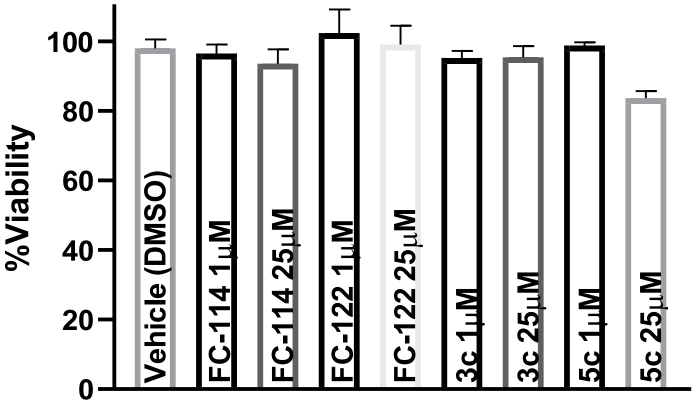

Figure S59. Cell viability assessment by crystal violet staining. HepG2 cell viability was evaluated after treatment with the respective compounds at concentrations of 1 and 25 μM for 24 hours. Bar graphs represent mean ± SEM. No significant differences were detected using ANOVA.
